# 3D genome organization in the epithelial-mesenchymal transition spectrum

**DOI:** 10.1186/s13059-022-02687-x

**Published:** 2022-05-30

**Authors:** Qing You Pang, Tuan Zea Tan, Vignesh Sundararajan, Yi-Chia Chiu, Edward Yu Wing Chee, Vin Yee Chung, Mahesh A. Choolani, Ruby Yun-Ju Huang

**Affiliations:** 1 Department of Obstetrics & Gynaecology, Yong Loo Lin School of Medicine, National University Health System, Singapore, 119077 Singapore; 2 Cancer Science Institute of Singapore, National University of Singapore, Center for Translational Medicine, Singapore, 117599 Singapore; 3 Genomics and Data Analytics Core (GeDaC), Cancer Science Institute of Singapore, National University of Singapore, 14 Medical Drive, #12-01, Singapore, 117599 Singapore; 4 School of Medicine, College of Medicine, National Taiwan University, No. 1, Ren-Ai Road Section I, Taipei, 10051 Taiwan; 5 Graduate Institute of Oncology, College of Medicine, National Taiwan University, Taipei, 10051 Taiwan

## Abstract

**Background:**

The plasticity along the epithelial-mesenchymal transition (EMT) spectrum has been shown to be regulated by various epigenetic repertoires. Emerging evidence of local chromatin conformation changes suggests that regulation of EMT may occur at a higher order of three-dimensional genome level.

**Results:**

We perform Hi-C analysis and combine ChIP-seq data across cancer cell lines representing different EMT states. We demonstrate that the epithelial and mesenchymal genes are regulated distinctively. We find that EMT genes are regulated within their topologically associated domains (TADs), with only a subset of mesenchymal genes being influenced by A/B compartment switches, indicating topological remodeling is required in the transcriptional regulation of these genes. At the TAD level, epithelial and mesenchymal genes are associated with different regulatory trajectories. The epithelial gene-residing TADs are enriched with H3K27me3 marks in the mesenchymal-like states. The mesenchymal gene-residing TADs, which do not show enrichment of H3K27me3 in epithelial-like states, exhibit increased interaction frequencies with regulatory elements in the mesenchymal-like states.

**Conclusions:**

We propose a novel workflow coupling immunofluorescence and dielectrophoresis to unravel EMT heterogeneity at single-cell resolution. The predicted three-dimensional structures of chromosome 10, harboring Vimentin, identify cell clusters of different states. Our results pioneer a novel avenue to decipher the complexities underlying the regulation of EMT and may infer the barriers of plasticity in the 3D genome context.

**Supplementary Information:**

The online version contains supplementary material available at 10.1186/s13059-022-02687-x.

## Background

Epithelial-mesenchymal transition (EMT) is a biological process that is fundamental to embryonic development and wound healing; however, it is also hijacked by cancer cells to acquire an invasive and aggressive phenotype to enable metastasis during tumor progression [1]. The morphological changes during EMT are briefly described as the transition from cobblestone, polarized epithelial cells into a dispersed spindle-shaped mesenchymal phenotype, accompanied by the corresponding switch between epithelial and mesenchymal markers [2–6]. The recent paradigm of EMT has shifted from the binary to the spectral view consisting of various intermediate states [1, 7]. Cancer cells undergo EMT in a sequential fashion resulting in diverse populations of differing EMT states—from a state enriched in epithelial markers, to one that expresses both epithelial/mesenchymal markers and lastly in a state where only mesenchymal markers are expressed [8, 9]. The transitional phases of the EMT states have thus been described as an EMT spectrum [10, 11] and shown in in vivo models [12, 13].

The plasticity along the EMT spectrum is regulated by transcriptional programs consisting of a repertoire of different transcription factors (TFs) between each state [1, 11, 12]. These TFs form a core regulatory network to either stabilize a given state or to drive the plasticity [1, 2, 6, 14–16]. The EMT-TFs network modulates the expression of EMT genes at various levels, through direct transcriptional control, epigenetic modifications, alternative splicing, post-translational modifications, subcellular localizations, and non-coding RNA regulation [1, 17, 18]. The interest in the epigenomic landscape changes in EMT has been growing, given its possible role in conferring the fluid nature of EMT in cancer [17, 19, 20] similar to the reprogramming during cell fate determination [21]. The epigenetic remodeling regulated by a pioneer factor-like EMT suppressor, grainyhead-like 2 (*GRHL2*), exemplified the fluidity and barriers that might exist in terms of the plasticity along the EMT spectrum [22, 23]. The diverse predicted enrichment of TF binding sites at the open chromatin regions among different states [12] also pointed to a non-linear scenario of the plasticity landscape to regulate the EMT genes.

Studies on selected loci of epithelial or mesenchymal genes have implied that local chromatin conformation changes are crucial during EMT [24–27]. For instance, the transcription of *CDH1* was shown to be mediated via DNA looping between GRHL2 and HNFα enhancers in an EMT model using mouse mammary epithelial cells [24]. A more recent study on a TGF-β-induced EMT model showed that transcriptionally active chromatin in the “active” compartments can interact with the heterochromatic lamin filament proteins lamin B1 during phenotype transition [27]. This emerging evidence of local chromatin conformation changes is implicating that the regulation of EMT genes might occur at a higher order at the three-dimensional (3D) genome level.

The 3D conformation of chromatin has important roles in the transcriptional regulation of genes [28]. Chromatin conformation capture-based methods have revealed the hierarchical organization of the chromatin architecture–chromosome territories, active/inactive (A/B) chromosomal compartments, topological associating domains (TADs), and short- and long-range chromatin interactions [29–32]. The reorganization of the 3D chromatin conformation is evident in influencing cell identity during lineage-differentiation and can be dysregulated in diseases [33, 34]. Despite plethora of studies describing the 3D genome in various basic biological processes, such as cell cycle and development or in diseases [33, 35–37], there is no study to date to investigate the changes in the 3D chromatin structure among the epithelial and mesenchymal plasticity states.

In this study, we analyzed genome-wide higher-order chromatin structure in cancer cell lines of epithelial and mesenchymal states through the integration of Hi-C, histone ChIP-seq, and RNA-seq data from both our in-house platforms and the ENCODE (Encyclopedia of DNA Elements) dataset [38, 39]. The genome-wide integrative analyses aimed to investigate variability of chromatin conformation and chromatin state changes during EMT in relation to gene transcription. Furthermore, to dissect the underlying heterogeneity, we combined fluorescence labelling of the lineage markers and a dielectrophoresis approach to isolate cells representing differing EMT states to deconvolute the chromatin conformation changes at the single-cell resolution. This study provided the most extensive data for the analysis of higher-order chromatin structure along the EMT plasticity spectrum in cancer cells to date.

## Results

### Refinement of a subset of pan-cancer epithelial and mesenchymal genes

**Fig. 1:**
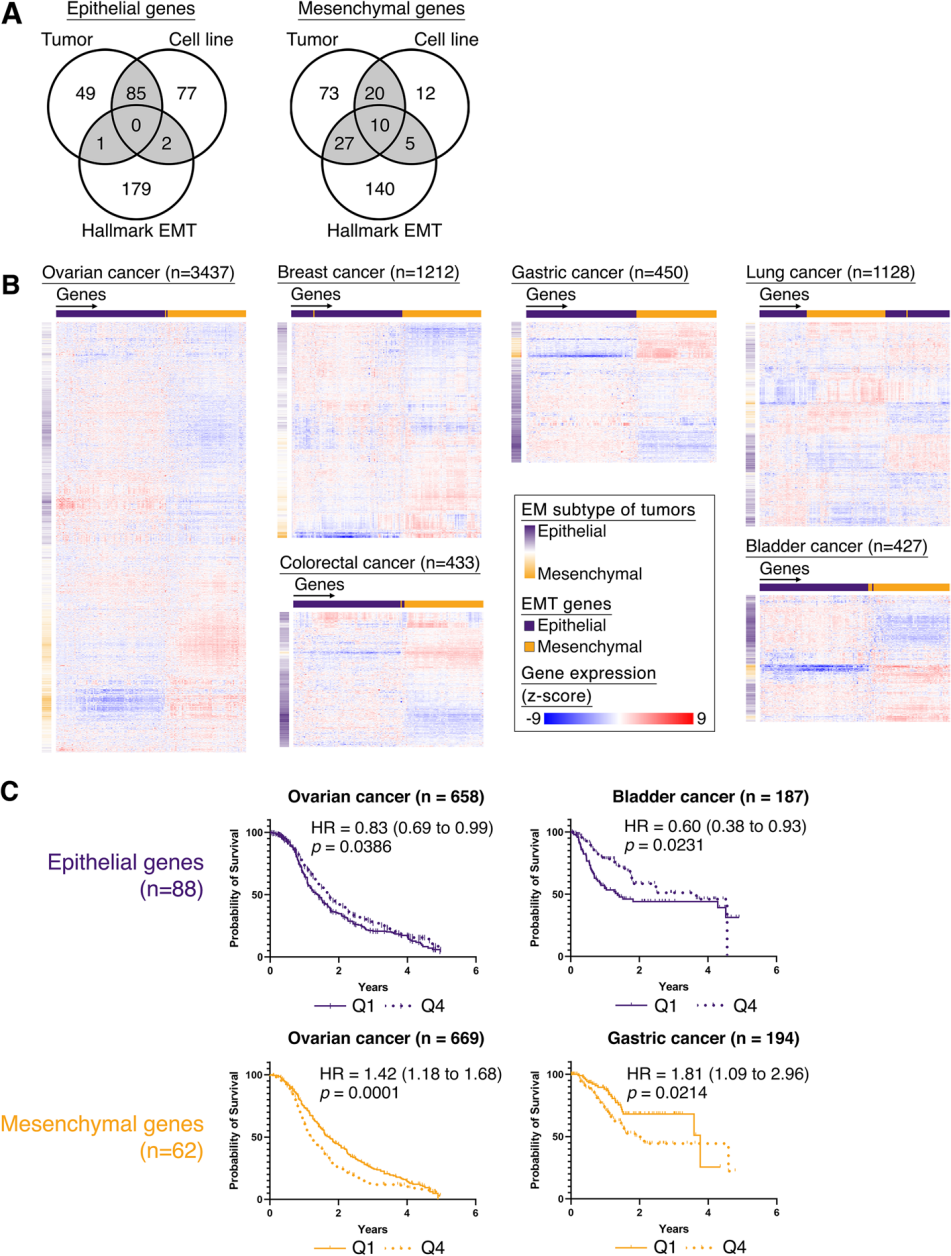
**a** Venn diagram of overlapping epithelial and mesenchymal genes of generic EMT signatures from tumor and cell line, with hallmark EMT genes. **b** Heatmaps of hierarchical clustering the expression of epithelial (purple) and mesenchymal (orange) genes (horizontal) across tumors (vertical) in ovarian, breast, gastric, lung, colorectal, and bladder cancers with the EMT scores of the tumors depicted vertically alongside the heatmap, indicating the EM subtypes. **c** Kaplan-Meier survival analysis of epithelial (purple) or mesenchymal (orange) genes for 1st quartile (solid line) vs 4th quartile (dotted line), in ovarian, bladder, and gastric cancers

To identify a core set of EMT genes, we refined a subset of pan-cancer epithelial (E) and mesenchymal (M) genes by using previously published generic EMT gene signatures for cell lines and tumors [10] and overlapped with the hallmark EMT signature of the Molecular Signatures Database (MSigDB v7.4) [40] to obtain 88 epithelial and 62 mesenchymal genes (Fig. 1A). We first verified if the expression of these E and M genes were still able to stratify the tumors by their EMT status across multiple cancer types. The EMT states of the tumors, in each cancer dataset, were scored by our refined generic signature, which had the advantage of being able to quantify EMT status in a pan-cancer fashion, while simultaneously unbiased towards EMT-related stromal genes [10]. We observed that the E-scored tumors had a stronger expression of E genes and a reduced expression of the M genes in 6 cancers, namely ovarian, breast, gastric, lung, colorectal, and bladder cancer (Fig. 1B). In particular, the tumors which had a hybrid EM state showed a distinct differential expression in both the E and M genes. These EMT genes were not frequently associated with genomic alterations (Average mutation frequency = 0.7%; Additional file 2: Fig. S1A) nor showed frequent copy number gains/losses (average copy number gain = 1%, average copy number loss = 0.3%; Additional file 2: Fig. S1FA) in pan-cancer TCGA tumors (*n *= 8510), indicating that the regulation of these EMT genes in cancer was not a consequence of genomic alterations. To investigate if this refined subset of EMT genes were associated with disease-free survival (DFS), Kaplan–Meier analyses was performed on the E and M genes across 6 cancers (Fig. 1C, Additional file 2: Fig. S1B). The E genes were associated with better survival in ovarian (HR = 0.83, *p* = 0.0386; Fig. 1C) and bladder cancers (HR = 0.60, *p* = 0.0231; Fig. 1C), while patients with tumors that exhibit higher expression of the M genes (Q1) showed poorer DFS in ovarian (HR= 1.42, *p* = 0.0001; Fig. 1C) and gastric cancer (HR = 1.81, *p* = 0.0214; Fig. 1C). Interestingly, the tumors with higher expression of M genes in bladder and colorectal cancers also showed poorer survival, despite not showing significance (bladder cancer – HR = 1.6, *p* = 0.052; colorectal cancer – HR = 1.5, *p* = 0.1428; Additional file 2: Fig. S1B), which could be due to the small sample sizes. While for breast and lung cancers, the EMT genes were not associated with survival (breast cancer – HR_epithelial_ = 0.94, HR_mesenchymal_ = 1.20; lung cancer – HR_epithelial_ = 1.34, HR_mesenchymal_ = 1.1; Fig. S1B). In summary, the 88 E and 62 M genes are only likely to determine the patient prognosis in selected cancer types, as the survival could be affected by the cancer-specific microenvironments and molecular subtypes. The varying association of EMT genes to prognosis was also evident in previous studies [10, 41]. Nevertheless, the refined subset of EMT genes presented in this study were able to clearly stratify the tumors by their EMT states in multiple cancers.

### A subset of mesenchymal genes exhibited concordant compartmental switch between EM states

**Fig. 2:**
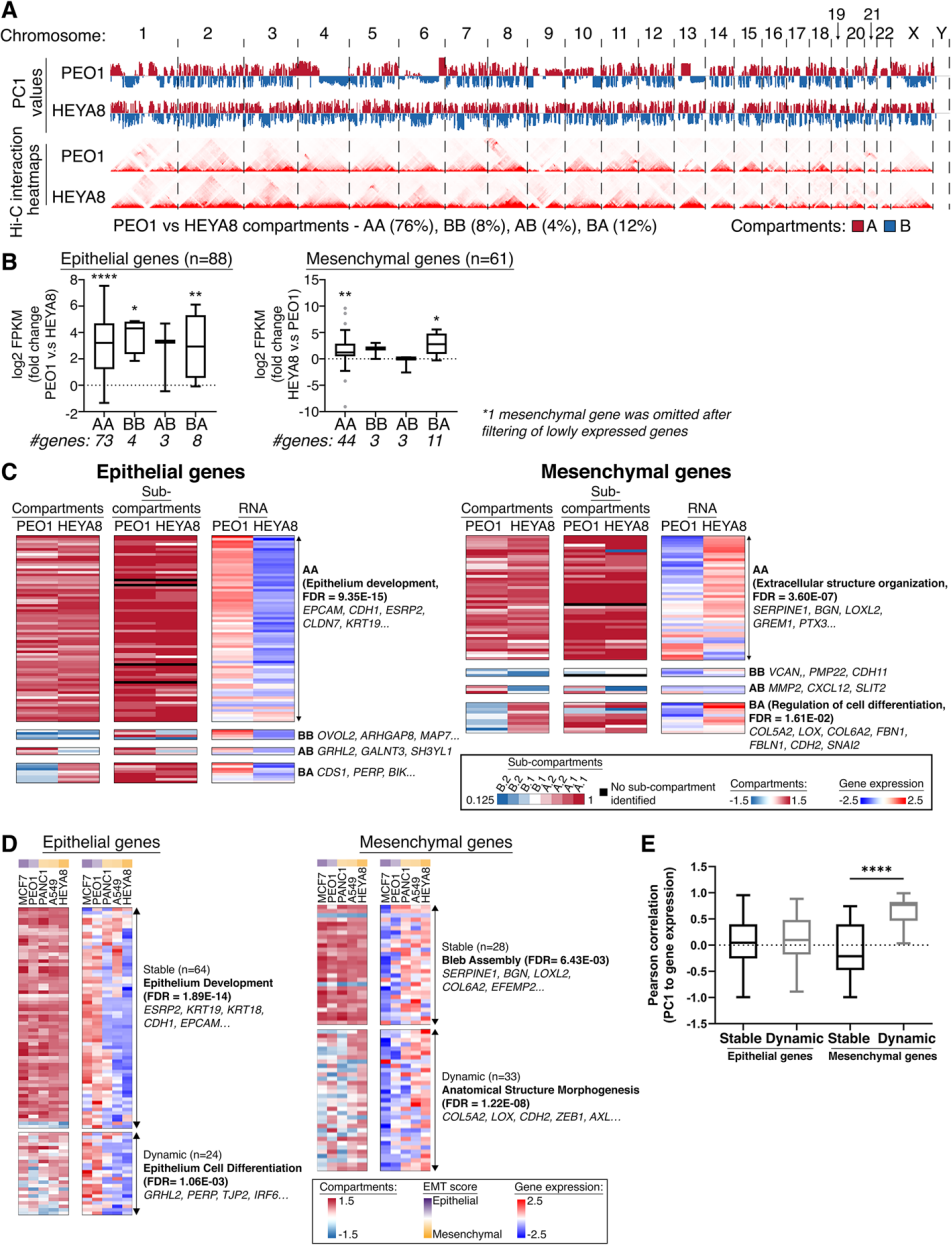
**a** Top panels: genome-wide (*x*-axis) compartments A (red) and B (blue) in PEO1 and HEYA8 based on the PC1 values (*y*-axis); bottom panels: genome-wide Hi-C interaction heatmaps in PEO1 and HEYA8. **b** Box plots showing fold change of log2 FPKM expression (*y*-axis) of 88 epithelial (left) and 61 mesenchymal (right) genes categorized by the compartment changes (*x*-axis; AA, BB, AB, BA) between PEO1 versus (v.s) HEYA8. *P* values were obtained by Wilcoxon test, *p<0.05, **p<0.01 and ****p<0.0001.  **c** Heatmaps of the 2 major compartments A (red) and B (blue), sub-compartments A.1.1 (dark red), A.1.2 (red), A.2.1 (light red), A.2.2 (pale red), B.1.1 (white), B.1.2 (light blue), B.2.1 (blue), and B.2.2 (dark blue), and RNA expression in the scale of *Z*-scores of epithelial and mesenchymal genes in PEO1 and HEYA8. The GO terms for the compartment switches (AA, BB, AB, and BB) are shown at the right of the RNA expression heatmap, where available. **d** Heatmap of the 2 major compartments A (red) and B (blue) and RNA expression in the scale of *Z*-scores of epithelial and mesenchymal genes in MCF7 (dark purple), PEO1 (light purple), PANC1 (pale yellow), A549 (light yellow), and HEYA8 (dark yellow) of different EMT scores along the spectrum. The GO terms for the stable or dynamic compartment changes are shown at the right of the RNA expression heatmap, where available. **e** Box plot of Pearson correlation (*y*-axis) between the PC1 values of the major compartments and expression of epithelial and mesenchymal genes based on the stable or dynamic compartment changes (*x*-axis)

To further decipher the regulation of EMT in cancer, the 3D genome architecture of the refined EMT signature genes were explored. High-throughput chromatin conformation capture (Hi-C) technique was performed in two ovarian cancer cell lines PEO1 (EMT score −0.328) and HEYA8 (EMT score 0.643) that represented the E and M states. The Hi-C libraries were sequenced at a high depth to allow better resolution of the 3D genome organization in these cell lines (Additional file 2: Fig. S2A – H and Additional file 3: Table S1). The genome-wide chromatin interactions can be depicted as patterns of interactions where regions of the same type tend to have higher interactions frequencies than those regions of a different type. These regions usually occur on a chromosomal scale and are referred to as A and B compartments [29, 42, 43]. Genome-wide differences in the A and B compartments between PEO1 and HEYA8 could be observed on a chromosomal-wide scale (Fig. 2A). The Hi-C data of two ovarian cancer cell lines revealed that, overall, the genes in the A compartment had higher expression compared to those in the B compartment (Additional file 2: Fig. S3A), reflecting the nature of the A compartment being the active euchromatic compartment. The changes in the compartments between PEO1 and HEYA8 can be categorized mainly into 3 types: (i) “Stable”—either “active” in both PEO1 and HEYA8 (AA) or “inactive” in both PEO1 and HEYA8 (BB); (ii) “AB”—compartment switching from “active” in PEO1 to “inactive” in HEYA8; (iii) “BA”—compartment switching from “inactive” in PEO1 to “active” in HEYA8 (Fig. 2A). Between the two cell lines, majority of the genes were in the stable active compartments (PEO1 versus HEYA8 compartments: AA 76%, BB 8%), while only a small fraction of the genes was in the dynamic compartments (PEO1 versus HEYA8 compartments: AB 4% and BA 12%) (Fig. 2A). This suggested that there was only a slight degree of plasticity in the compartments and the 3D genome changes at the hierarchical order and the compartments were relatively stable between the two states. To explore whether the compartmentalization of the 3D genome could define the differences between the E and M states via controlling the transcription of genes residing in each compartment, the concordance of the gene expression levels to the nature of the residing compartment was examined with the assumption that the gene expression level in the active compartment would be higher and vice versa. At the genome-wide scale, there was indeed differential gene expression in the dynamic compartments in both AB and BA (*p* < 0.001, Additional file 2: Fig. S3B). However, only 33% and 34% of the genes in BA and AB displayed concordance between gene expression and compartment changes (Additional file 2: Fig. S3B). Gene ontology (GO) of these concordant genes showed that the genes that switched from the inactive to active compartments between PEO1 and HEYA8 (BA) were enriched in cellular metabolic processes (FDR= 2.07 × 10^−13^, Additional file 2: Fig. S3B), while genes in AB did not reveal specific GO.

Zooming into the EMT genes, most of the E and M genes remained in the stable AA and BB compartments (Epithelial_AA+BB_ = 77, 87.5%; Mesenchymal_AA+BB_ = 47, 77%; Fig. 2B. Additional file 4: Table S2A and 2C), while a smaller of proportion of the EMT genes exhibited changes in compartment status between the two cell lines (Epithelial_AB+BA_ = 11, 12.5%; Mesenchymal_AB+BA_ = 14, 23%; Fig. 2C. Additional file 4: Table S2A). In terms of the concordance of gene expression levels, EMT genes showed differential expression specific to the EMT state regardless of the stable (AA/BB) and dynamic (AB/BA) compartments (Fig. 2B). The E genes were all more highly expressed in PEO1 across the compartment categories (Fig. 2C). The M genes in AA were highly expressed in HEYA8 (*p* < 0.01, Fig. 2B). Notably, the M genes in the dynamic BA compartments showed significant upregulation in the M state (*p* < 0.05, Fig. 2B). GO analysis (Fig. 2C) further revealed that the E genes in AA were enriched in epithelium development (FDR = 9.35 × 10^−15^, Additional file 4: Table S2B) including *EPCAM*, *CDH1*, *ESRP2*, *CLDN7*, and *KRT19*. No specific GO was annotated in other categories. Of note, the M genes in AA were enriched in processes of the extracellular matrix organization (FDR= 3.60 × 10^−7^, Additional file 4: Table S2D) including *SERPINE1*, *BGN*, *LOXL2*, *GREM1*, and *PTX3*. Interestingly, the M genes in BA were enriched in genes involved in the regulation of cell differentiation (FDR = 1.61 × 10^−2^, Additional file 4: Table S2E) including *COL5A2*, *LOX*, *COL6A2*, *FBN1*, *FBLN1*, *CDH2*, and *SNAI2*, indicating that this subset of M genes may require genome reorganization at the higher-order hierarchy during EMT. In both PEO1 and HEYA8, compartment A showed correlation to active histone marks (H3K27ac, H3K4me1, and H3K4me3), while the repressive histone mark H3K27me3 was weakly correlated to both compartments A and B, and H3K9me3 was correlated to compartment B in HEYA8 (Additional file 2: Fig. S3C).

### Sub-compartments of the chromatin did not have a deterministic role in regulating the EMT genes in the AA compartments

As majority of E genes in HEYA8 remained in the active compartment despite being repressed transcriptionally, we questioned if a refined sub-compartmentalization of the chromatin [44] would better segregate the EMT genes to identify intermediate sub-compartments enriched in H3K27me3 in HEYA8. The E genes in AA were mostly assigned to active sub-compartments A.1.1 and A.1.2 in both PEO1 and HEYA8 (Fig. 2C, Additional file 4: Table S2H). This suggested that the repressed expression of these E genes in AA in HEYA8 indeed did not involve significant compartmental switch. Interestingly, E genes in BB and BA were re-assigned to active sub-compartments A.1.1, A.1.2, A.2.1, and A.2.2 in PEO1 without significant change in sub-compartments in HEYA8 (Fig. 2C. Additional file 4: Table S2F and S2H). This suggested that these E genes could be regulated further by sub-compartmentalization of the chromatin with the sub-compartments being the fundamental units of the chromatin organization. Similarly, the M genes in AA were assigned to the active sub-compartments A.1.1 and A.1.2 in both PEO1 and HEYA8 (Fig. 2C. Additional file 4: Table S2G). This confirmed that the repressed expression of these M genes in AA in PEO1 also did not involve significant compartmental switch. In addition, the M genes in BB were assigned to sub-compartments B.1.1 and B.1.2, whereas the M genes in BA with a corresponding increase in expression in HEYA8 were re-assigned to sub-compartments A.1.1, A.1.2, A.2.1, A.2.2, and B.1.2 in PEO1 (Fig. 2C. Additional file 4: Table S2G and S2H), signifying that these M genes would also be regulated further at units within the sub-compartments. At a broader scale, when segregating the chromatin into two major compartments, the rearrangement of the M genes from an inactive to active compartment, or vice versa, may pose an epigenetic barrier on the activation of M genes during EMT. But at a finer scale of the sub-compartmentalization, it is evident that regulation of the EMT genes occurs in a more intrinsic manner, where smaller units within the sub-compartments may better explain the regulation of the genes in a 3D context. In summary, in this ovarian cancer cell line model, the compartmental changes did not occur in most of the E genes while a subset of M genes required for cell differentiation did show association to the compartment changes between EM states at a broader scale of chromatin compartments.

### Mesenchymal genes involved in morphogenesis showed compartmental changes along the EMT spectrum

We extended the analysis of the EMT genes in their major chromatin compartments A and B, to a panel of 5 cancer cell lines along the EMT spectrum with annotated EMT scores (Fig. S4A), which had 3D genome (Hi-C) and epigenetic (histones ChIP-seq) information available. The expression levels of E and M genes did show good correlation with the EMT spectrum (Additional file 2: Fig. S4B – C).

Evidently, majority of the E genes remained stable in the AA/BB compartments (*n* = 64, 72.7%, Additional file 5: Table S3A) across the 5 cancer cell lines (Fig. 2D). These genes were involved in epithelium development including *ESRP2*, *KRT19*, *KRT18*, *CDH1*, and *EPCAM* (FDR_stable_ = 1.89 × 10^−14^, Fig. 2D. Additional file 5: Table S3B). Twenty-four (27.3%) E genes showed dynamic compartment changes across 5 cancer cell lines. The E genes that showed dynamic compartment changes across the cell lines were involved in epithelium differentiation including *GRHL2*, *PERP*, *TJP2*, and *IRF6* (FDR_dynamic_ = 1.06 × 10^−3^, Fig. 2D. Additional file 5: Table S3C). Overall, the E genes were still being repressed in the M cell lines, regardless of the compartment status (Fig. 2D). Interestingly, for the M genes, we observed a higher degree of plasticity in the compartment changes across the cancer cell lines (Mesenchymal_stable_ = 28, 45.9%; Mesenchymal_dynamic A/B_ = 33, 54.1%; Fig. 2D. Additional file 6: Table S4A). The M genes in the stable compartments were involved in the process of bleb assembly, including genes such as *SERPINE1, BGN, LOXL2, COL6A2*, and *EFEMP2* (FDR_stable_ = 6.43 × 10^−03^; Fig. 2D. Additional file 6: Table S4B). In addition, the M genes that exhibited dynamic compartment changes across the cancer cell lines (Additional file 6: Table S4A) were enriched in the biological process related to anatomical structure morphogenesis, including *COL5A2*, *LOX*, *CDH2*, *ZEB1*, and *AXL* (FDR_dynamic_ = 1.22 × 10^−8^; Fig. 2D. Additional file 6: Table S4C). This highlighted that the M genes involved in cell morphology changes during EMT would require to be regulated by compartmental changes. The expression of M genes in the dynamic compartments were significantly correlated to the compartment changes across the cell lines, while the E genes did not show any correlation between the compartment changes and expression (*p* = 5.67 × 10^−4^; Fig. 2E). Taken together, our analysis suggested that the M genes involved in morphogenesis are likely to be regulated at the higher hierarchical order of the active/inactive compartments at the genome level along the EMT spectrum.

### Epithelial genes in the eTADs were regulated at the histone levels regardless of the TAD boundary changes

**Fig. 3:**
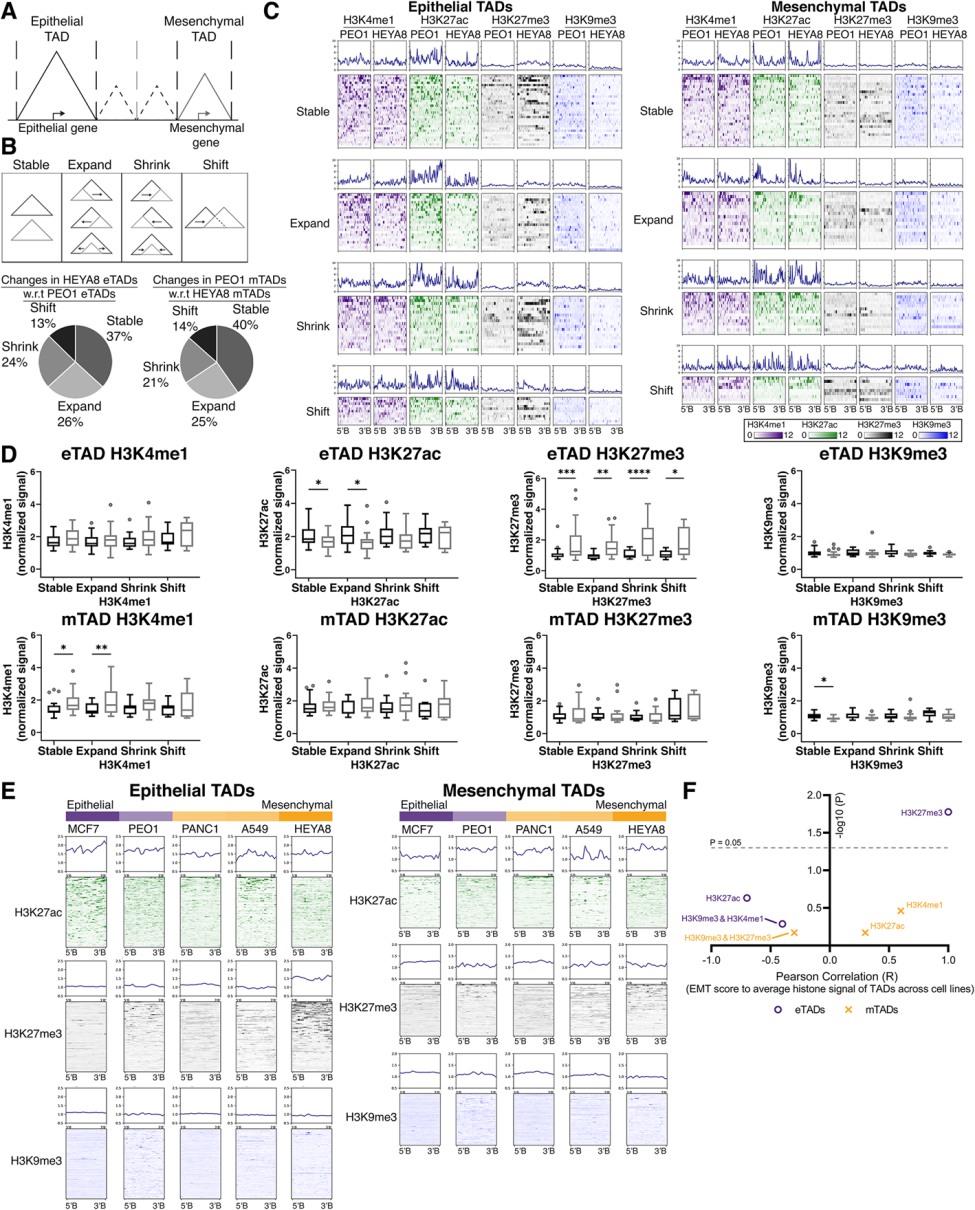
**a** Definition of epithelial and mesenchymal TADs. **b** Top panel: Illustration of changes in EMT TADs between PEO1 (epithelial state) and HEYA8 (mesenchymal state), described by 4 categories: (i) Stable, (ii) Expand, (iii) Shrink, and (iv) Shift. The dashed lines represent the TAD boundaries that were identified in the cancer cell line, which was used as a reference of comparison. Bottom panel: Pie charts annotating the percentages of changes in HEYA8 eTADs with respect to PEO1 eTADs (left) and changes in PEO1 mTADs with respect to HEYA8 mTADs (right). **c** Distribution profile plots and heatmaps of the histones marks between PEO1 and HEYA8 in epithelial (left) and mesenchymal (right) TADs, split into the categories of TAD changes: (i) Stable, (ii) Expand, (iii) Shrink, and (iv) Shift. H3K4me1, H3K27ac, H3K27me3, and H3K9me3 are represented by indigo, green, black, and blue respectively. **d** Boxplots of normalized histones signal (*y*-axis; H3K4me1, H3K27ac, H3K27me3, and H3K9me3) in eTADs (top row) and mTADs (bottom row) of PEO1 (black) and HEYA8 (grey). The histones signals were split into the categories of TAD changes within each histone marks: (i) Stable, (ii) Expand, (iii) Shrink, and (iv) Shift. Student’s *t* test was carried out between PEO1 and HEYA8 for the respective histone marks, *p<0.05 **p<0.01, ***p<0.001 and ****p<0.0001. **e** Distribution profile plots and heatmaps of histone marks (H3K27ac—green, H3K27me3—black, and H3K9me3—blue) in eTADs (left) and mTADs (right) of cancer cell lines representing different states of the EMT spectrum. The relative epithelial and/or mesenchymal states of the cancer cell lines are shown as a color bar on top of the heatmaps (epithelial state—purple, mesenchymal state—orange). **f** Scatter plot showing Pearson correlation (R, *x*-axis) of EMT score to average histone signal in eTADs (purple circle) and mTADs (orange cross) across the cancer cell lines

To understand how the EMT genes could be further regulated, insights were sought by digging into the next higher order of chromatin structure occurring at the sub-chromosomal scale, the topological associating domains (TADs) [31, 32]. The TAD which encompasses an E gene is termed as an epithelial TAD (eTAD); likewise, the TAD with an M gene is defined as mesenchymal TAD (mTAD) (Fig. 3A). At the TAD level, we questioned if the boundary changes in the TADs between the PEO1 (E) and HEYA8 (M) states had a role in the modulation of the EMT genes. The changes in the TAD boundaries can be briefly categorized into (i) TADs with unchanged boundaries—“Stable”; (ii) TADs with shifted boundaries—“Expand,” “Shrink,” and “Shift.” A similar proportion of E and M TADs retain their TAD boundaries between the two EMT states (37% in eTADs; 40% in mTADs; Fig. 3B). Similar proportion of the eTADs/mTADs which displayed boundary changes between the E and M states were also observed (“Expand”—26% eTADs, 25% mTADs; “Shrink”—24% eTADs, 21% mTADs; “Shift”—13% eTADs, 14% mTADs; Fig. 3B). We then questioned if these changes in the EMT TADs between the two states had any impact on the epigenomic landscape in the TADs. The eTADs in HEYA8 had significantly higher enrichment of the repressive H3K27me3 mark while the eTADs in PEO1 had higher enrichment of active H3K27ac chromatin mark, regardless of the TAD boundary changes (Fig. 3C, D). In the mTADs, the changes in the chromatin marks between the E and M states were subtle, regardless of the TAD boundary changes, since there was no enrichment of the repressive H3K27me3 mark nor the decrease of the active marks in the mTADs of the E state (Fig. 3C, D). Our data suggested that the E genes in the TADs were repressed in the M state at the chromatin level for histone modifications, while the subtle changes in the histone marks in the mTADs pointing to a different trajectory path of regulation for the M genes. We then questioned if DNA methylation had a role in the regulation of the genes in the EMT TADs. Firstly, the E genes were methylated in the M state as compared to the E state (*p* < 0.0001. Additional file 2: Fig. S5A), while the M genes did not show any significant difference between the E and M states in methylation levels, which corroborated with a previous study [23]. The methylation levels of the genes in mTADs were higher than the eTADs in the E state (*p*_PEO1-eTAD vs PEO1-mTAD_ < 0.05. Additional file 2: Fig. S5B). However, the methylation levels of the genes in the EMT TADs were generally higher in the M state (*p*_PEO1-eTAD vs HEYA8-eTAD_ < 0.0001, *p*_PEO1-mTAD vs HEYA8-mTAD_ < 0.0001. Additional file 2: Fig. S5B).

### H3K27me3 in eTADs contributed to the regulation of the epithelial genes in M state of the EMT spectrum

In the EMT spectrum model across the 5 cancer cell lines, we observed that the M-like cancer cell lines had a higher enrichment of H3K27me3 compared to E-like cancer cell lines with a positive correlation to the EMT score (Fig. 3E). However, this was not observed for H3K9me3 in the eTADs (Fig. 3E). The repressive H3K27me3 mark in the eTADs and at the E genes correlated positively with the EMT scores (Fig. 3F, Pearson correlation_eTADs_ = 0.933, *p*_eTADs_ = 0.021. Additional file 2: Fig. S4D, Pearson correlation_Egenes_ = 0.938, *p*_Egenes_ = 0.018. Additional file 7: Table S5). There was also a strong negative correlation between the H3K27ac signal in the eTADs and at the E genes, to the EMT score of the cancer cell lines, albeit not being significant in the eTADs (Fig. 3F, Pearson correlation_eTADs_ = −0.691, *p*_eTADs_ = 0.197. Fig. S4D, Pearson correlation_Egenes_ = −0.917, *p*_Egenes_ = 0.029. Additional file 7: Table S5). This suggested that the eTADs adopted a more repressed chromatin state in the M-like cancer cell lines, with the enrichment of H3K27me3. On the other hand, the mTADs in the E-like cancer cell lines did not exhibit higher enrichment of H3K27me3, which was in concordant with the ovarian cancer model. In the mTADs and corresponding M genes, there was no correlation between the repressive H3K27me3 mark and the EMT score (Fig. 3F, Pearson correlation_mTADs_ = −0. 308, *p*_mTADs_ = 0.614. Fig. S4D, Pearson correlation_Mgenes_ = −0.306, *p*_Mgenes_ = 0.617. Additional file 7: Table S5) There was also no significant correlation between other histone marks (H3K27ac, H3K4me1, and H3K9me3) and the EMT score of the cancer cell lines for the mTADs (Fig. 3F. Additional file 2: Fig. S4D. Additional file 7: Table S5). Taken together, the repressive histone modification marked by H3K27me3 contributed to the regulation of the E genes where both the eTADs and the E genes adopted a repressed chromatin mark in the M state, leading to a reduced expression of the E genes. Whereas in the case of the mTADs and M genes, the reduced expression of the M genes in the E state did not equate to a repressed chromatin state akin to the eTADs.

### Changes in H3K27me3 in the mTADs and M genes were less associated to the EMT states

**Fig. 4:**
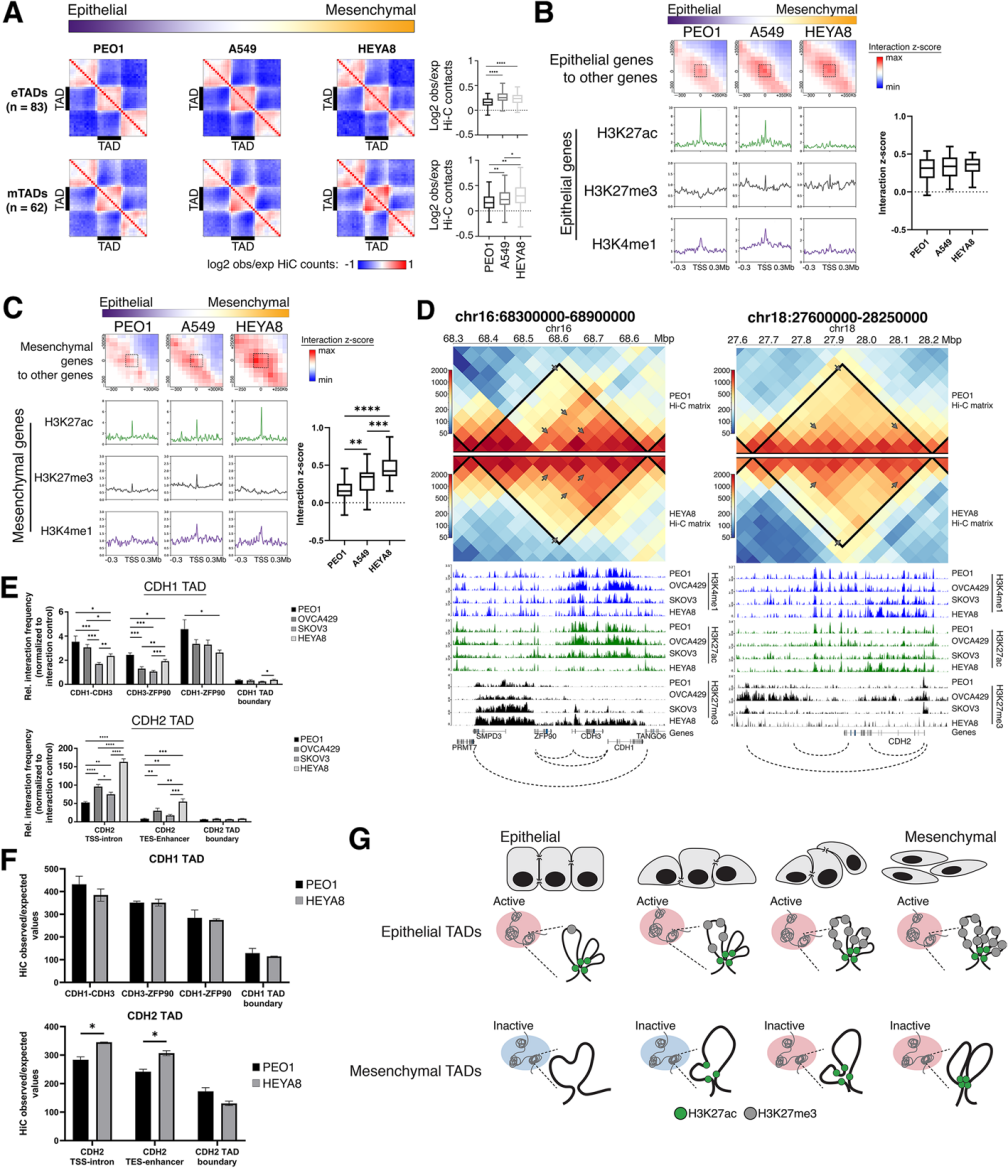
**a** Aggregate TAD plot showing normalized observed/expected Hi-C counts in the eTADs (top row) and mTADs (bottom row) of cancer cell lines—PEO1 (purple), A549 (white), and HEYA8 (orange)—arranged in the order of increasing EMT score (from epithelial to mesenchymal state). Boxplot representations of the observed/expected Hi-C counts (*y*-axis) within the eTADs or mTADs of the cancer cell lines (*x*-axis) are depicted on the right of the aggregate TAD plots. Student’s *t* test was used for the statistical analysis, ****p < 0.0001. **b** Aggregate Hi-C matrices showing the interaction *z*-scores (red: high interaction *z*-score, blue: low interaction *z*-score) at pairwise genomic regions, between TSS of epithelial genes to the other genes within the eTADs of cancer cell lines—PEO1 (purple), A549 (white), and HEYA8 (orange)—arranged in the order of increasing EMT score (from epithelial to mesenchymal state). Distribution profile plots of the histone marks (H3K27ac—green, H3K27me3—black, and H3K4me1—indigo) at the TSS of epithelial genes were shown below the respective aggregate Hi-C matrices. Boxplot representation of interaction *z*-scores, as highlighted by the dashed boxes in the aggregate matrices, (*y*-axis) at the TSS of epithelial genes across the cancer cell lines (*x*-axis) of different EMT scores along the EMT spectrum. Student’s *t* test was used for the statistical analysis. **c** Aggregate Hi-C matrices showing the interaction *z*-scores (red: high interaction *z*-score, blue: low interaction *z*-score) at pairwise genomic regions, between TSS of mesenchymal genes to the other genes within the mTADs of cancer cell lines—PEO1 (purple), A549 (white), and HEYA8 (orange)—arranged in the order of increasing EMT score (from epithelial to mesenchymal state). Distribution profile plots of the histone marks (H3K27ac—green, H3K27me3—black, and H3K4me1—indigo) at the TSS of mesenchymal genes were shown below the respective aggregate Hi-C matrices. Boxplot representation of interaction *z*-scores, as highlighted by the dashed boxes in the aggregate matrices, (*y*-axis) at the TSS of mesenchymal genes across the cancer cell lines (*x*-axis) of different EMT scores along the EMT spectrum. Student’s *t* test was used for the statistical analysis, **p< 0.01, ***p<0.001 and ****p<0.0001. **d** Triangular Hi-C matrix showing the normalized Hi-C counts at *CDH1* and *CDH2* TADs (chr16:68,300,000–68,900,000 and chr18:27,600,000–28,250,000, respectively) in PEO1 (top Hi-C matrix) and HEYA8 (bottom inverted Hi-C matrix). The TADs at *CDH1* and *CDH2* loci are represented by the solid black lines drawn on the Hi-C matrix. Histone marks in the 4 ovarian cancer cell lines (PEO1, OVCA429, SKOV3, and HEYA8) are shown below the Hi-C matrices. H3K4me1, H3K27ac, and H3K27me3 are represented by indigo, green, and black respectively. The gene track (hg38) is shown below the histone marks tracks. The dotted arcs, below the gene track, represent the interacting loci that were used in assessing the interaction frequency by 3C-qPCR. The interacting loci were also annotated in the triangular Hi-C matrices by the grey arrows. **e** Bar chart representation of 3C-qPCR interaction frequencies (*y*-axis) between interacting loci (*x*-axis) annotated in **d**, in 4 ovarian cancer cell lines (PEO1, OVCA429, SKOV3, and HEYA8). The interacting loci assessed by 3C-qPCR at the *CDH1* TAD are “CDH1-CDH3” (*CDH1* TSS to *CDH3* TSS), “CDH3-ZFP90” (*CDH3* TSS to *ZFP90* TSS), “CDH1- ZFP90” (*CDH1* TSS to *ZFP90* TSS), and “CDH1 TAD boundary” (*CDH1* TAD 5′boundary to 3′boundary). The interacting loci assessed by 3C-qPCR at the *CDH2* TAD are “CDH2 TSS-intron” (*CDH2* TSS to *CDH2* intronic enhancer), “CDH2 TES-Enhancer” (*CDH2* TES to *CDH2* upstream enhancer), and “CDH2 TAD boundary” (*CDH2* TAD 5′boundary to 3′boundary). Interaction frequencies were normalized to loading control and Student’s *t* tests were used for statistical analyses. *p<0.05, **p<0.01, ***p<0.001, and ****p<0.0001. **f** Top bar plot depicting Hi-C observed/expected values (*y*-axis) between interacting loci (*x*-axis) in *CDH1* TAD, in PEO1 (black) and HEYA8 (grey). The interacting loci at the *CDH1* TAD are as follows: “CDH1-CDH3” (*CDH1* TSS to *CDH3* TSS), “CDH3-ZFP90” (*CDH3* TSS to *ZFP90* TSS), “CDH1- ZFP90” (*CDH1* TSS to *ZFP90* TSS), and “CDH1 TAD boundary” (*CDH1* TAD 5′boundary to 3′boundary). Bottom bar plot depicting Hi-C observed/expected values (*y*-axis) between interacting loci (*x*-axis) in *CDH2* TAD, in PEO1 (black) and HEYA8 (grey). The interacting loci at the *CDH2* TAD are as follows: “CDH2 TSS-intron” (*CDH2* TSS to *CDH2* intronic enhancer), “CDH2 TES-Enhancer” (*CDH2* TES to *CDH2* upstream enhancer), and “CDH2 TAD boundary” (*CDH2* TAD 5′boundary to 3′boundary). Student’s *t* tests were used for statistical analyses, *p<0.05. **g** Illustration of 3D genome structure and epigenetic landscape changes in the epithelial and mesenchymal TADs during different EMT states along the EMT spectrum. H3K27ac and H3K27me3 are represented by green and grey respectively. The active compartment A and inactive compartment B are represented by red and blue respectively

Prior studies in the 3D genome have shown that the most fluid variable of the chromatin conformation is the promoter-enhancer interactome, exhibiting significant degree of cell-type specificity [45–49]. We hypothesized that the underlying mechanism in the regulation of the M genes could be influenced by the interactome differences within the TADs. Therefore, we looked at the normalized contact probabilities within the EMT TADs in three cell lines, PEO1, A549, and HEYA8, representing the E, EM, and M states. We observed that the eTADs in the EM (A549) and M (HEYA8) cell lines had slightly higher interaction frequency within the eTADs (*p*_PEO1-A549_ < 0.0001, *p*_PEO1-HEYA8_ < 0.0001; Fig. 4A). As we know that eTADs showed a decrease in H3K27ac and enrichment in H3K27me3 mark in the M state (Fig. 3E, F), the increase in the contact frequency within the eTADs in the EM and M state could be due to the compaction of the chromatin as we observed a significantly lower accessibility in the eTADs in HEYA8 as compared to PEO1 (Additional file 2: Fig. S6A). On the other hand, the mTADs in all three cell lines exhibited strong boundary-boundary interactions (Fig. 4A). There was higher contact frequency in the mTADs of HEYA8 as compared to PEO1 and A549, as well as higher contact frequency in A549 when compared to PEO1 (*p*_HEYA8-PEO1_ < 0.01, *p*_HEYA8-A549_*<* 0.05, *p*_A549-PEO1_*<* 0.01 Fig. 4A). Knowing that the mTADs did not show significant enrichment of repressive histone marks (Fig. 3E, F), the frequent contact probabilities within the mTADs of the M state might suggest increased loop formation, since the mTADs in the E-like and the M-like cell lines did not differ in terms of accessibility (Additional file 2: Fig. S6A). In addition, we observed similar levels of CTCF and cohesin complex proteins across the E-like and M-like cell lines (Additional file 2: Fig. S6B) thus implying that the boundaries of the mTADs do not play a significant role towards the changes in contact probabilities within the TADs.

### Local chromatin interaction with the regulatory elements in TADs contributed to the regulation of EMT genes

We continued to examine the spatial proximity and the chromatin loop interactions between EMT genes and other non-EMT genes, or EMT genes and its regulatory elements, within the same TAD. To approach this, we performed an aggregate analysis between the transcription start site (TSS) of EMT genes and other non-EMT genes within the same TADs. The interaction scores of the E genes coming into spatial proximity of the other genes within the same TADs were similar along the EMT spectrum, showing no significant differences between the E-like and M-like cell lines (Fig. 4B). The histone profiles around these spatial proximity sites in the eTADs showed the trend as the active H3K27ac mark decreased and the H3K27me3 mark increased from E to EM, to M (Fig. 4B). In the mTADs, non-EMT genes were also in proximity with the M genes. However, in PEO1, the genes contacted less frequently with each other compared to the M-like cell lines, A549 and HEYA8 (Fig. 4C). Given the subtle changes in the histone marks within the mTADs between the E and M state, the aggregate contact frequency suggested that loop interactions in the mTADs could play a bigger role in the regulation of the M genes.

3C-qPCR was used to validate the loop interactions of the EMT genes within their respective TADs to assess if the increasing chromatin interaction frequency along the EMT spectrum, at the M genes to non-EMT genes in the same TAD was indeed occurring. Four ovarian cancer cell lines (PEO1, OVCA429, SKOV3, and HEYA8) representing the E, Intermediate E (IE), Intermediate M (IM), and M states along the EMT spectrum [23] (Additional file 2: Fig. S4A) were examined at the two classical E and M genes of the cadherin family – E-Cadherin (*CDH1*) and N-Cadherin (*CDH2*). At the *CDH1* TAD, the M-like cell lines (SKOV3 and HEYA8) adopted a more repressed chromatin state while the E-like cell lines (PEO1 and OVCA429) had an open chromatin state according to the histone peaks (Fig. 4D). *CDH1* interacted more frequently to other genes (*CDH3* and *ZFP90*) within the same TAD in the E-like cell lines (PEO1 and OVCA429) (Fig. 4D, E). In the 2 EMT states (PEO1 and OVCA429 – E, IE), the expression of both *CDH1* and *CDH3* were similar, corresponding to higher *CDH1-CDH3* interaction frequencies (Fig. 4E. Additional file 2: Fig. S6D). Notably, the decrease in *CDH1-CDH3* contact strength also corresponded to the decreasing expressions of both *CDH1* and *CDH3* (Fig. 4E. Additional file 2: Fig. S6D). We observed that in SKOV3 (IM state), which had the lowest *CDH1-CDH3* interaction frequency among the 4 cell lines, also showed low *CDH1* and *CDH3* expression (Fig. 4E. Additional file 2: Fig. S6D). Interestingly, HEYA8 (M state) which had the lowest *CDH1* and *CDH3* expression among the four showed slight increase of *CDH1*-*CDH3* interaction frequency compared to SKOV3 (Fig. 4E. Additional file 2: Fig. S6D). However, we did not see the same phenomena between the co-expression of genes and the gene-gene contact strength in *CDH3-ZPF90* or *CDH1-ZFP90* interactions (Fig. 4E. Additional file 2: Fig. S6D). We speculated that there might be a sub-TAD present harboring both *CDH1* and *CDH3*.

In contrast, at the *CDH2* TAD, the M-like cell lines were enriched in active histone marks (H3K27ac and H3K4me1) while the E-like cell lines showed a slight increase in H3K27me3 binding across the TAD (Fig. 4D). 3C-qPCR showed that *CDH2* interacted strongly with its intronic enhancer in HEYA8 while this interaction became weaker as the H3K4me1 and H3K27ac marks were depleted in the less M-like cells (PEO1, OVCA429, and SKOV3) (Fig. 4E). There was up to 2-fold increase in interaction frequency in *CDH2* TSS to the intronic enhancer between HEYA8 and PEO1 (Fig. 4E). Likewise, this pattern of increased interaction frequency in the M state was also observed between the transcription end site (TES) of *CDH2* and an upstream enhancer in the *CDH2* TAD (Fig. 4D, E). In both *CDH1* and *CDH2* TADs, the TAD boundaries displayed similar interaction frequency between all the EMT states (Fig. 4E). In agreement with the 3C-qPCR observations, Hi-C data at the *CDH2* TAD showed higher interaction frequencies in HEYA8 at the interacting loci of *CDH2* TSS-intron, *CDH2* TES-enhancer (Fig. 4F). Albeit not being significant, we did observe higher interaction between *CDH1* and *CDH3* in the Hi-C data of PEO1 as compared to HEYA8 (Fig. 4F). In summary, the eTADs adopted an open chromatin state in the E state compared to the M state with 3C-qPCR showing increased interaction between selected E genes such as *CDH1* and *CDH3* with non-EMT genes in the same domain. In the mTADs, both 3C-qPCR and Hi-C suggested that the changes in the local chromatin conformation with the M genes such as *CDH2* could play a bigger role in the transcriptional activity of these genes (Fig. 4G).

### Regulation of *SNAI2* via concerted coordination among transcription factor binding, enhancer looping, and the chromatin state

**Fig. 5:**
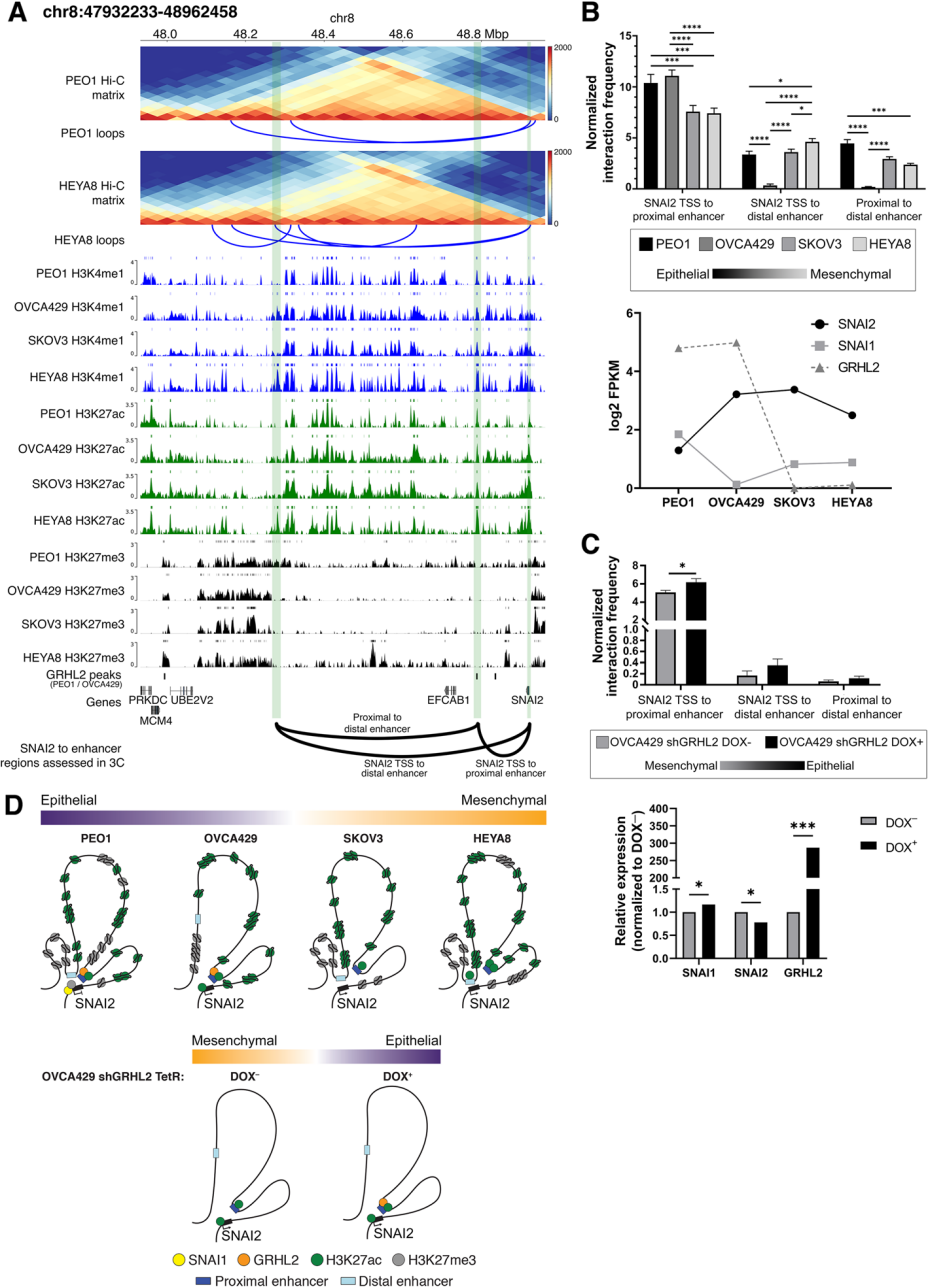
**a** Triangular Hi-C matrix depicting the Hi-C counts at *SNAI2* TAD in PEO1 (top Hi-C matrix) and HEYA8 (bottom Hi-C matrix). The loops (blue solid lines) identified from the Hi-C data are shown below their respective Hi-C matrices. Histone marks in the 4 ovarian cancer cell lines are shown below the Hi-C matrices. H3K4me1, H3K27ac, and H3K27me3 are represented by indigo, green, and black respectively. *GRHL2* binding regions (*GRHL2* peaks) at *SNAI2* TAD are shown below the histone marks tracks. The gene track (hg38) is shown below the *GRHL2* peaks. The green stripes correspond to the regions at the *SNAI2* TAD: *SNAI2* gene, proximal enhancer, and distal enhancer. The solid arcs represent the interacting loci that were used in assessing interaction frequency by 3C-qPCR, “SNAI2 TSS to proximal enhancer,” “SNAI2 TSS to distal enhancer,” and “proximal to distal enhancer.” **b** Bar chart representation of 3C-qPCR normalized interaction frequencies between interacting loci as annotated in **a**. The normalized interaction frequency (*y*-axis) at the interacting loci (axis) were shown in 4 ovarian cancer cell lines (PEO1—black, OVCA429—dark grey, SKOV3—grey, and HEYA8—light grey), representing different EMT states across the EMT spectrum (epithelial—black, mesenchymal—light grey). RNA-seq log2(FPKM) expression of *SNAI1* (solid grey line with square symbol), *SNAI2* (solid black line with circle symbol), and *GRHL2* (dotted dark grey line with triangle symbol) is shown below the bar chart for the respective cell lines. Student’s *t* tests were used for statistical analyses. *p<0.05, ***p<0.001, and ****p<0.0001. **c** Bar chart representation of 3C-qPCR normalized interaction frequencies between interacting loci as annotated in **a**. The normalized interaction frequency (*y*-axis) at the interacting loci (*x*-axis) were shown in OVCA429 shGRHL2 DOX^–^ (dark grey; mesenchymal state) and OVCA429 shGRHL2 DOX^+^ (black; epithelial state). 3C-qPCR showing normalized interaction frequencies between SNAI2 TSS and its proximal/distal enhancers in OVCA429 shGRHL2 Tet-inducible cell line (epithelial state—black, mesenchymal state—grey). Bar chart representation of log2 fold change (*y*-axis) of *SNAI1*, *SNAI2*, and *GRHL2* (*x*-axis) between OVCA429 shGRHL2 DOX^–^ (dark grey) and OVCA429 shGRHL2 DOX^+^ (black) is shown below the 3C-qPCR bar chart. The log2 fold change expression (*y*-axis) was determined by RT-qPCR. Student’s *t* tests were used for statistical analyses. *p<0.05 and ***p<0.001. **d** Illustration of local chromatin structure at *SNAI2* locus in the 4 ovarian cancer cell lines (PEO1—purple, OVCA429—light purple, SKOV3—light orange, and HEYA8—orange) representing different EMT states along the spectrum, as well as the changes in chromatin conformation at *SNAI2* locus as the cell transits from a mesenchymal (OVCA429 shGRHL2 DOX^–^, orange) to epithelial (OVCA429 shGRHL2 DOX^+^, purple) state. The representations of the proteins, histones, and enhancer at the *SNAI2* locus are as indicated: SNAI1—yellow circle, GRHL2—orange circle, H3K27ac—green circle, H3K27me3—grey circle, proximal enhancer—dark blue rectangle, and distal enhancer—light blue rectangle. The *SNAI2* gene is represented by a black rectangle and the *SNAI2* TSS is indicated by the black arrow appended at its side

Among the EMT-TFs, the regulation of *SNAI2* is less understood [50, 51] compared to its paralog *SNAI1* [52, 53]. We thus continued to explore the chromatin looping within the *SNAI2* TAD. From the Hi-C data, we observed 2 loops arising from the *SNAI2* promoter, of which one of the loops shows an interaction between *SNAI2* TSS and a distal enhancer (Fig. 5A). In addition, there is a proximal enhancer to *SNAI2* that consists of the binding site of GRHL2 (Fig. 5A), a gatekeeper of the E state [22]. Although no loops were detected between the proximal enhancer and *SNAI2* in the Hi-C data, knowing that GRHL2 regulates E genes via CpG methylation and nucleosome remodeling during the intermediate states of EMT/MET [23], we then questioned if the binding of GRHL2 at the *SNAI2* TAD would contribute to the repression of *SNAI2* in the E state by using 3C-qPCR.

Through 3C-qPCR, the interaction frequencies between *SNAI2* TSS and its proximal enhancer were found to be significantly higher in E-like cells (PEO1 and OVCA429) with higher GRHL2 expression, as compared to the M-like SKOV3 and HEYA8 with no GRHL2 expression (*p*_PEO1-SKOV3_ < 0.001, *p*_PEO1-HEYA8_ < 0.001, *p*_OVCA429-SKOV3_ < 0.0001, *p*_OVCA429-HEYA8_ < 0.0001. Fig. 5B). On the contrary, the interaction between *SNAI2* TSS and its distal enhancer in the TAD showed a higher interaction frequency in the M-like HEYA8 than the E state (PEO1) (*p*_PEO1-HEYA8_ < 0.05. Fig. 5B). In PEO1 (E), the distal and proximal enhancers were in closer proximity compared to the M-like cell lines (*p*_PEO1-HEYA8_ < 0.001; Fig. 5B). The proximal enhancer of *SNAI2* coincided with the GRHL2 binding peaks in both PEO1 and OVCA429 (Fig. 5A, D) with low H3K27ac and H3K27me3 marks (Additional file 2: Fig. S6E). Knowing that SNAI1 binds to the *SNAI2* E-boxes and recruits HDAC to repress transcription [54], *SNAI2* repression in PEO1 could result from the endogenous SNAI1 expression and the concomitant H3K27me3 enrichment at the *SNAI2* TSS with the removal of H3K27ac at the proximal and distal enhancers around the close proximity looping (Fig. 5B, D).

In the M-like states SKOV3 (IM) and HEYA8 (M), with both *SNAI1* and *GRHL2* being absent endogenously, the spatial proximity of *SNAI2* TSS with the proximal enhancer decreased significantly. Even as the proximal enhancer exhibited an increasing enrichment of H3K27ac and H3K4me1 marks in SKOV3 and HEYA8 (Additional file 2: Fig. S6E), the accessibility to the enhancer in SKOV3 and HEYA8 was decreased (Additional file 2: Fig. S6E). The factor binding to the proximal enhancer, in the absence of GRHL2, remained to be addressed. In HEYA8, the looping from *SNAI2* TSS to the distal enhancer remained high with the concomitant induction of H3K27ac mark (Fig. 5B, D) and the slightly increased accessibility at the distal enhancer (Additional file 2: Fig. S6E). Taken together, given that the *SNAI2* expression in SKOV3 and HEYA8 was higher compared to PEO1, our data suggested that the interactions among the TSS, proximal and distal enhancers and the local chromatin state might play a predominant role for *SNAI2* transcription.

Interestingly, the distal enhancer interaction was absent in OVCA429, suggesting this interaction may have some cell-type specificity in the regulation of *SNAI2* (Fig. 5B). This loss of interaction also corresponded to the region being inaccessible in OVCA429, with low ATAC-seq peaks at the site where the 3C primer was targeted, as compared to the other cell lines (PEO1, SKOV3, and HEYA8. Additional file 2: Fig. S6E). In OVCA429, there was no endogenous SNAI1 expression to repress *SNAI2* at the distal enhancer with the decrease of H3K27me3 mark around *SNAI2* TSS (Fig. 5A, D). However, GRHL2 binding was still present at the proximal enhancer which remained in spatial proximity to *SNAI2* TSS (Fig. 5B, D). We thus utilized a tetracycline-inducible transcriptional activation (Tet-On) system in OVCA429 shGRHL2 cells that drove the expression of GRHL2 to model the transition between IE and IM states [23]. GRHL2 expression in OVCA429 shGRHL2 Tet-On cells increased the interaction frequency between *SNAI2* TSS and its proximal enhancer but not the distal enhancer, with the concomitant decrease in *SNAI2* expression (Fig. 5C) and the increase in *SNAI1* expression (Fig. 5C, D). Therefore, the interaction between the proximal enhancer and *SNAI2* TSS is regulated by the presence of GRHL2 binding together with the SNAI1 repression at the *SNAI2* TSS without the restoration of the spatial proximity of the distal enhancer.

### Heterogeneity of single-cell 3D genome structure of selected EMT genes

**Fig. 6:**
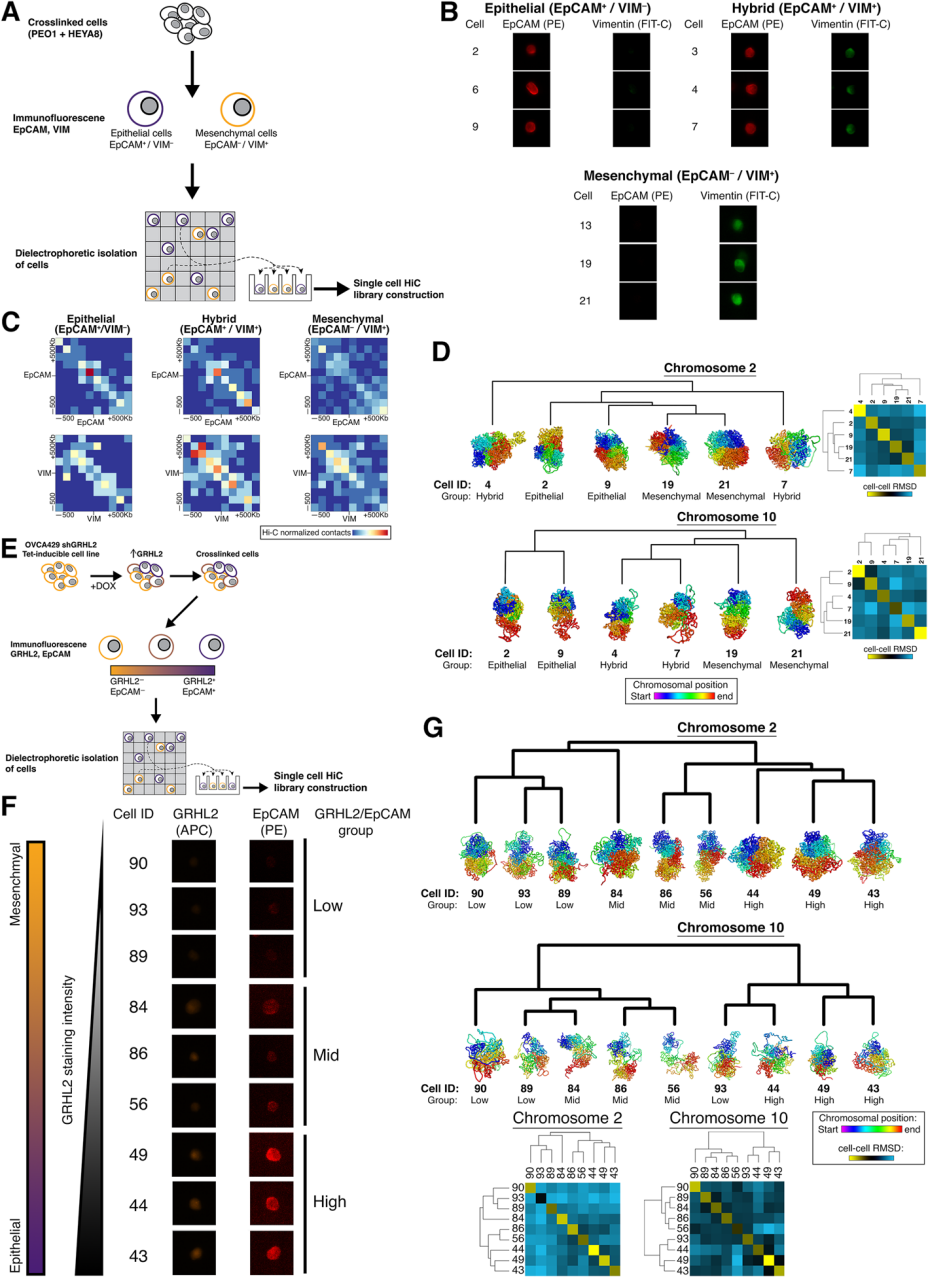
**a** Workflow depicting single-cell isolation by epithelial and mesenchymal markers, followed Hi-C library construction from the single cells isolated from the “PEO1+HEYA8” mixed population of cells. Single cells isolated from the “PEO1+HEYA8” mixed population were classified into (i) Epithelial (EpCAM^+^/VIM^–^), (ii) Hybrid (EpCAM^+^/VIM^+^), and (iii) Mesenchymal (EpCAM^–^/VIM^+^) clusters. Profiles of the 3 clusters: **b** Staining of EpCAM (PE, red) and Vimentin (FIT-C, green); **c** Heatmap of single-cell Hi-C matrix at ± 500 kb of EPCAM or VIM; and **d** genome structures at chromosome 2 and 10. Hierarchical clustering heatmap based on the cell-cell r.m.s.d values at chromosomes 2 and 10 are shown on the right of the genome structures. **e** Workflow depicting single-cell isolation by epithelial and mesenchymal markers, following which Hi-C library construction from the single cells isolated from OVCA429 shGRHL2 Tet-inducible cell line treated with doxycycline to induce GRHL2 expression. The single cells from OVCA429 shGRHL2 Tet-inducible were classified into (i) low GRHL2/EpCAM, (ii) mid GRHL2/EpCAM, and (iii) high GRHL2/EpCAM as differentiated by the staining intensity of GRHL2 (APC, green) and EpCAM (PE, red). Profiles of the GRHL2 groups: **f** staining profiles. EMT spectrum (orange-purple gradient rectangle) alongside GRHL2 staining intensity (triangle with white-black gradient) indicates how the 3 groups of cells correspond to their EMT states in the spectrum; and **g** genome structures of chromosome 2 and 10. Heatmap of the cell-cell r.m.s.d values at chromosome 2 and 10 of the OVCA429 shGRHL2 Tet-inducible single cells are shown below the genome structures. In **b** and **f**, cell IDs are shown to the left of staining images. In **d** and **g**, color = purple-to-red spectrum denotes chromosome start-to-end. Cells are arranged according to the dendrogram derived from hierarchical clustering of the cell-cell r.m.s.d values

Given the known heterogeneity of EMT transition states along the EMT spectrum [11, 12, 55], the bulk Hi-C might still reflect a plethora of diverse EM states. Deconvolution of the genome-wide chromatin structure at a single-cell level was carried out. There were single-cell Hi-C (scHi-C) techniques established using various cell line models [37, 56–58]. Here, we utilized a novel approach where immunofluorescence staining of the E and M markers could be obtained prior to the isolation of single cells via a dielectrophoretic platform (Fig. 6A). PEO1 and HEYA8 cells were mixed in equal populations prior to crosslinking and staining for EpCAM (E marker) and Vimentin (M marker). The two markers were then used to isolate single cells by the fluorescence intensity of these 2 markers (Fig. 6A. Additional file 2: Fig. S7A). Firstly, in the PEO1/HEYA8 mixed population of cells, besides identifying cell clusters which stained positive for EpCAM and for Vimentin respectively, there was also a cluster of cells which showed double positivity (Fig. 6B. Additional file 2: Fig. S7B – C). These 3 clusters of single cells—“E,” “Hybrid,” and “M”—provided a great opportunity to explore differences in the local chromatin structure at *EPCAM* and *VIM* loci and therefore subjected to single-cell Hi-C analysis. At the *EPCAM* locus, the E cluster had a higher density of short-range interactions (+/− 100 kb interactions) which decreased in the hybrid cluster, and in the M cluster *EPCAM* was interacting to regions further upstream/downstream (+/− 300–500 kb) of the gene (Fig. 6C). At the *VIM* locus, there were lesser chromatin interactions at *VIM* in the E cluster compared to both the hybrid and M clusters (Fig. 6C). In addition, the regions upstream and downstream of *VIM* (+/− 500 kb) also had higher density of chromatin interactions in the hybrid and M clusters (Fig. 6C). Therefore, the 3 clusters of single cells exhibited variability in the local chromatin structure at both *EPCAM* and *VIM*, as well as the upstream/downstream regions of the genes. Unlike the bulk Hi-C data, Hi-C contacts identified in single cells represent physical DNA-DNA contacts between distinct genomic regions, making it possible to measure the similarity of the single-cell 3D genome structures of *EPCAM* and *VIM* at their respective chromosomes. Between the single cells of the E, hybrid, and M clusters, there was cell-cell variability in the 3D genome structure of chromosome (chr) 2 where *EPCAM* is situated, whereby the clustering of the r.m.s.d (root-mean-square deviation) values did not identify the 3 clusters (Fig. 6D). Clustering of the r.m.s.d values of the 3D genome structures of chr10, where *VIM* is situated, showed that the genome structures in the E cluster were distinct from the hybrid and M clusters (Fig. 6D). According to the gene density of E and M genes per 10-Mb windows across the chromosomes, in relation to the average Pearson correlation of compartment PC1 to gene expression, chr10 had a lower density of EMT genes but with higher average of correlation between chromosomal compartment changes and expression (Additional file 2: Fig. S7D). For chr2, despite having higher density of EMT genes than chr10, there was lower correlation between chromosomal compartmental changes and gene expression which could explain why the 3D genome structure of chr10 performed better to cluster the EMT states.

In addition, scHi-C libraries from OVCA429 shGRHL2 Tet-inducible cells were also constructed (Fig. 6E). It was shown previously that the induction of GRHL2 in this system did not change *VIM* expression [23], while *EPCAM* was shown to be a direct target of GRHL2 [22]. Hence, GRHL2 and EpCAM were stained in the OVCA429 shGRHL2 Tet-inducible cells to assess the heterogenous EM states along this MET spectrum switching in between the intermediate states. In the OVCA429 shGRHL2 Tet-inducible system, a heterogenous response to GRHL2 induction were observed at the single-cell depth, with 3 groups of cells showing differential staining intensity of GRHL2 and EpCAM that could be classified into (i) low GRHL2/EpCAM, (ii) mid GRHL2/EpCAM, and (iii) high GRHL2/EpCAM (Fig. 6F. Additional file 2: Fig. S7B). We then assessed if the 3D genome structures at chr2 and chr10 were able to distinguish these 3 groups of cells. Clustering of the r.m.s.d values obtained from the 3D genome structure at chr2 and chr10 of the OVCA429 shGRHL2 Tet-inducible cells consistently separated the high GRHL2/EpCAM cells from the rest (Fig. 6G). For chr2, the 3 high GRHL2/EpCAM cells were co-clustered with 2 mid GRHL2/EpCAM cells (Fig. 6G). This suggested that there was an association between the gradual increase in EpCAM protein expression and the changes in chromatin conformation at chr2 in these single cells, which was resolved in the single-cell Hi-C data obtained from the “PEO1+HEYA8” mixed population. For chr10, the 3 single cells belonging to the high GRHL2/EpCAM group were co-clustered with 1 low GRHL2/EpCAM cell while the rest of the low and mid GRHL2/EpCAM cells were clustered together (Fig. 6G). In sum, our data showed that the high GRHL2/EpCAM cells could be consistently separated from the rest from the chromatin conformation of chr2 and chr10.

## Discussion

One conceptual pitfall of understanding the regulation of EMT has been to assume the suppression of E genes and the activation of M genes not only would occur concomitantly but also would follow the same regulatory trajectory. From the barriers of plasticity during EMT or MET, it has been shown that E and M genes are regulated differently at the epigenetic level [23]. The coupling of different TF binding with the chromatin state changes has further supported the concept that although EMT or MET is reversible, the reversibility does not indicate the existence of a universal path [12, 23]. From the spatial perspective of the 3D genome structure, the regulation of E and M genes is clearly distinct. Firstly, we observed that at the organization of the 3D genome at the higher order of compartments is not the sole contributing factor to the regulation of the EMT genes and processes. This was especially so for E genes where their expression was independent to the compartment state changes. The discordance of compartmental changes to gene expression has also been shown in stem cell differentiation. The covariance between gene expression and PC1 values was calculated with only a small subset (33% concordance in AB, and 34% concordance in BA) of genes that showed concordance of gene expression and compartmental switches [35]. While this was also true for nearly half of the M genes, a selected group of M genes crucial for morphogenesis did show compartmental changes from the inactive B in the E state to the active A compartment in the M state. *CDH2*, the gene coding for N-cadherin, is among this category. This could partly explain why cadherin switching from *CDH1* to *CDH2* only occurs in 50% of cancer cells [11] that the structural changes from the compact chromatin to the more open euchromatin might require more complex coordination. This is similar to what was shown in the genome reorganization during the reprogramming of mouse B cells to iPSCs that the pluripotency genes in the A compartment were upregulated early during reprogramming while those genes that switched between B and A compartments were activated at later stages. The delayed activation of these genes reflected the requirement of prior extensive epigenetic and chromatin structure remodelling [59].

Between the cell lines representing the E and M states (PEO1 and HEYA8), we observed that the TADs were relatively stable which was in line with previous studies reporting conservation of TAD boundaries across different cell types [31, 35, 60, 61], while the chromatin modification patterns are constrained within the TAD boundaries. Our data suggested that the expression of the E genes within the eTADs were regulated by the changes in the histone modifications, switching from an open chromatin state marked by H3K27ac in the E-like cancer cell lines to a more repressed one in the M-like cancer cell lines, with the enrichment in H3K27me3. Interestingly, the M genes and their TADs show less associated with the changes in histone modification between the EMT states, suggesting that another underlying mechanism could contribute to the regulation of the M genes. While the E genes and eTADs were repressed at the histone level in the M states, the M genes in the mTADs were more associated with changes in the local conformation of DNA interactions to the regulatory elements in the M state. Since the M genes did not undergo the same repression mechanism during MET as the E genes would be repressed during EMT, this could explain why E genes could be re-expressed by the epigenetic modifying compounds such as HDAC inhibitors and EZH2 inhibitors [23, 62] and the M genes do not exhibit the corresponding repression in the reverse manner [23]. Therefore, the repression of M genes would require different strategies involving the rearrangement of local DNA looping and contacts.

Within the EMT TADs, rather than binary changes in the local chromatin structure, such as the formation or loss of enhancer-promoter interactions, it is the quantitative changes such as contact frequency between the enhancer and its promoter that influences the transcriptional activity of the EMT gene. This was observed in both *CDH2* and *SNAI2* mTADs, the increase in interaction frequency between the genes and its regulatory elements indeed corresponded to an increase in gene expression, despite the enhancer-promoter interactions still being present even in the E state, albeit at a lower frequency. Although the increase in interaction frequency between the E genes and their regulatory elements was not observed in bulk Hi-C data but via 3C-qPCR, it could be further validated with more 3C-qPCR at other epithelial genes, as the bulk Hi-C provides an aggregate of the chromatin interaction frequency at the 88 epithelial genes. The correlation between enhancer-promoter contact frequency and gene transcription has been shown at the single-cell resolution of *Drosophila* embryos, via genome editing and live imaging [63]. The establishment of enhancer-promoter interactions preceding gene activation has been shown during fly embryogenesis and often requires the release of poised RNA polymerases prior to gene transcription [64, 65]. More recently, in *Drosophila* development through high-resolution imaging of single-cell spatial genomics, enhancer-promoter interactions were found to be still present between different cell fates despite having differential transcriptional activity, and instead the stable enhancer-promoter interactions serves as a scaffolds for gene regulation as the enhancers are activated [66, 67]. Furthermore, evidence of such pre-existing enhancer-promoter loops using Hi-C generated from human cell line models, under different stimuli, have reported the activation of genes which are already in contact with their specific enhancers upon signaling cues, thereby allowing rapid transcription activation [68]. In this context, whether the presence of pre-formed mesenchymal promoter-enhancer loops underlying the dynamic induction of EMT upon signaling cues needs to be addressed in a temporal manner.

Meanwhile, to answer the temporal mechanics of chromatin conformation in the regulation of EMT genes, we used an ovarian cancer cell line Tet-On system to model the transition between the IE and IM states. We observed changes in the interaction frequency between *SNAI2* and its proximal enhancer during MET, which could be regulated by induction of GRHL2. However, the regulation of *SNAI2* by SNAI1 and/or GRHL2, as well as the spatial relationship of *SNAI2* to both its proximal and distal enhancers, remains to be addressed. This suggests that the regulation of *SNAI2* and its local chromatin structure also requires the presence of transcription factors such as SNAI1 and/or GRHL2 at the *SNAI2* locus. The presence of transcription factors (TFs) in influencing the local chromatin structure has been described in cell differentiation [69], and there are various modes of TF action on shaping the 3D genome [70]. In the formation of a functional promoter-enhancer loop, it can occur via a pre-formed loop followed by enhancer activation or TF pairing of activated enhancers to their specific promoters [59, 64]. In addition, using an epidermal differentiation system, it was shown that stable enhancer-promoter interactions (present before and after differentiation) were preferentially associated with cohesin, while those enhancer-promoter interactions that only occur upon differentiation were usually mediated by differentiation-induced transcription factors [71]. In recent studies, by coupling CRISPR with Flow-FISH (flow cytometry and fluorescent in situ hybridization) [72] or single-cell RNA-seq (scRNA-seq )[73], the prediction of enhancer-promoter specificity in gene transcription has been found to be dictated by the enhancer activity (determined by the accessibility and H3K27ac signal) and contact frequency. Similarly, for the *SNAI2* locus, we observed that the distal enhancer in the M-like states could play a bigger role in the regulation of *SNAI2* transcriptional activity, given the increased enhancer activity and contact frequency observed at the distal enhancer, as compared to *SNAI2* proximal enhancer.

We also noted in the repression of *CDH1* TAD, the *CDH1*-enhancer promoter remained present in the M state, albeit at a lower frequency. Although we did not assess interaction frequency between the *CDH1* gene and its enhancers in all the cell lines, we did observe a higher interaction frequency between *CDH1* and its proximal enhancer in PEO1 compared to HEYA8 (Additional file 2: Fig. S6C). In addition, it was previously reported in other EMT models that increased interaction frequency between *CDH1* and its intronic enhancer played a role in the regulation of its expression [24, 25]. Interactions between inactive genes and elements marked by repressive chromatin modifications have been reported in another study suggesting the function of these repressive features as long-range silencers [74]. In consideration of the repression observed in the *CDH1* TAD, future studies could address the role of silencers, in tandem with EMT transcription factors (EMT-TFs), given that EMT-TFs usually coordinate the repression of epithelial genes and induction of M genes during EMT [1, 6]. Using CHi-C (Capture Hi-C) in a mouse model, it was also reported that promoters of active genes tend to interact with elements marked by active chromatin features, while transcriptionally inactive genes preferentially associate with repressive chromatin modifications [75], highlighting the presence of silencers in influencing gene expression.

With studies reporting the existence of heterogenous EMT states in the tumor and the in vivo mouse model [12, 76], we established a method to capture the genome-wide chromatin conformation of single cells while maintaining the ability to stratify these cells by their EMT states. There were scHi-C techniques established using various cell line models [37, 56–58] in which some of these methods uses fluorescence-activated cell sorting (FACS) on single nuclei to characterize the cells by their DNA content and Geminin-immunoreactivity. In the context of characterizing cells by their EMT states, the phenotypic information of EMT markers expressed intracellularly or at the cell surface is required. In addition, we observed higher density of chromatin interactions occurring at the *VIM* locus in mesenchymal-like cells, similar to the bulk Hi-C results in which mesenchymal genes tend to interact more frequently with its regulatory elements. The epithelial-like cells, on the other hand, had lesser chromatin interactions at the single-cell depth. To further deconvolute the heterogeneous EMT states occurring during EMT, we constructed scHi-C libraries using a GRHL2 Tet-inducible system, where we observed the changes in chromatin conformation at chr2, harboring *EPCAM*, were able to distinguish the single cells by the staining intensity of EpCAM which was associated with the induction of GRHL2. In addition, the genome structures of chr2 and 10, harboring EpCAM and VIM respectively, were able to consistently distinguish the single cells that had the highest expression of both GRHL2 and EpCAM in the GRHL2 Tet-inducible system. By reconstructing and measuring the similarity of 3D genome structures at selected chromosomes in the single cells, the EMT states could be stratified. While in prior studies, such an approach has been used to report the variability of 3D genome structures between single cells of uncharacterized states [37, 56, 57, 77], our data highlights that chromatin conformation at the chromosomal level could be inferred by spatial arrangement of the EMT genes and paves a new path to identify the hybrid states.

## Conclusions

The Hi-C information generated in this study, coupled with available information from public Hi-C datasets, provides an integrated and comprehensive understanding into the epigenetic regulation of the EMT genes in relation to chromatin conformation. This study also presents an approach to reconstruct the 3D genome structure at the single-cell depth in relation to the epithelial and mesenchymal features of each single cell, which enables further exploration of the role of epigenetic regulation in the plasticity of the EMT process. Our data will serve as a valuable avenue for the EMT community to better understand the complexities underlying the regulation of EM plasticity from the spatial genome context.

## Methods

### Cell culture

Ovarian cancer cell lines, PEO1, HEYA8, and OVCA429 shGRHL2 Tet-inducible, used were cultured as previously described [11, 22, 23]. GRHL2 expression was induced in OVCA429 shGRHL2 Tet-inducible cell line using doxycycline (1 μg/ml) and harvested 1 week later for 3C and scHi-C library construction.

### ChIP-seq analysis

ChIP-seq analysis was performed according to ENCODE guideline [78]. Upon quality checking the raw sequence data, the raw fastq files were mapped using bwa v0.7.13-r1126 to hg38 using the bwa-mem algorithm. The mapped files were then processed using Picard v2.5.0 (http://broadinstitute.github.io/picard/) and Samtools v1.3 [79]. Mapped reads were converted to tag using Bedtools v2.25.0 [80]. Quality of ChIP-seq results were checked using strand-cross-correlation analysis from R SPP package v1.14 [81] to ensure the ChIP-seq experiments were successful. Peaks were called using macs2 v2.1.0.20150731 [82] with --nomodel option and annotated using HOMER v4.10.

### RNA-seq analysis

The raw sequence data were mapped to hg38 using STAR 2.5.3a [83] using default parameter settings, and transcripts were quantified using RSEM v1.3.0 [84] and Gencode v30 annotation.

### ATAC-seq

Raw sequencing fastq files were assessed for quality, adapter content, and duplication rates with FastQC v0.11.9 [85], trimmed using NGmerge v0.3 [86] and aligned with bowtie2 v2.4.2 [87] to the human genome using Homo_sapiens.GRCh38 version from Ensembl [88]. The signal tracks were generated using BAMscale v0.05 [89], and the average signal in the eTADs/mTADs were calculated using deepTools v3.5.0 [90] multiBigwigSummary.

### Chromatin conformation capture (3C)

Cells were crosslinked using 1% formaldehyde for 10 min at room temperature before quenching the reaction with 0.125M glycine. 3C was performed on the crosslinked cells as described by Naumova et al. [91, 92]. 3C-qPCR was then carried on the 3C DNA at the regions of interest and normalized to loading controls [93].

### Hi-C

Cells were crosslinked using 1% formaldehyde for 10 min, and the reaction was stopped in 0.125M glycine for 5 min. The crosslinked cells were then used for Hi-C library construction as described by Rao et al. [42]. Libraries were then sequenced on an Illumina HiSeq platform with 2× 150 bp paired end reads.

### Hi-C analysis

The Hi-C libraries were mapped, processed, and filtered according to the HiCUP v0.6.1 [94] and checked for quality of each library (Additional file 2: Fig. S1). The sequencing metrics of each library is recorded in supplementary information (Additional file 3: Table S1). PEO1 and HEYA8 Hi-C libraries were sequenced to about 1.2 billion paired end reads each. Common artifacts such as dangling ends, re-ligation fragments, contiguous fragments, and duplicated reads were filtered out. About 74% and 77% of the total reads processed were usable Hi-C contacts in PEO1 and HEYA8, separately. The usable Hi-C contacts results generated from the HiCUP was then analyzed using HOMER v4.10 [95] and HiCExplorer [96–98].

### A/B compartment analysis

Chromosomal compartments, A and B, were identified using HOMER v4.10 [95]. Pearson correlation was used to calculate the correlation between PC1 values and RNA-seq gene expression *z*-scores, to look at the correlation between compartmental changes and gene expression. Sub-compartments (A.1.1, A.1.2, A.2.1, A.2.2, B.1.1, B.1.2, B.2.1, and B.2.2) were called using CALDER [44] (https://github.com/CSOgroup/CALDER).

### TADs and loops analysis

The TADs and loops of the Hi-C data used in this study were identified using HiCExplorer v3.6 [96–98] with the default parameters at 50 kb resolution. Aggregate TAD analysis was performed using FAN-C v0.9.17 [99]. To aggregate the contacts between EMT genes and the non-EMT genes of the same TAD, “hicAggregateContacts” from HiCExplorer with the option “—outFileObsExp” was used to obtain the interaction *z*-scores and the aggregate matrix. The interaction *z*-scores from the center pixels of the aggregate matrix were used to compare the differences in chromatin interaction frequency between PEO1, A549, and HEYA8. For statistical analysis, two-sided Student’s *t* test was used. We used “cooler dump” from cooler v0.8 [100] to extract the observed/expected values from the Hi-C data (cooler format) at interacting loci that were validated by 3C-qPCR in *CDH1* and *CDH2* TADs.

### Single-cell Hi-C

Cells were crosslinked using 2% paraformaldehyde for 5 min before the reaction was quenched with glycine. After which the cells were washed with 1 ml PBS before incubation with 0.5 ml of blocking buffer (5% BSA, 0.1% Tween-20 in PBS) at room temperature for 15 min. After centrifugation of the cells at 2200*g* for 5 min at 4 °C, the cells are resuspended in 100 μl of incubation buffer (0.5% BSA, 0.1% Tween-20). The antibodies used for staining were as follows: (i) anti-EpCAM antibody conjugated with PE (sc-71059 PE, Santa Cruz Biotechnology), (ii) anti-Vimentin antibody conjugated with FIT-C (sc-32322 FIT-C, Santa Cruz Biotechnology), (iii) anti-GRHL2 (HPA004820, Sigma-Aldrich) with secondary antibody Alexa Fluor 647-conjugated anti-rabbit (A21245). The amount of the antibodies used for staining was as recommended in their respective datasheets. The cells were first stained for EpCAM for 30 min in the dark at room temperature and washed with PBS before permeabilization with 0.2% TritonX-100 for 15 min at room temperature. The cells are then washed with PBS before staining it for Vimentin for 30 min in the dark at room temperature. For GRHL2 staining, the cells were washed with PBS after staining for Vimentin and subsequently stained for GRHL2 at 30 min in the dark at room temperature. The secondary antibody Alexa Fluor 647-conjugated anti-rabbit (A21245) was then added to the cells after washing once with PBS and incubated at 30 min at room temperature in the dark. The crosslinked and stained cells, at a maximum of 20,000 cells, were then loaded onto the DEPArray™ NxT system for isolation of single cells (Menarini Silicon Biosystems) [101] following the protocol instructions. CellBrowser™ (Menarini, Silicon Biosystems, Bologna, Italy) analysis software, integrated into the DEPArray™ system, was then used view and select cells from the particle database according to multiple criteria, based on qualitative and quantitative marker evaluation and cell morphology. The scan settings used for detection of the immunofluorescence markers in CellBrowser™ (Menarini, Silicon Biosystems, Bologna, Italy) analysis software are shown in the generated DepArray™ reports (Additional file 9: Single cell isolation reports.zip). The fluorescence images of the single cells are also shown in the generated DepArray™ reports (Additional file 9: Single cell isolation reports.zip). The isolated single cells were subsequently used for construction of single-cell Hi-C libraries using the Ampli1^TM^ WGA Kit (Menarini Silicon Biosystems), with minor modifications to the kit protocol. In particular, the cells were lysed using reduced volumes of Hi-C lysis buffer [42]. MboI was used to digest the DNA, before biotin-fill and ligation, to capture the Hi-C contacts. The Hi-C DNA was then obtained, following the protocol stated in the Ampli1^TM^ WGA Kit. The single-cell Hi-C libraries were then sequenced on an Illumina HiSeq platform with 2× 150 bp paired end reads.

### Single-cell Hi-C analysis

The single-cell Hi-C libraries were mapped, processed, and filtered according to NucProcess [57], for subsequent 3D genome structure model analysis. The 3D genome structures in the single cells were reconstructed using NucDynamics [102] with the default parameters, and the comparison of the 3D structures between the single cells was calculated using the Nucleome processing and analysis toolkit [57, 102] (Nuc_tools, available at https://github.com/tjs23/nuc_tools). scHiCExplorer [98] was used to generate the cool matrices representing the epithelial, hybrid, and mesenchymal cell clusters, which was used to plot the Hi-C matrix at +/− 500 kb of EpCAM and Vimentin locus.

## Supplementary Information

**Additional file 1.** Graphical abstract.

**Additional file 2: Figures S1-7**.

**Additional file 3: Table S1**: Hi-C library statistics.

**Additional file 4: Table S2**: Hi-C compartments and gene ontology information relating to Fig. 2B-C.

**Additional file 5: Table S3**: Changes in Hi-C compartments across cancer cell lines. Gene ontology information of epithelial genes in stable and dynamic compartments, relating to Fig. 2D.

**Additional file 6: Table S4**: Changes in Hi-C compartments across cancer cell lines. Gene ontology information of mesenchymal genes in stable and dynamic compartments, relating to Fig. 2D.

**Additional file 7: Table S5**: Pearson correlation analysis, relating to Fig. 3F and Fig. S4D.

**Additional file 8.** Uncropped images, relating to Fig. S2.

**Additional file 9.** DEPArray™ NxT system generated reports for single-cell isolation. The reports contain the parameters used for immunofluorescence and the images of single cells captured by the system.

**Additional file 10.** Review history.

## Data Availability

We downloaded all histone ChIP-seq datasets for MCF-7, PANC1, and A549 from the ENCODE portal [38, 103] (https://www.encodeproject.org/) with the following file accession numbers: ENCFF692SZU, ENCFF096GIM, ENCFF960KET, ENCFF384KMQ, ENCFF999CRP, ENCFF238AHH, ENCFF611UYK, ENCFF997VYW, ENCFF683OAH, ENCFF714IMC, ENCFF747ZKE, ENCFF168ZZS, ENCFF470UYO, ENCFF556EUL, ENCFF913JED, ENCFF285FMN, ENCFF763ADD, ENCFF243YAC, ENCFF107RUY, ENCFF292PQI, ENCFF076CJL, ENCFF839GWH, ENCFF675MQQ, ENCFF114ODP, ENCFF482SGU. Histone ChIP-seq data for ovarian cancer cell lines (PEO1, OVCA429, SKOV3, and HEYA8) were downloaded from NCBI’s Sequence Read Archive [104, 105] (http://www.ncbi.nlm.nih.gov/sra) with the GEO accession number GSE71019 [106]. Hi-C datasets for PANC1 and A549 were also downloaded from the from the ENCODE portal [38, 103] with the following file accession numbers: ENCFF817XOP, ENCFF876LKL, ENCFF896AJW, ENCFF012YJD, ENCFF039FYU, ENCFF479RSE, ENCFF473XVG, ENCFF706REZ, ENCFF622FZJ, ENCFF796FXG, ENCFF231WIS, ENCFF479TQN, ENCFF935APO, ENCFF470IOJ, ENCFF998HPF, ENCFF720PMQ, ENCFF456YXR, ENCFF862MJI, ENCFF520CXI, ENCFF834ELO. The Hi-C data for MCF7 was downloaded from NCBI’s Sequence Read Archive [104, 105] with the GEO accession number GSE118712 [107]. The Hi-C data for PEO1 and HEYA8 have been deposited in NCBI’s Sequence Read Archive [104, 105] and are accessible through GEO Series accession number GEO: GSE201919 [108]. RNA-seq data for the cell lines MCF7, PANC1, A549, and HEYA8 were downloaded from Cancer Dependency Portal (DepMap) under “DepMap Public 20Q2” release [109]. RNA-seq data for PEO1 was download from GEO GSE117765 [110]. Single-cell Hi-C data used in this study have been deposited in NCBI’s Sequence Read Archive [104, 105] and are accessible through GEO Series accession number GEO: GSE201919 [108]. The related DepArray™ generated reports which contains the parameters used for immunofluorescence detection and the immunofluorescence images of single cells captured using the CellBrowser™ analysis software have been submitted to Figshare. DOI: 10.6084/m9.figshare.19732567 [111]. Additional data that support the findings of this study are available from the corresponding author, R.Y.H, upon reasonable request.
