# Supplementary figures and images for: 3D genome organization in the epithelial-mesenchymal transition spectrum
Source: Genome Biol. 2022 May 30;23:121. doi: 10.1186/s13059-022-02687-x (PMC9150291; doi:10.1186/s13059-022-02687-x)

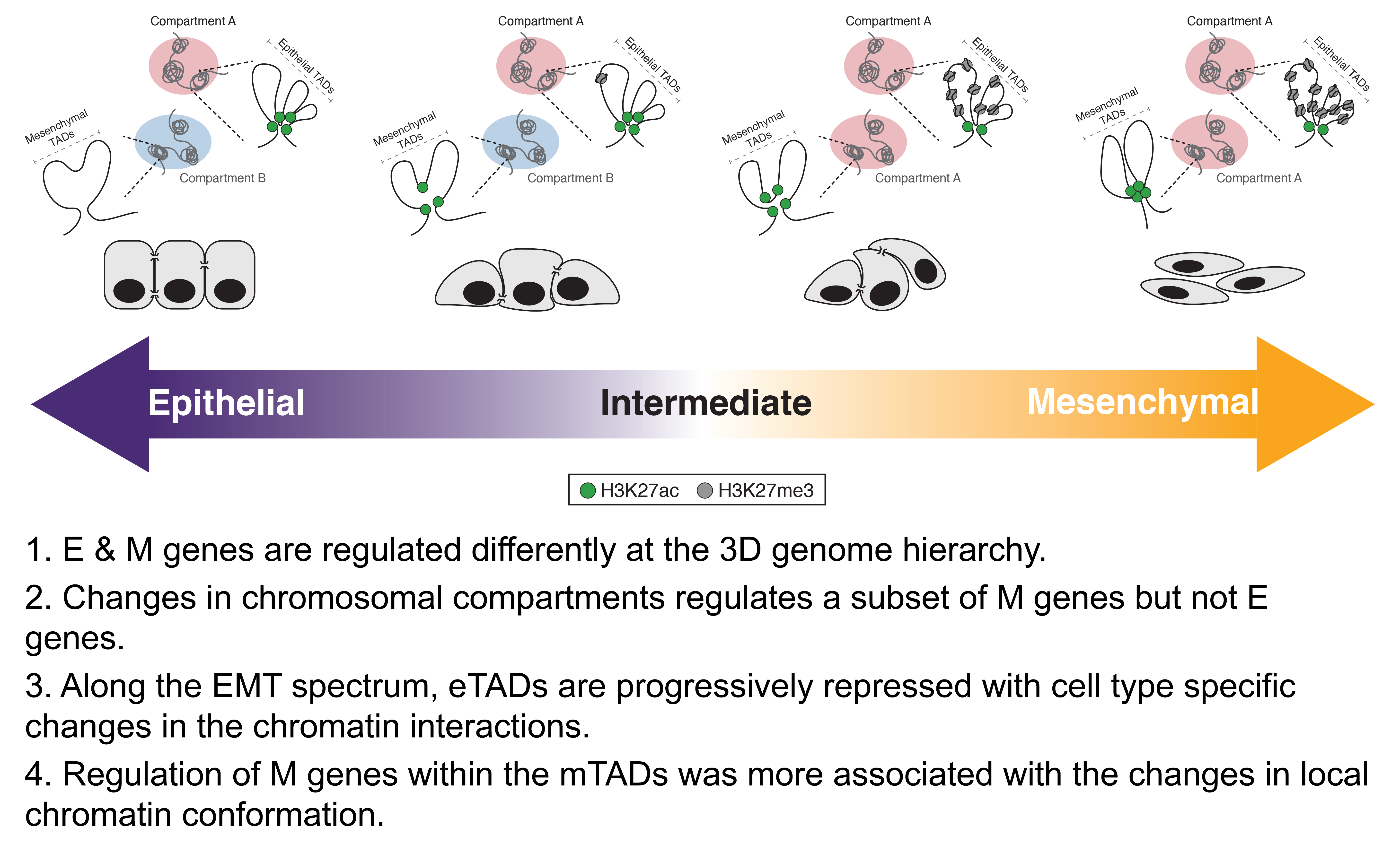

Supplement: Supplementary file 1 — Additional file 1. Graphical abstract. [file 13059_2022_2687_MOESM1_ESM.png]

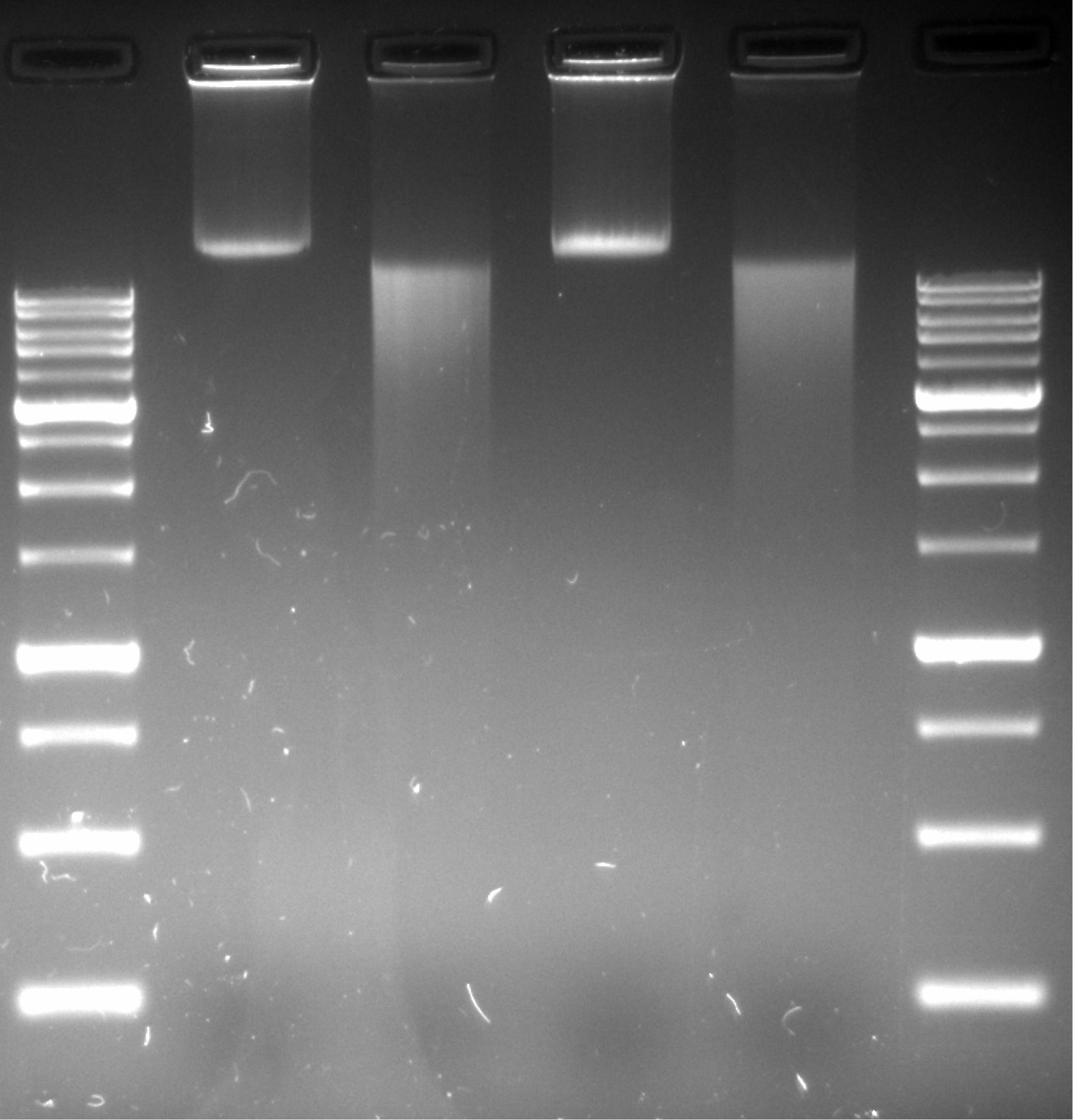

Supplement: Supplementary file 8 — Additional file 8. Uncropped images, relating to Fig. S2. [file 13059_2022_2687_MOESM8_ESM.zip › Additional file 8_Fig S2_uncropped images/Fig S2a_HEYA8 Hi-C Digestion QC_uncropped.tif]

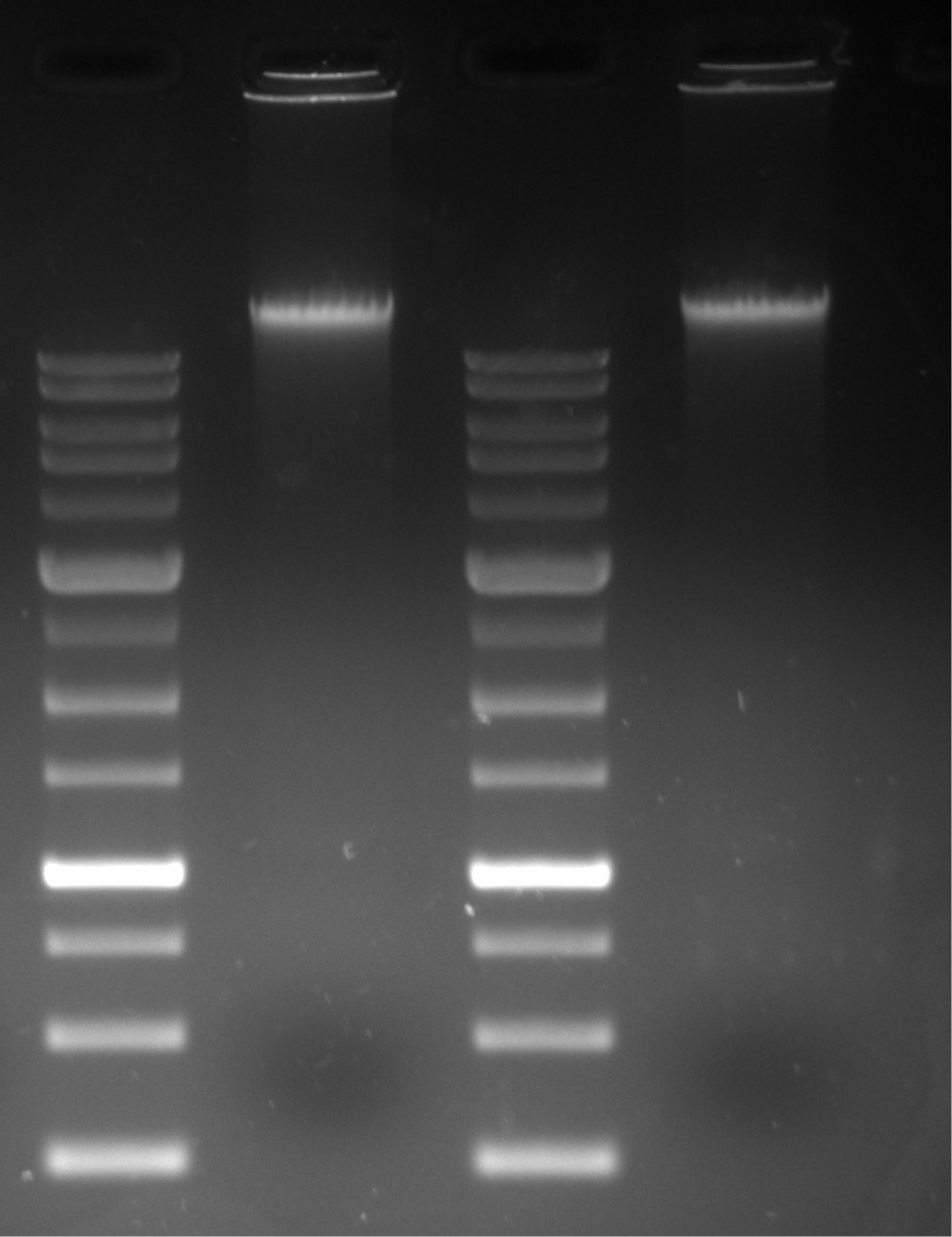

Supplement: Supplementary file 8 — Additional file 8. Uncropped images, relating to Fig. S2. [file 13059_2022_2687_MOESM8_ESM.zip › Additional file 8_Fig S2_uncropped images/Fig S2a_HEYA8 Hi-C ligation QC_uncropped.tif]

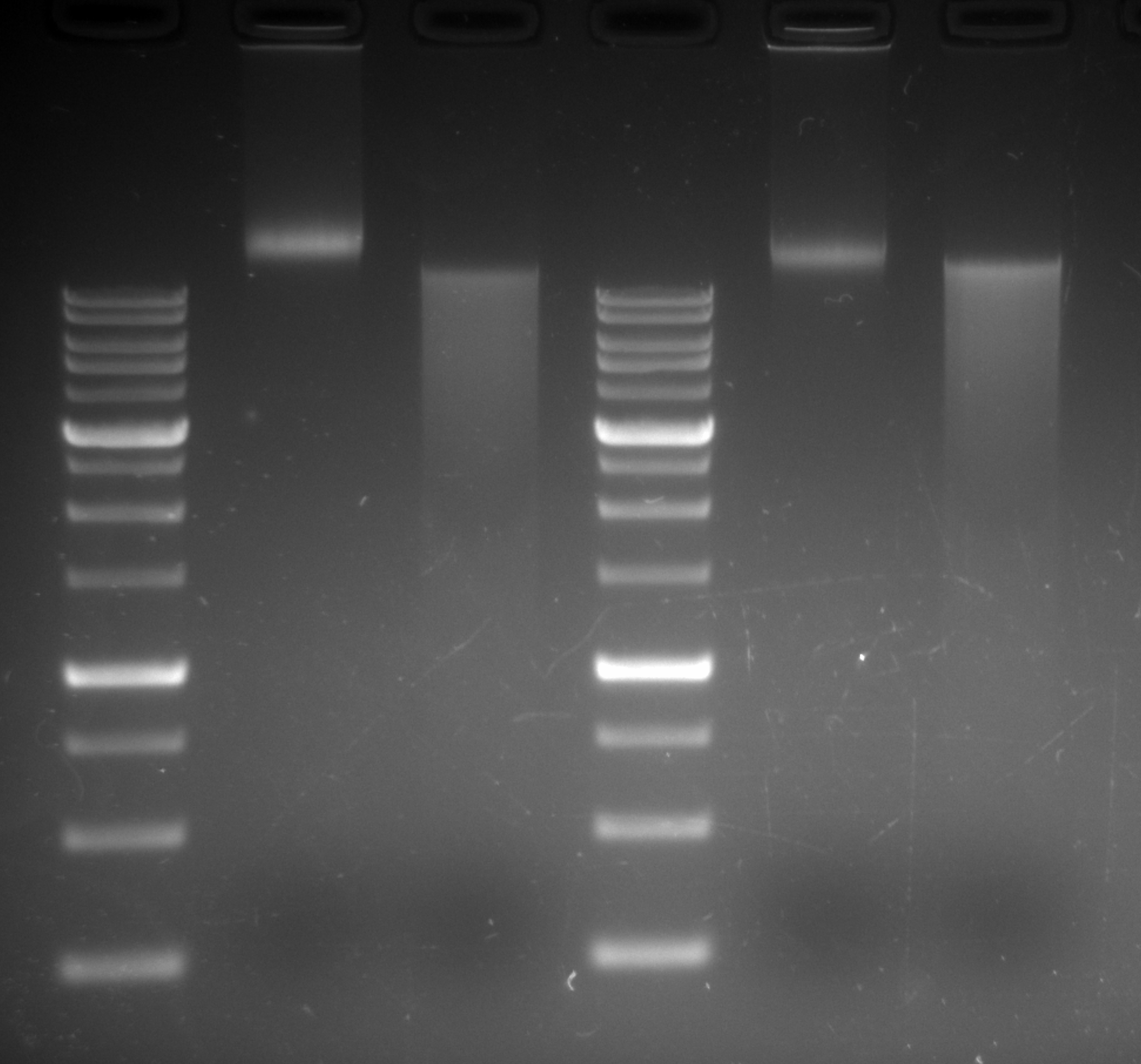

Supplement: Supplementary file 8 — Additional file 8. Uncropped images, relating to Fig. S2. [file 13059_2022_2687_MOESM8_ESM.zip › Additional file 8_Fig S2_uncropped images/Fig S2a_PEO1 Hi-C Digestion QC_uncropped.tif]

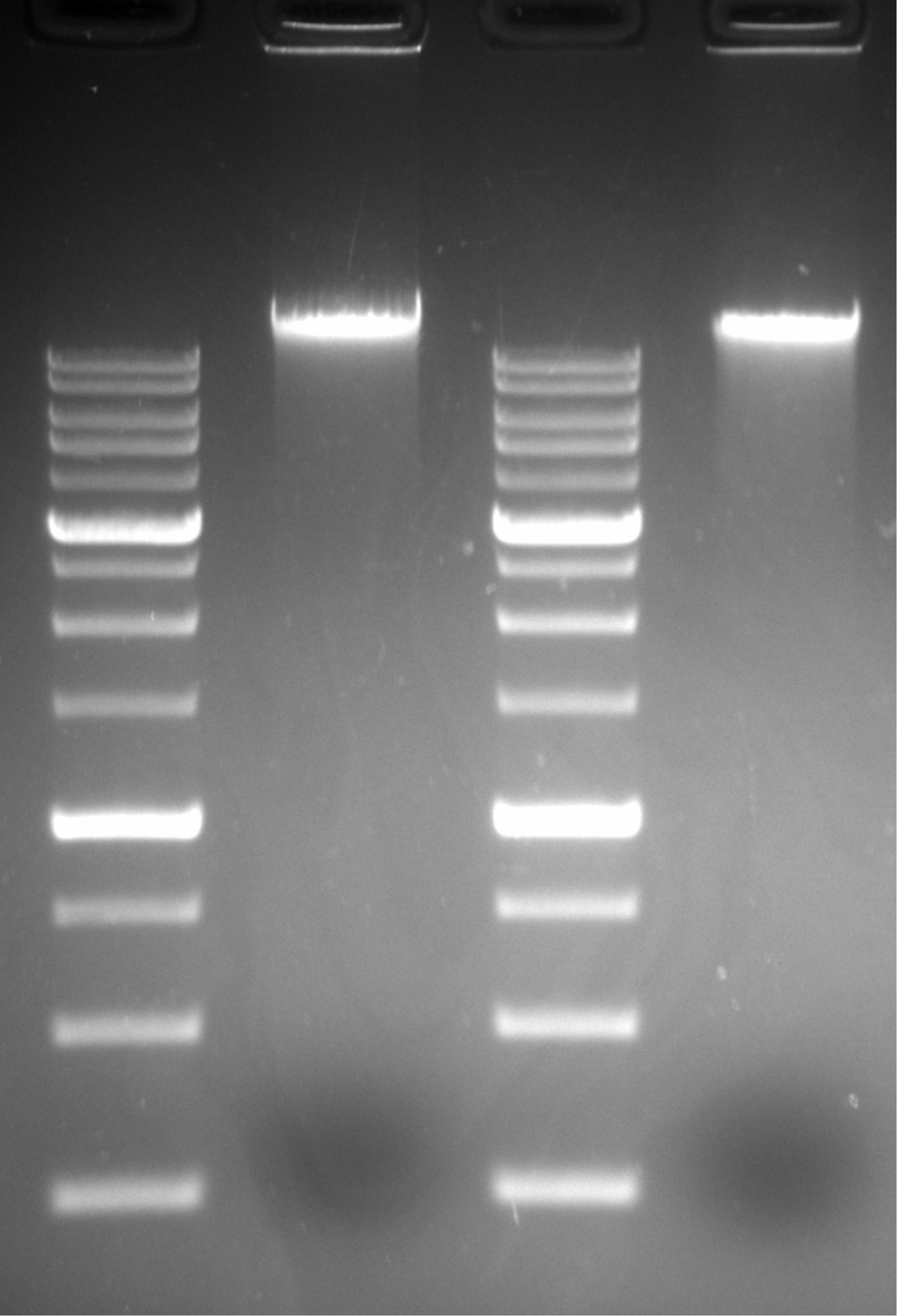

Supplement: Supplementary file 8 — Additional file 8. Uncropped images, relating to Fig. S2. [file 13059_2022_2687_MOESM8_ESM.zip › Additional file 8_Fig S2_uncropped images/Fig S2a_PEO1 Hi-C ligation QC_uncropped.tif]

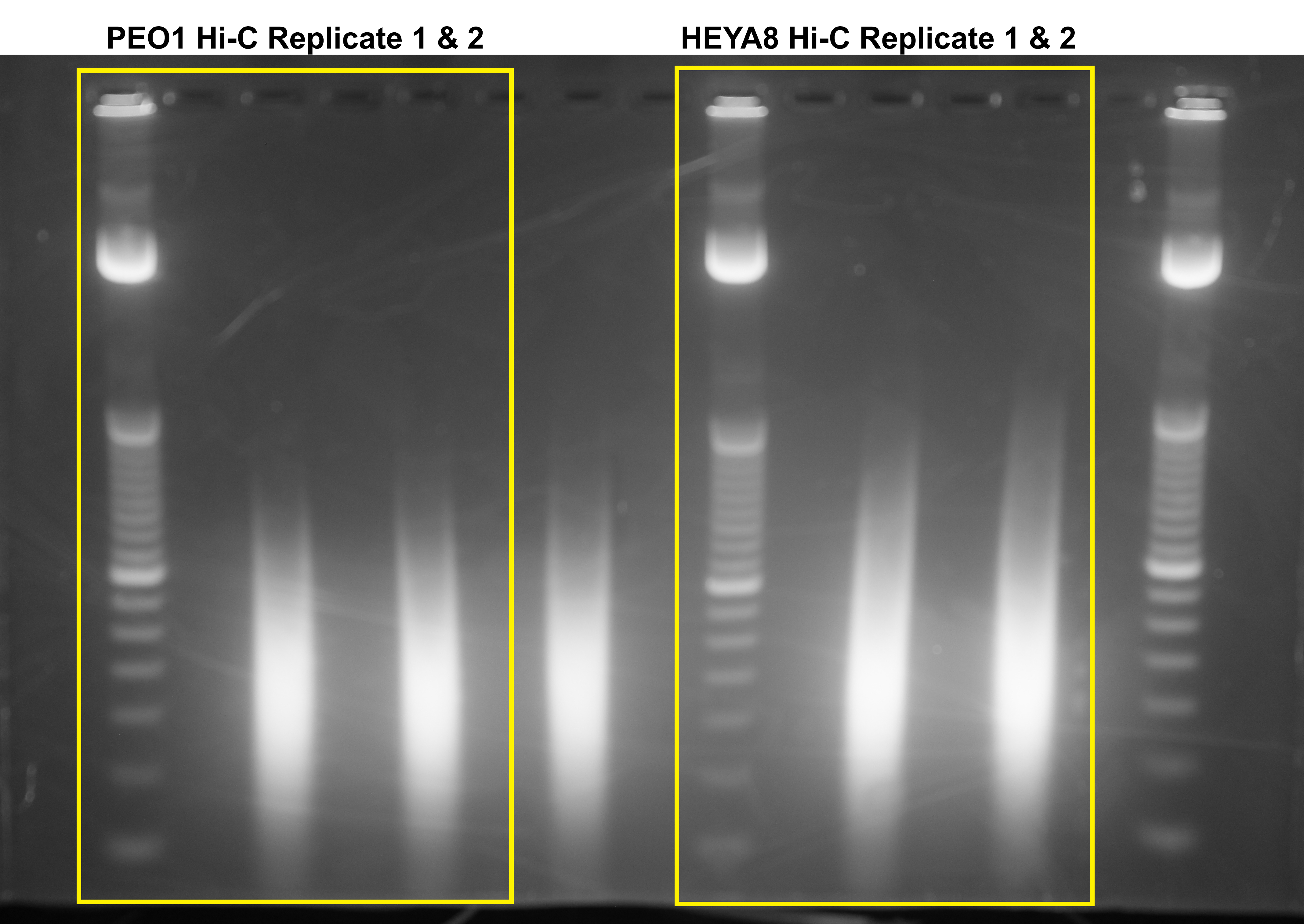

Supplement: Supplementary file 8 — Additional file 8. Uncropped images, relating to Fig. S2. [file 13059_2022_2687_MOESM8_ESM.zip › Additional file 8_Fig S2_uncropped images/FigS2d_uncropped image.png]
