# Supplementary material for: 3D genome organization in the epithelial-mesenchymal transition spectrum
Source: Genome Biol. 2022 May 30;23:121. doi: 10.1186/s13059-022-02687-x (PMC9150291; doi:10.1186/s13059-022-02687-x)
Supplement: Supplementary file 2 — Additional file 2: Figures S1-7. [file 13059_2022_2687_MOESM2_ESM.pdf]

Fig S1

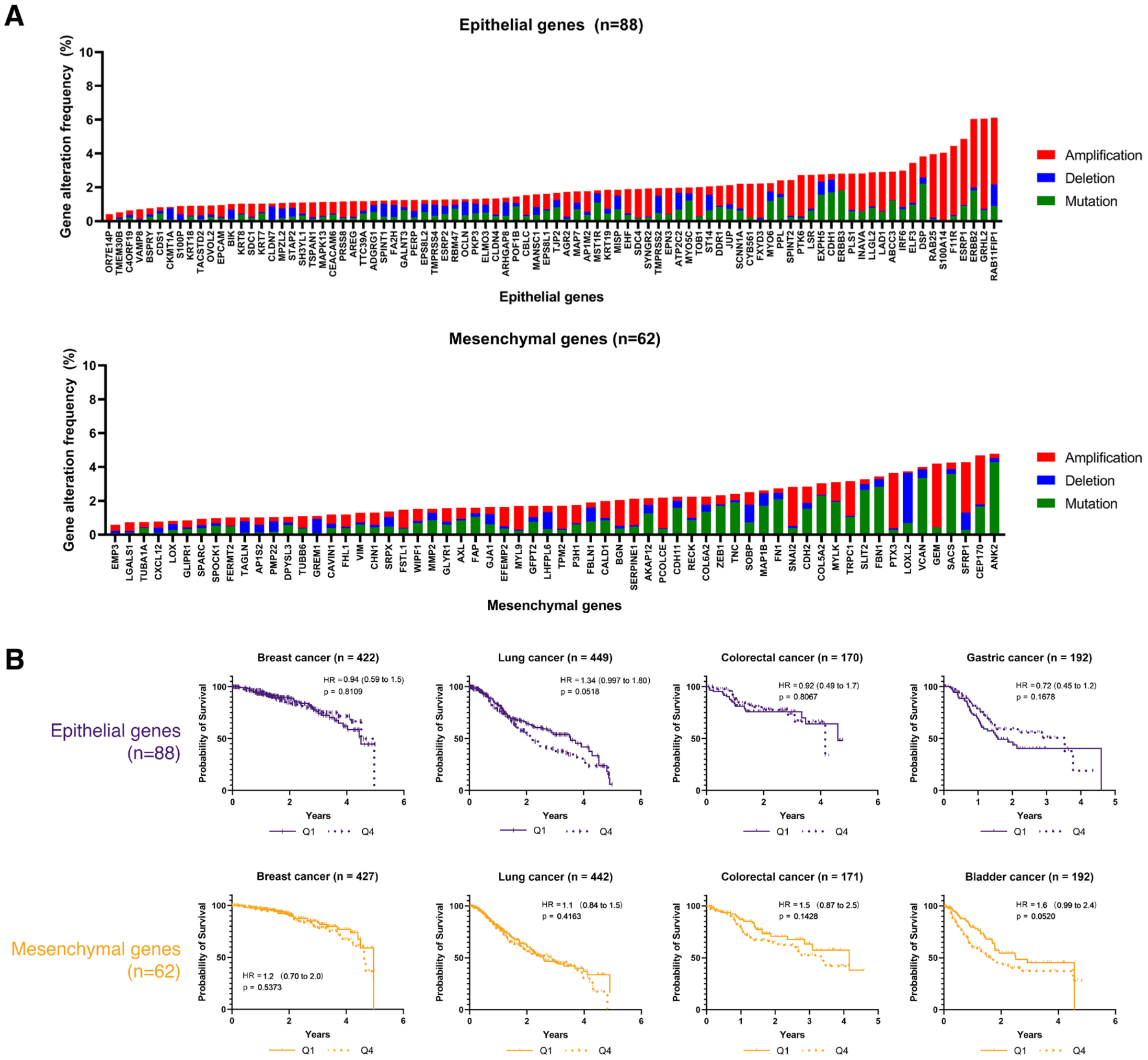

Fig S1. (a) Bar chart representations of the gene mutation and copy-number alteration frequency of EMT genes across pan-cancer tumours (n=8,510). (b) Kaplan-Meier survival analysis of epithelial (purple) or mesenchymal (orange) genes for 1st quartile (Q1 – solid lines) vs 4th quartile (Q4 – dotted lines) in breast, lung, colorectal, bladder, and gastric cancers.

Fig S2

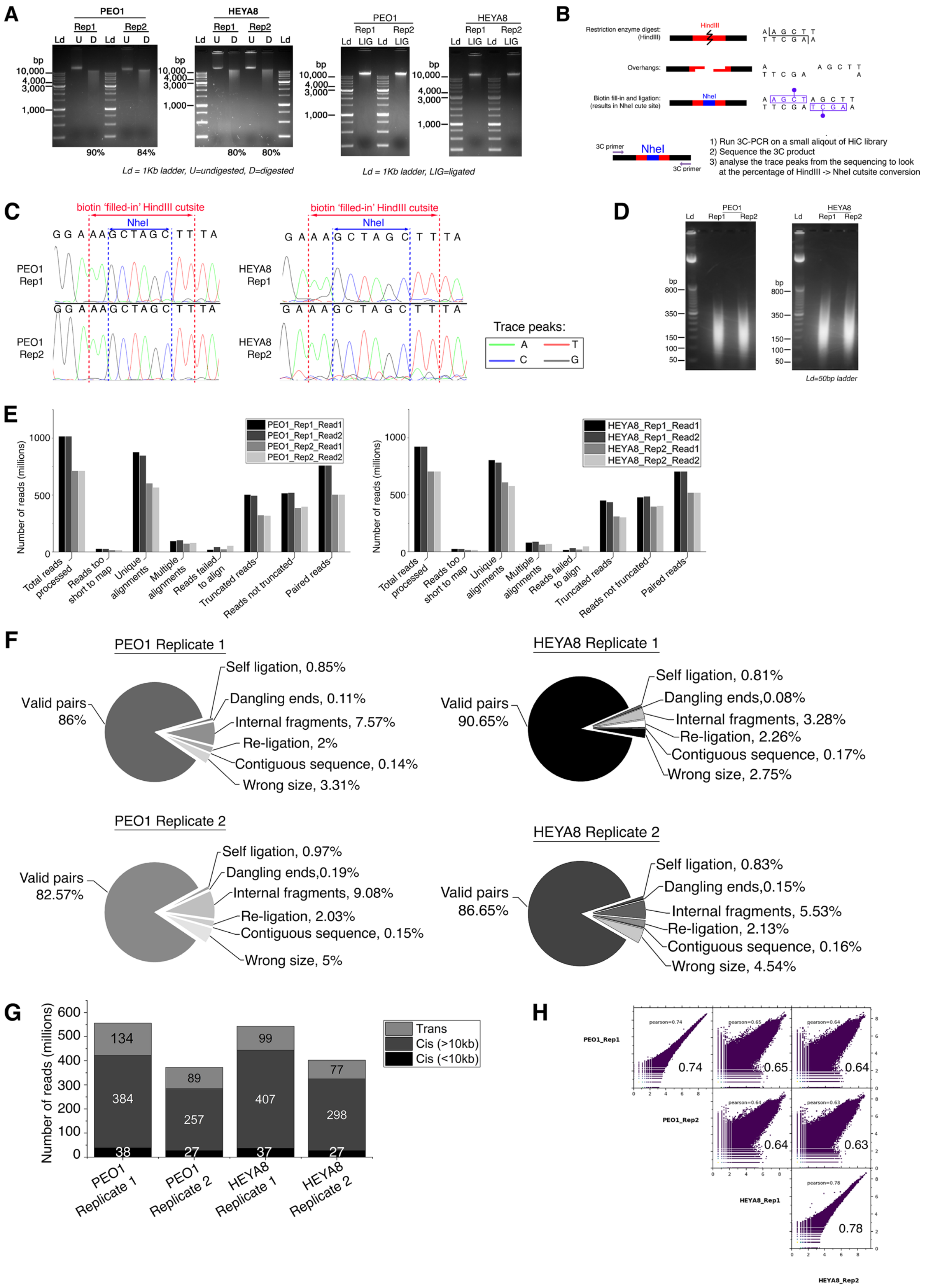
A
PEO1
Rep1
Rep2
Ld U D Ld U D
HEYA8
Rep1
Rep2
Ld U D U D Ld
90%
84%
80%
80%
Ld = 1Kb ladder, U=undigested, D=digested
PEO1
HEYA8
Ld LIG Ld LIG
Ld = 1Kb ladder, LIG=ligated
B
Restriction enzyme digest: (HindIII)
HindIII
Overhangs:
Biotin fill-in and ligation: (results in NheI cute site)
NheI
3C primer
1) Run 3C-PCR on a small aliqout of HiC library
2) Sequence the 3C product
3) analyse the trace peaks from the sequencing to look at the percentage of HindIII -> NheI cutsite conversion
C
biotin 'filled-in' HindIII cutsite
PEO1 Rep1
PEO1 Rep2
HEYA8 Rep1
HEYA8 Rep2
Trace peaks:
D
Ld=50bp ladder
E
Number of reads (millions)
PEO1_Rep1_Read1
PEO1_Rep1_Read2
PEO1_Rep2_Read1
PEO1_Rep2_Read2
HEYA8_Rep1_Read1
HEYA8_Rep1_Read2
HEYA8_Rep2_Read1
HEYA8_Rep2_Read2
Total reads processed
Reads too short to map
Unique alignments
Multiple alignments
Reads failed to align
Truncated reads
Reads not truncated
Paired reads
F
PEO1 Replicate 1
Valid pairs 86%
Self ligation, 0.85%
Dangling ends, 0.11%
Internal fragments, 7.57%
Re-ligation, 2%
Contiguous sequence, 0.14%
Wrong size, 3.31%
HEYA8 Replicate 1
Valid pairs 90.65%
Self ligation, 0.81%
Dangling ends,0.08%
Internal fragments, 3.28%
Re-ligation, 2.26%
Contiguous sequence, 0.17%
Wrong size, 2.75%
PEO1 Replicate 2
Valid pairs 82.57%
Self ligation, 0.97%
Dangling ends,0.19%
Internal fragments, 9.08%
Re-ligation, 2.03%
Contiguous sequence, 0.15%
Wrong size, 5%
HEYA8 Replicate 2
Valid pairs 86.65%
Self ligation, 0.83%
Dangling ends,0.15%
Internal fragments, 5.53%
Re-ligation, 2.13%
Contiguous sequence, 0.16%
Wrong size, 4.54%
G
Number of reads (millions)
Trans
Cis (>10kb)
Cis (<10kb)
134
384
38
89
257
27
99
407
37
77
298
27
PEO1 Replicate 1
PEO1 Replicate 2
HEYA8 Replicate 1
HEYA8 Replicate 2
H
PEO1_Rep1
PEO1_Rep2
HEYA8_Rep1
HEYA8_Rep2
0.74
0.65
0.64
0.64
0.63
0.78

Fig S2. (a) Gel electrophoresis of digested Hi-C libraries, where 2 replicates of Hi-C libraries were performed in each cell line. Aliquots of the libraries were ran on a 1.2% gel against a 1Kb ladder (bp = basepairs). Replicates 1 and 2 of the Hi-C libraries are represented by 'Rep1' and 'Rep2' respectively. The digested libraries (D) were loaded next to the undigested libraries (U) to estimate the digestion efficiency. The digestion efficiency of the libraries was also checked using qPCR. The Hi-C libraries after proximity ligation step (LIG) were ran against a 1Kb ladder on a 1.2% gel. Replicates 1 and 2 of the Hi-C libraries are represented by 'Rep1' and 'Rep2' respectively. (b) Illustration of biotin fill-in in Hi-C workflow depicts the conversion of HindIII (red) to NheI (blue) to enrich the Hi-C contacts with biotinylated cut sites. (c) Sanger sequencing to check biotin fill in of Hi-C libraries. (d) Sonication profile of Hi-C libraries. (e) Mapping statistics of Hi-C libraries as processed by HiCUP. (f) Filtering of the mapped pair reads in PEO1 and HEYA8 Hi-C libraries. (g) Number of valid chromatin contacts available in PEO1 and HEYA8 Hi-C libraries. (h) Pairwise correlation of replicates 1 and 2 of PEO1 and HEYA8 Hi-C libraries. Pearson correlation of PEO1 Hi-C replicates 1 and 2 is 0.74, and HEYA8 Hi-C replicates 1 and 2 is 0.78. Pearson correlation values between replicates of the two different cell lines are also noted, respectively.

Fig S3

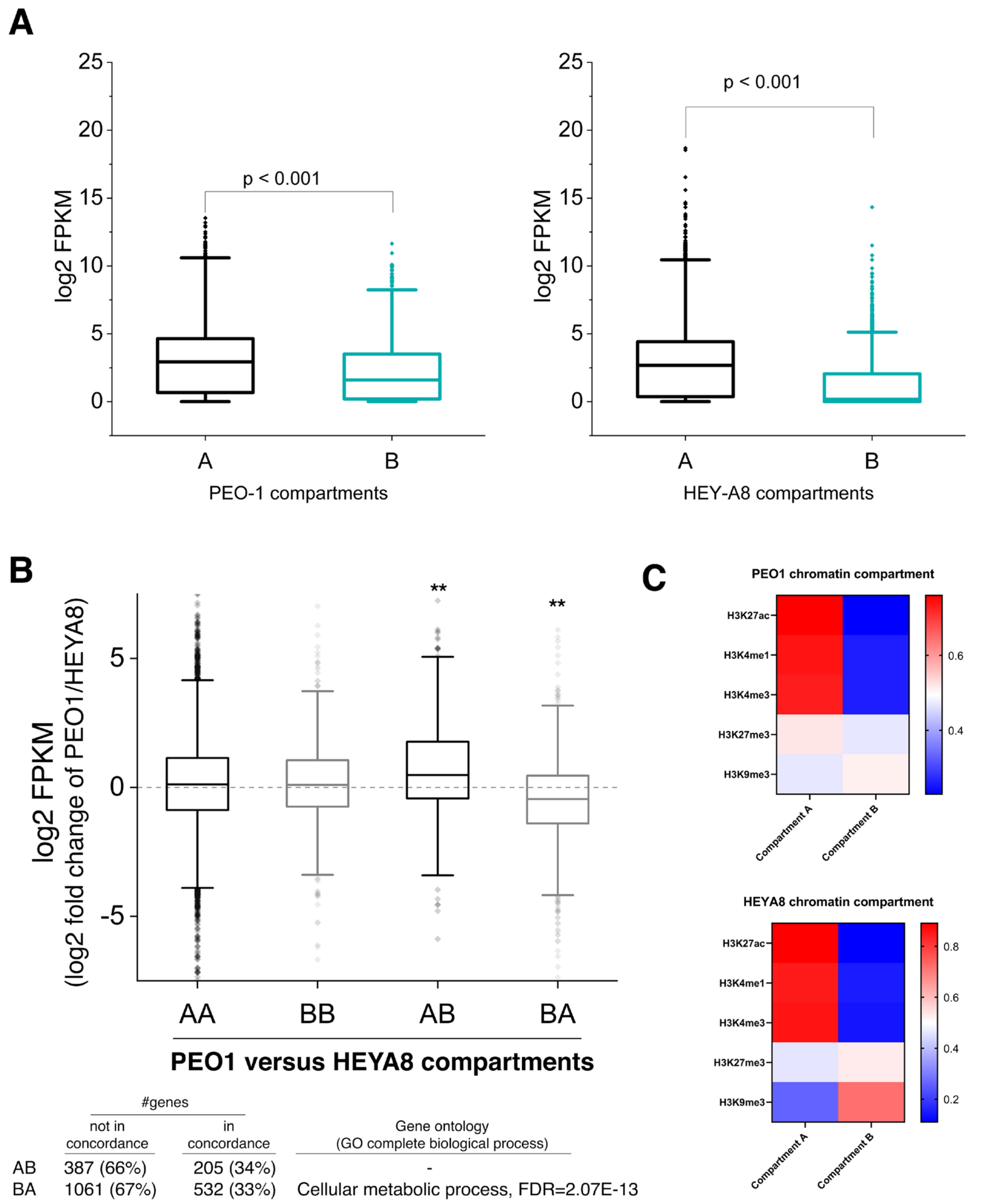

Fig S3. (a) Expression of genes (log2 FPKM; y-axis) in chromosomal compartments (A and B; x-axis) of PEO1 (left) and HEYA8 (right). (b) Fold change of gene expression (log2 FPKM; y-axis) between PEO1 and HEYA8, across the categories of changes in active (A) and inactive (B) chromosomal compartments (x-axis). Gene ontology of the genes in ‘BA’, genes that switched from inactive compartment in PEO1 to active compartment in HEYA8 were enriched in cellular metabolic processes (FDR = 2.07×10^{-13}). (c) Correlation between histone marks (H3K27ac, H3K4me1, H3K4me3, H3K27me3, H3K9me3; y-axis) and chromatin

compartments A and B (x-axis) in PEO1 (top) and HEYA8 (bottom). Coloured heatmap (blue and red scale) indicating the Pearson correlation value.

Fig S4

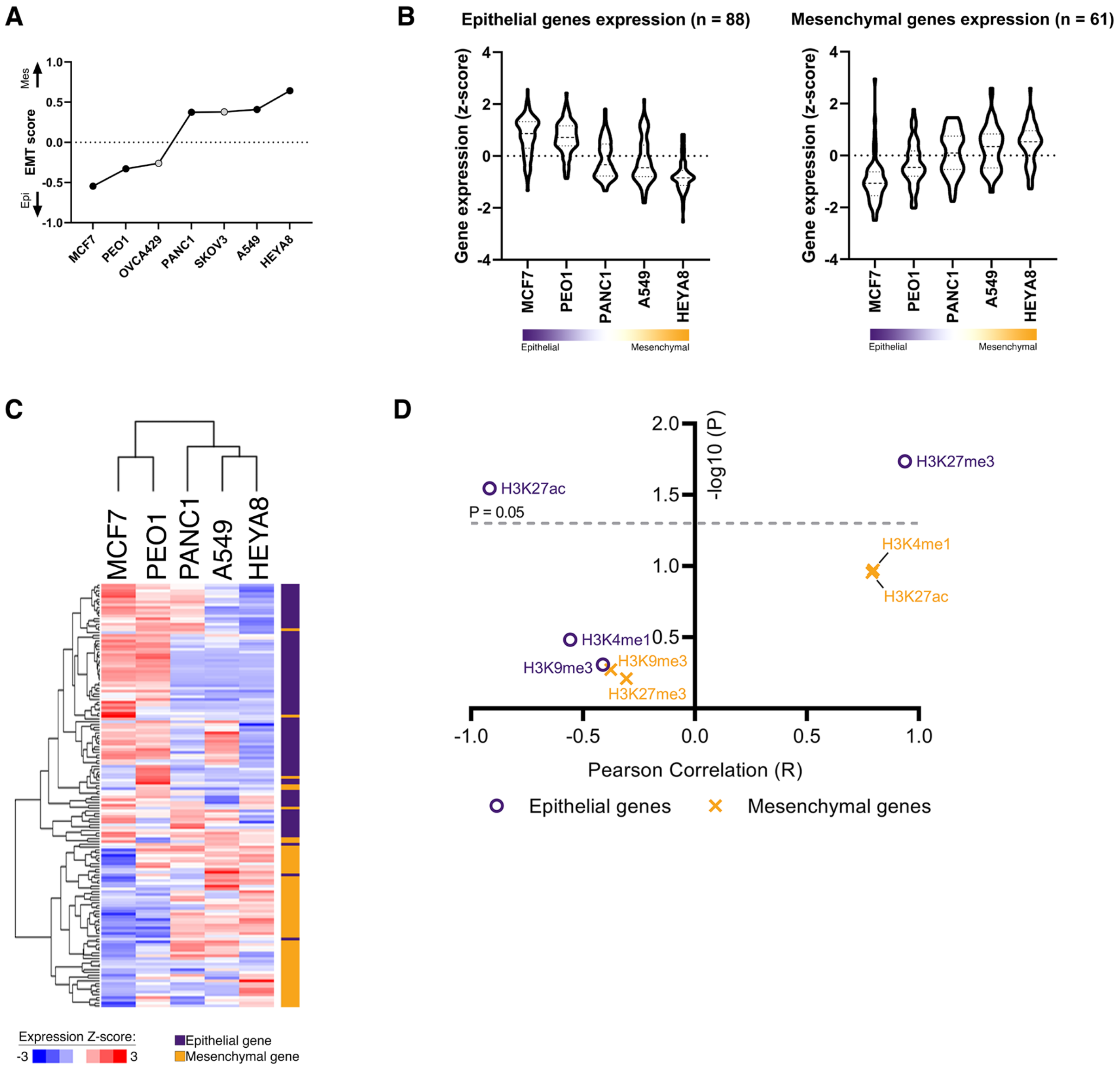

Fig S4. (a) EMT scores (y-axis) of cancer cell lines (x-axis; MCF7, PEO1, OVCA429, PANC1, SKOV3, A549, HEYA8). Epi: epithelial; Mes: mesenchymal (b) Violin plots showing expression of epithelial and mesenchymal genes in z-scores (y-axis) across the cancer cell lines (x-axis; MCF7, PEO1, PANC1, A549, HEYA8). Colour heatmap (purple to yellow) indicating the EMT spectrum. (c) Heatmap of unsupervised hierarchical clustering gene expression of epithelial (purple) and mesenchymal (yellow) genes in z-scores (blue and red colour scale) across the cancer cell lines (MCF7, PEO1, PANC1, A549, HEYA8). (d) Scatter plot showing the significance *p*-value levels (y-axis) and Pearson correlation R values (x-axis) between EMT

scores and average histone signals (H3K27ac, H3K4me1, H3K27me3 and H3K9me3) at epithelial (purple circle) and mesenchymal (yellow cross) genes.

Fig S5

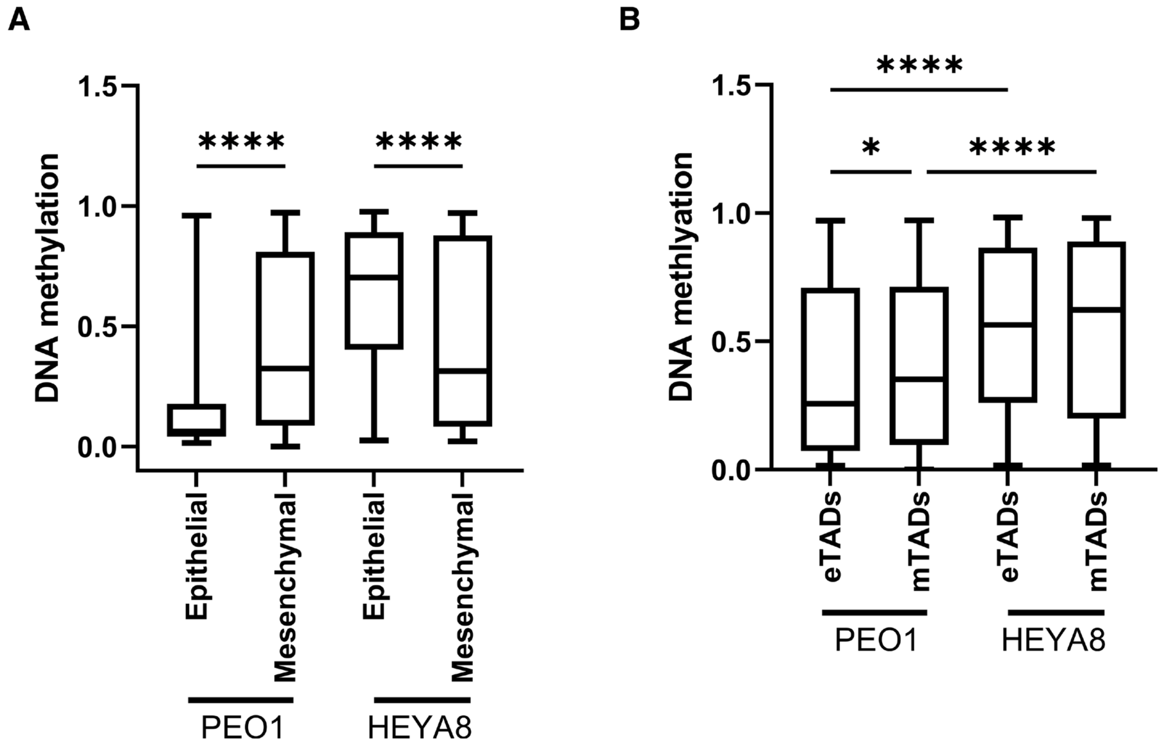

Fig S5. (a) Box plots showing DNA methylation (y-axis) of epithelial and mesenchymal genes within EMT TADs of PEO1 and HEYA8 (x-axis). (b) Box plots showing DNA methylation (y-axis) of epithelial TADs (eTADs) and mesenchymal TADS (mTADs) of PEO1 and HEYA8 (x-axis). **** p<0.0001; * p<0.05

Fig S6

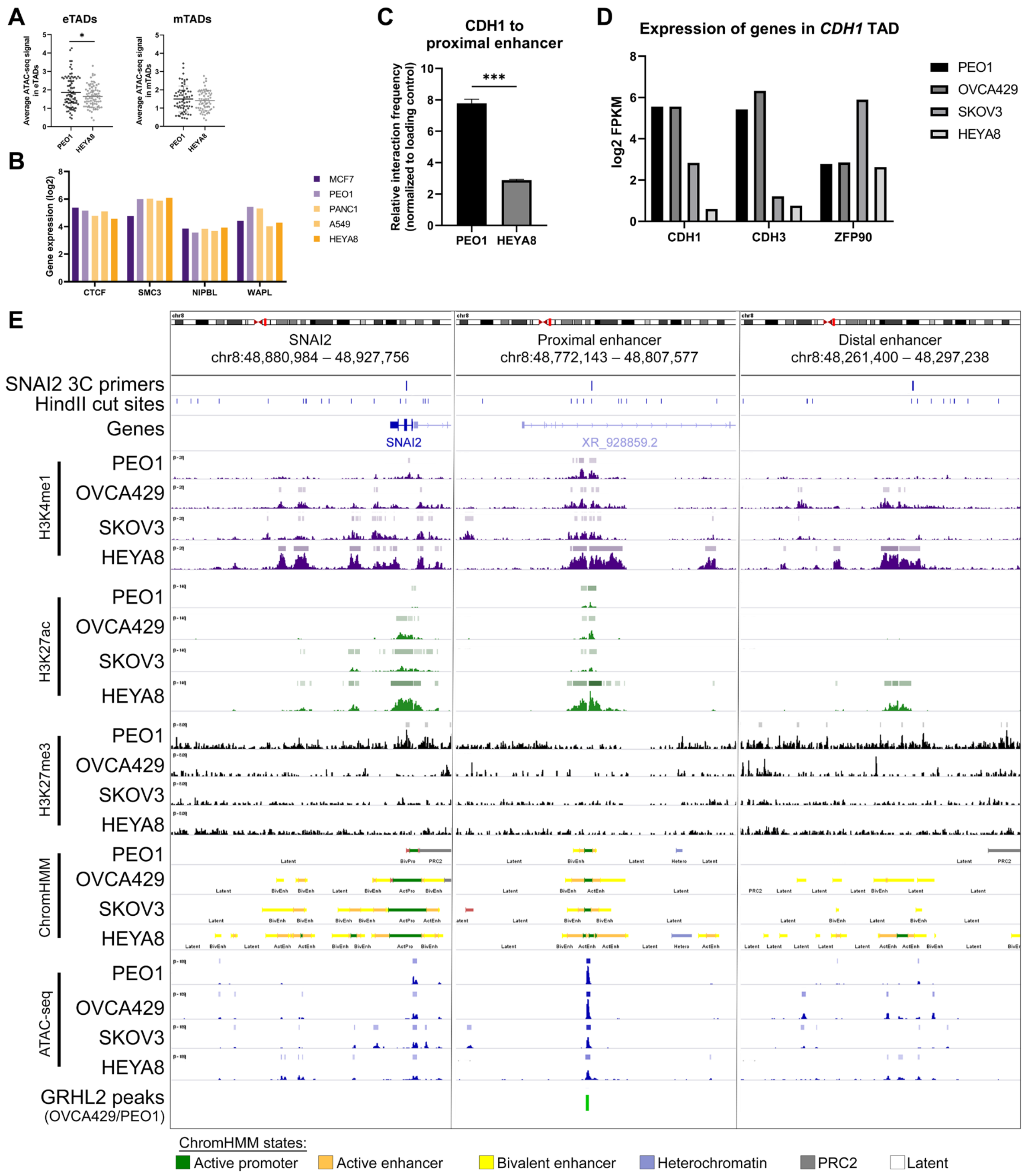

Fig S6. (a) Average ATAC-seq signal (y-axis) in eTADs (left) and mTADs (right) in PEO1 (black) and HEYA8 (gray). **p*<0.05, Student's T-test. (b) RNA-seq log2 gene expression (y-axis) of CTCF, SMC3, NIPBL and WAPL in MCF7 (dark purple), PEO1 (light purple), PANC1 (pale yellow), A549 (light yellow), HEYA8 (dark yellow). (c) Bar chart showing 3C-qPCR normalized interaction frequencies (y-axis) between TSS of *CDH1* and its proximal

enhancer (5,400bp apart) in PEO1 (black) and HEYA8 (gray). ***$p<0.001$, Student's T-test. (d) Bar chart showing log2 FPKM (y-axis) of gene expression levels of CDH1, CDH3, and ZFP90 within the *CDH1* TAD in PEO1 (black), OVCA429 (dark gray), SKOV3 (gray), and HEYA8 (light gray). (e) IGV browser view at 3 regions of SNAI2 locus: SNAI2 gene, SNAI2 proximal enhancer, and SNAI2 distal enhancer showing their respective genomic positions at chromosome 8 (chr8) (top panel and 3rd lane from top), 3C primer sites (1st lane from top), HindIII cut sites (2nd lane from top), and the peaks of histone marks, H3K4me1 (indigo), H3K27ac (green), H3K27me3 (black), across PEO1, OVCA429, SKOV3, and HEYA8. ChromHMM states (active promoter in green, active enhancer in orange, bivalent enhancer in yellow, heterochromatin in blue, PRC2 in gray, latent in white) and the ATAC-seq peaks (blue) in PEO1, OVCA429, SKOV3, and HEYA8 are shown below the IGV browser tracks followed by the GRHL2 binding peaks in both PEO1 and OVCA429 (bottom lane).

Fig S7

A

Single cell HiC libraries statistics

PEO1 + HEYA8 single cells

Cell ID	1	2	3	4	5	6	7	8	9	10	11	12
Total contacts	16,238	152,989	60,249	147,336	17,883	76,609	83,750	47,382	96,943	90,997	87,928	140,931
Ambiguous contacts	-	-	-	-	-	-	-	-	-	-	-	-
Cis near (contacts)	235	45,849	11,132	49,030	428	26,151	28,261	11,261	32,736	29,873	34,433	31,189
Cis far (contacts)	1,173	73,536	41,638	71,880	3,588	32,967	33,427	18,678	41,948	40,386	37,619	81,854
Total cis (contacts)	1,408	119,385	52,770	120,910	4,016	59,118	61,688	29,939	74,684	70,259	72,052	113,043
Trans (contacts)	14,830	33,604	7,479	26,426	13,867	17,491	22,062	17,443	22,259	20,738	15,876	27,888

Cell ID	13	14	15	16	17	18	19	20	21	22	23	24
Total contacts	97,576	22,808	7,237	45,557	13,413	98,327	119,797	29,112	104,708	44,218	139,944	68,425
Ambiguous contacts	-	-	-	-	-	-	-	-	-	-	-	-
Cis near (contacts)	25,100	4,499	59	394	78	23,861	31,532	52	27,449	189	33,439	17,313
Cis far (contacts)	45,746	8,733	439	7,278	930	46,302	56,569	1,661	47,327	3,551	79,276	31,876
Total cis (contacts)	70,846	13,232	498	7,672	1,008	70,163	88,101	1,713	74,776	3,740	112,715	49,189
Trans (contacts)	26,730	9,576	6,739	37,885	12,405	28,164	31,696	27,399	29,932	40,478	27,229	19,236

OVCA429 shGRHL2 Tet-inducible single cells

Cell ID	43	44	49	56	84	86	89	90	93
Total contacts	35,580	49,506	49,519	30,431	59,994	37,394	30,046	27,331	28,062
Ambiguous contacts	-	-	-	-	-	-	-	-	-
Cis near (contacts)	14,769	18,149	15,815	10,092	16,218	12,395	10,353	9,059	6,476
Cis far (contacts)	15,458	21,080	21,289	13,302	22,780	17,944	12,539	12,530	9,745
Total cis (contacts)	30,227	39,229	37,104	23,394	38,998	30,339	22,892	21,589	16,221
Trans (contacts)	5,353	10,277	12,415	7,037	20,996	7,055	7,154	5,742	11,841

B

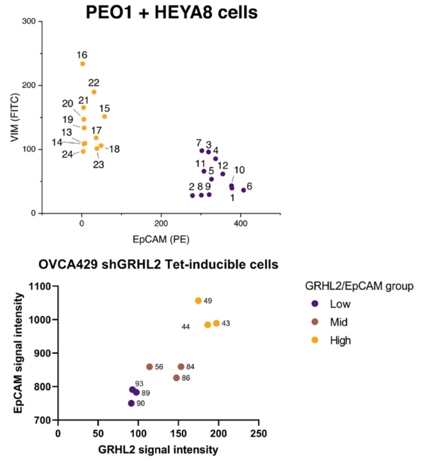

C

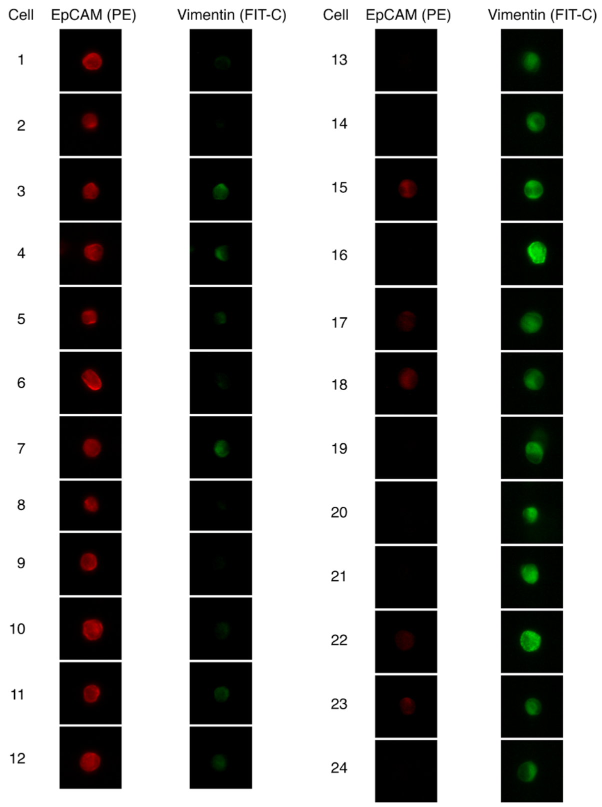

D

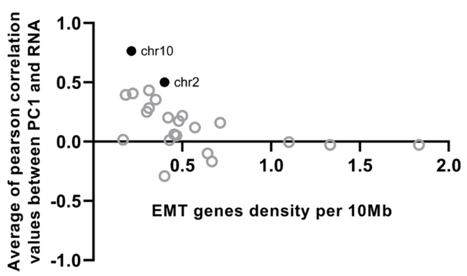

Fig S7. (a) Statistics of single cell Hi-C libraries of mapping and filtering of reads. (b) Scatterplot (top) of the fluorescence staining intensity signals of EpCAM (PE, x-axis) and VIM

(FITC, y-axis) in single cells isolated for Hi-C library construction, from the PEO1+HEYA8 population. Scatterplot (bottom) showing the fluorescence staining intensity signals of EpCAM (PE, y-axis) and GRHL2 (APC, x-axis) in single cells isolated from the doxycycline-treated OVCA429 shGRHL2 Tet-inducible cells. (c) Fluorescence staining images of EpCAM (PE, red) and VIM (FITC, green) in single cells isolated from the PEO1+HEYA8 population. Cell IDs are shown on the right of the staining images. (d) Scatterplot showing the density of EMT genes per 10Mb region on individual chromosomes (x-axis) with the average Pearson correlation values (correlation between chromosomal compartment PC1 and RNA-seq gene expression values) of EMT genes (y-axis) on each chromosome. Chromosomes 2 (chr2) and 10 (chr10) are denoted as black dots, while the other chromosomes are shown as gray circles.
