# Supplementary material for: 3D genome organization in the epithelial-mesenchymal transition spectrum
Source: Genome Biol. 2022 May 30;23:121. doi: 10.1186/s13059-022-02687-x (PMC9150291; doi:10.1186/s13059-022-02687-x)
Supplement: Supplementary file 9 — Additional file 9. DEPArray™ NxT system generated reports for single-cell isolation. The reports contain the parameters used for immunofluorescence and the images of single cells captured by the system. [file 13059_2022_2687_MOESM9_ESM.zip › OVCA429_shGRHL2_tet_DepArrayReport.pdf]

DEPArray™ NxT Run Report not for IVD use

System information

DEPArray™ NxT System Serial Number: D03-001-0069

DEPArray™ NxT System Software Release: 3.5.1.8.1

DEPArray™ NxT System intended for: Research Use Only

Run information

Selected application: CTC-RUO-FIXED

Run ID: 150721EDDY02

Run mode: Sorting

DEPArray™ NxT Cartridge: 950007382

DEPArray™ Buffer:

User ID: NCCS

Recovery Container Information: 200ul-tube-rack 200ul-tube-rack-1

Date: 2021 / 07 / 15

Scan settings	Chip scan				Image analysis		
	Exposure (ms)	Camera Gain	Lamp intensity (%)	Offset (um)	Detection	Analysis	Duplicate removal
**CHIP SCAN 1**							
APC	200	1X	100%	50	Faint Signal	Enable	
FITC	1000	8X	100%	35	Faint Signal	Enable	
PE	1000	8X	100%	35	Faint Signal	Enable	☑
BRIGHTFIELD	3	1X	5%	26	Faint Signal	Enable	

Group 1

id	Group	APC_0	PE_1	FITC_2	BRIGHTFIELD_3	APC_PE_FITC_ BRIGHTFIELD_4	
74	GROUP 1	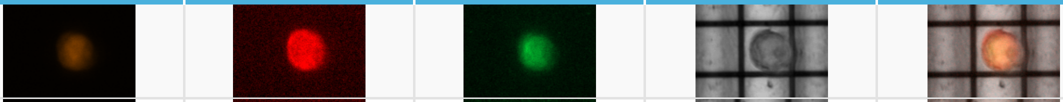					☑
708	GROUP 1	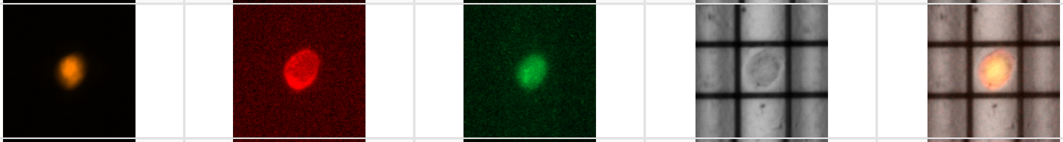					☑
1331	GROUP 1	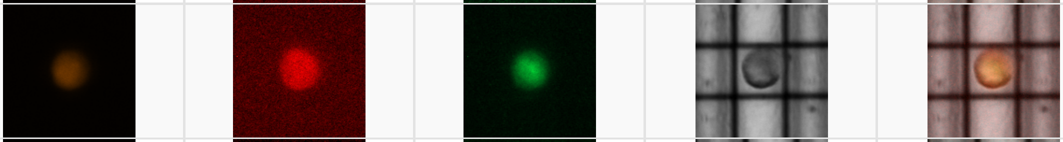					☑
2024	GROUP 1	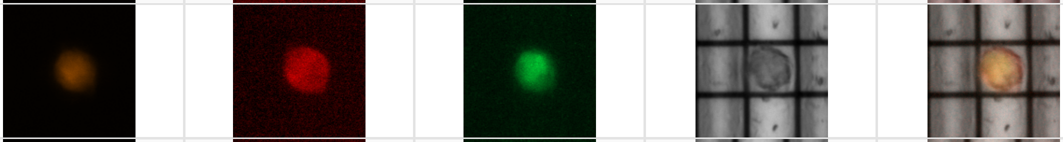					☑
2390	GROUP 1	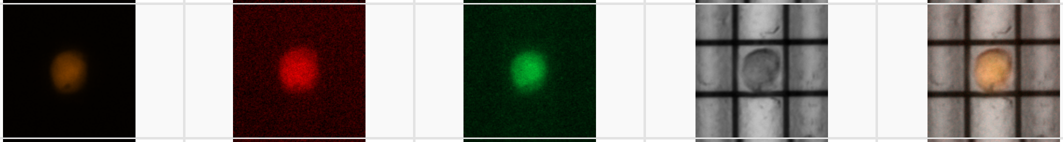					☑
2523	GROUP 1	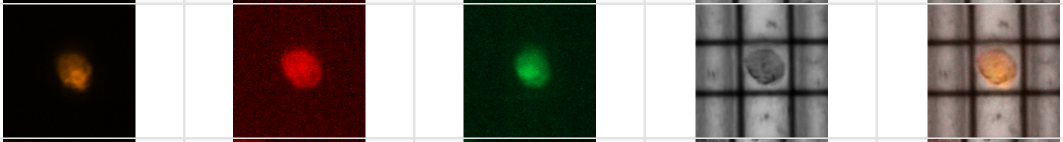					☑
2783	GROUP 1	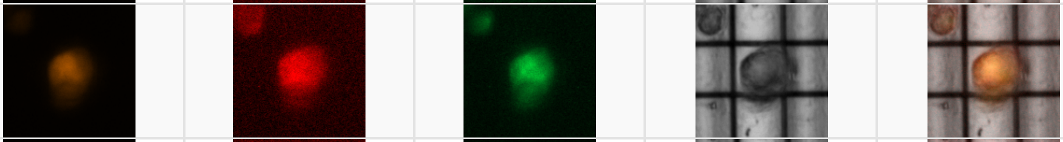					☑
2790	GROUP 1	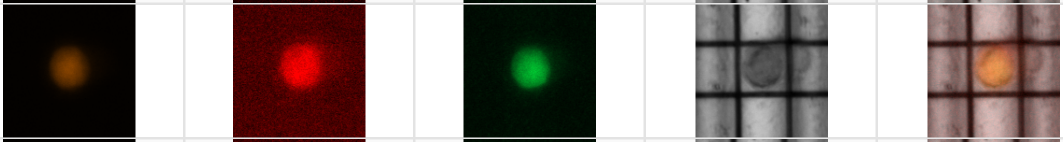					☑
2881	GROUP 1	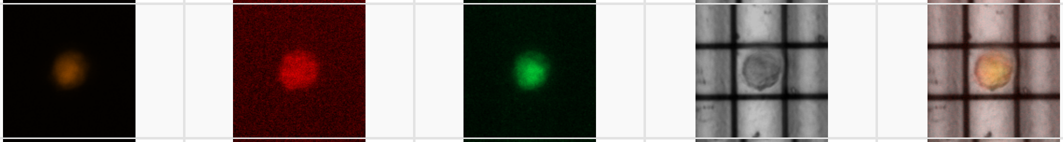					☑
3203	GROUP 1	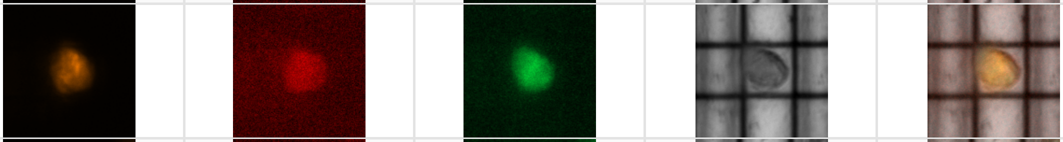					☑
3277	GROUP 1	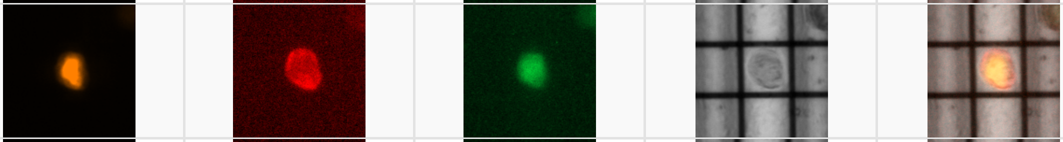					☑
3486	GROUP 1	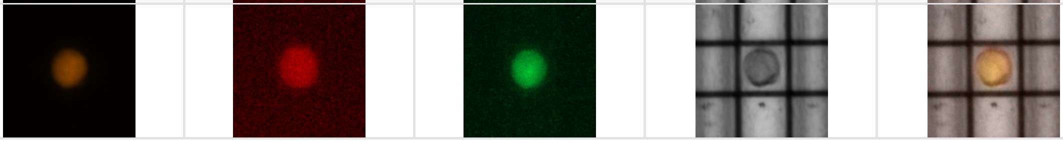					☑

id	Group	APC_0	PE_1	FITC_2	BRIGHTFIELD_3	APC_PE_FITC_ BRIGHTFIELD_4	
3573	GROUP 1						☑
3576	GROUP 1						☑
3667	GROUP 1						☑
3732	GROUP 1						☑
3903	GROUP 1						☑
4832	GROUP 1						☑
5624	GROUP 1						☑
6358	GROUP 1						☑
6445	GROUP 1						☑
6950	GROUP 1						☑
624	GROUP 1						☑
6617	GROUP 1						☑

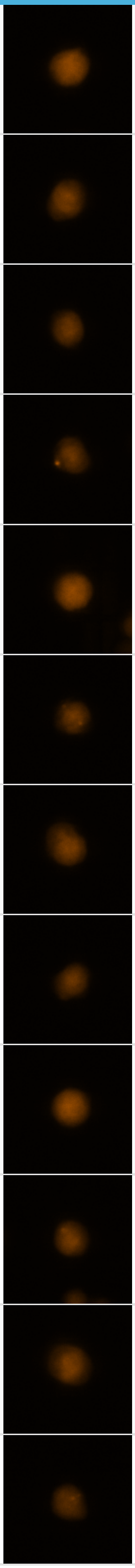
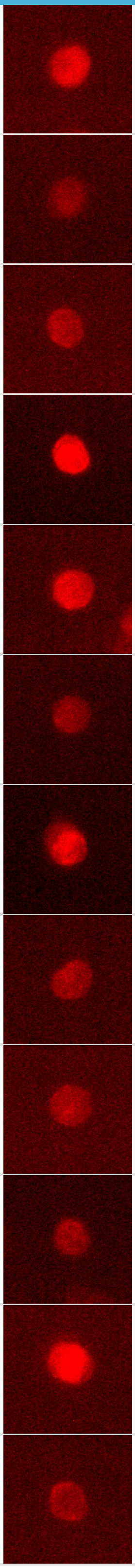
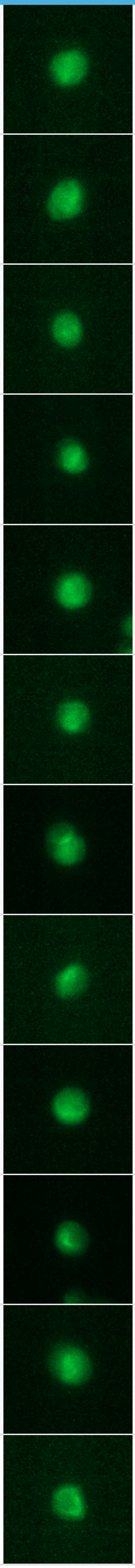
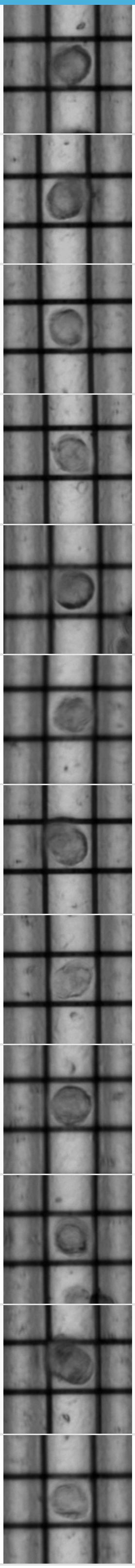
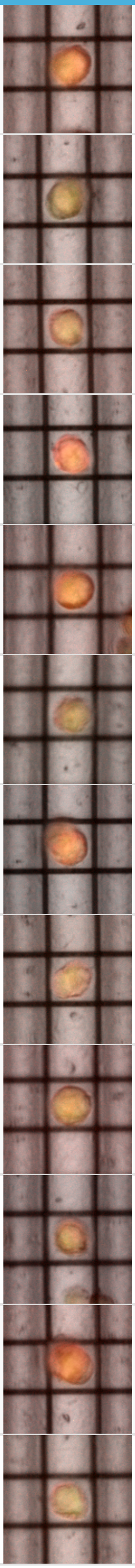

id	Group	APC_0	PE_1	FITC_2	BRIGHTFIELD_3	APC_PE_FITC_BRIGHTFIELD_4	
2319	GROUP 1	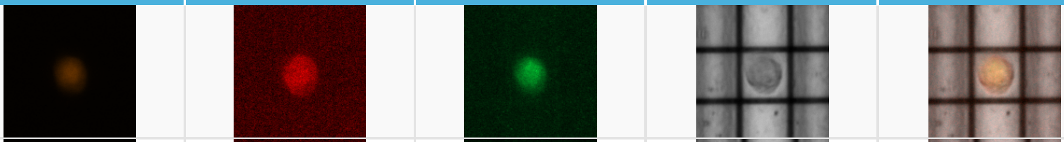					☑
5837	GROUP 1	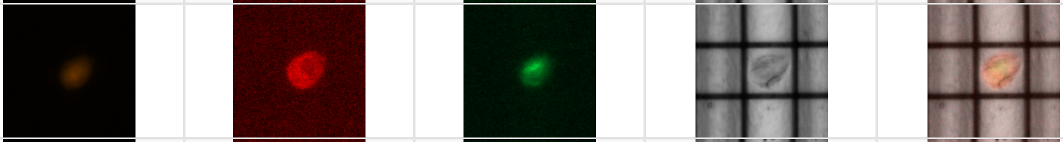					☑
757	GROUP 1	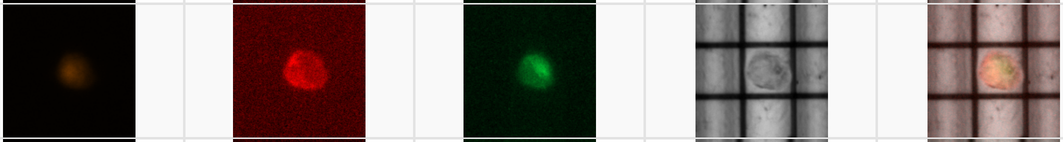					☑
5187	GROUP 1	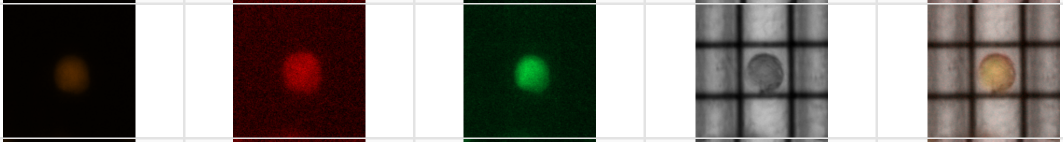					☑
5216	GROUP 1	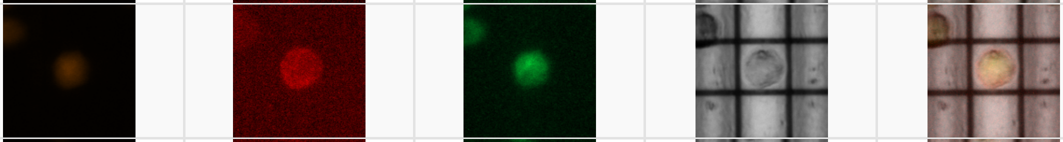					☑
3889	GROUP 1	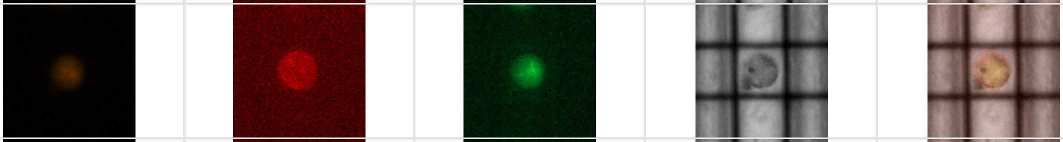					☑
4922	GROUP 1	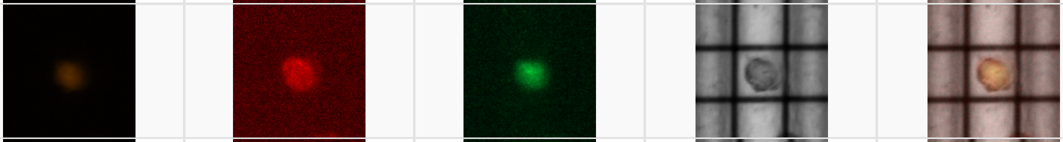					☑
2077	GROUP 1	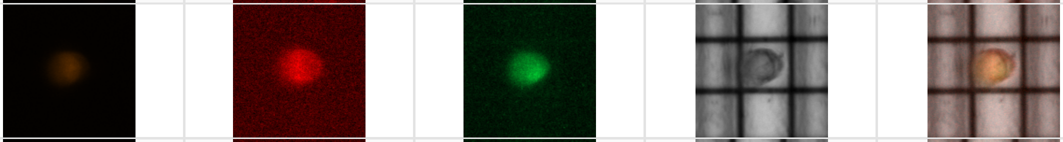					☑
449	GROUP 1	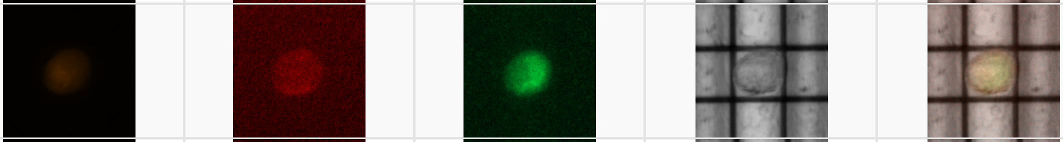					☑
802	GROUP 1	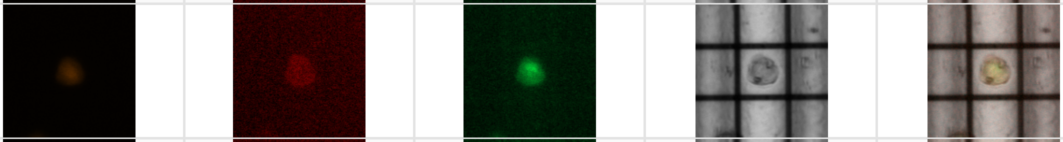					☑
815	GROUP 1	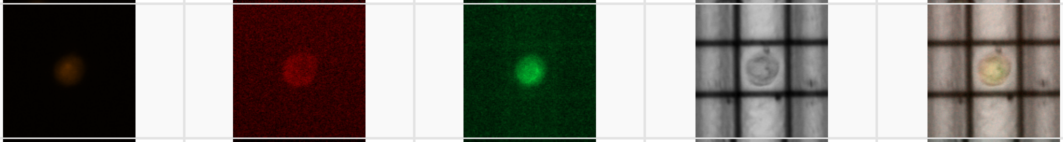					☑
1517	GROUP 1	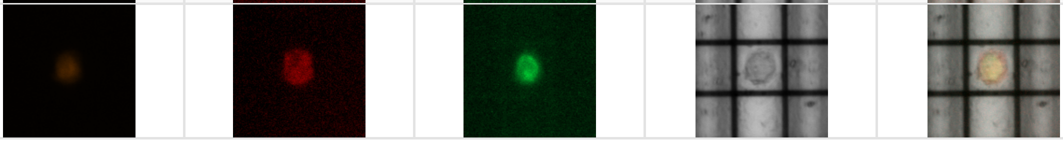					☑

id	Group	APC_0	PE_1	FITC_2	BRIGHTFIELD_3	APC_PE_FITC_ BRIGHTFIELD_4	
1851	GROUP 1	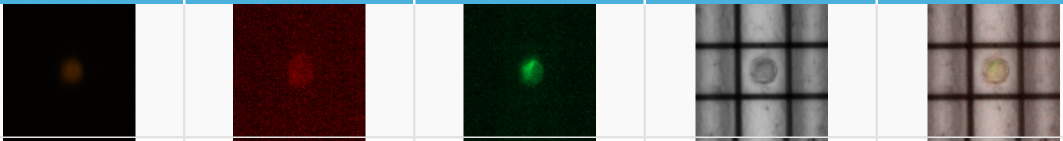					☑
1921	GROUP 1	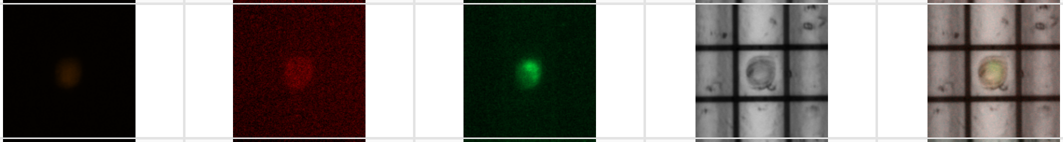					☑
2230	GROUP 1	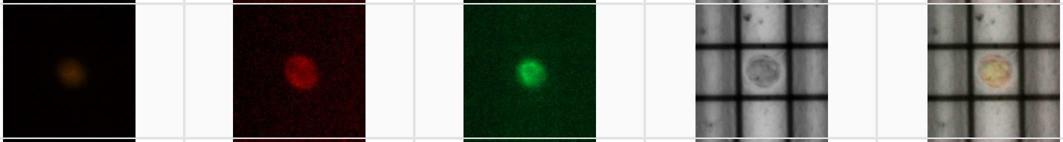					☑
2300	GROUP 1	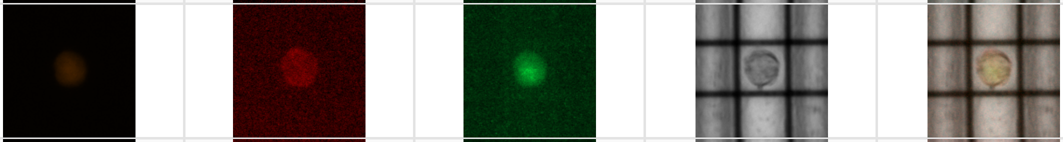					☑
2671	GROUP 1	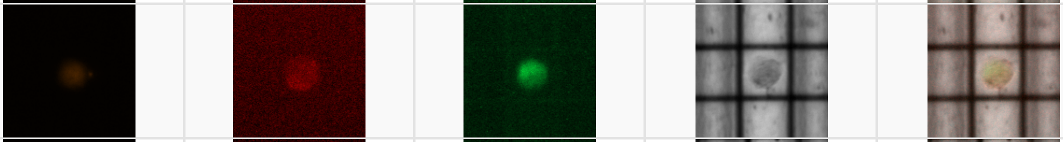					☑
2741	GROUP 1	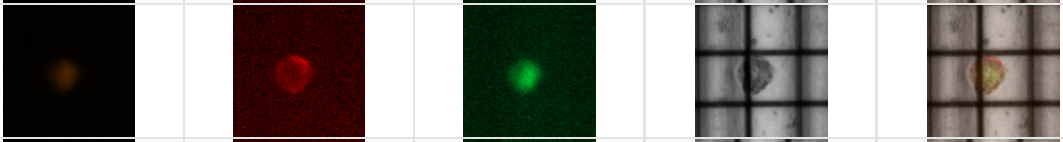					☑
3690	GROUP 1	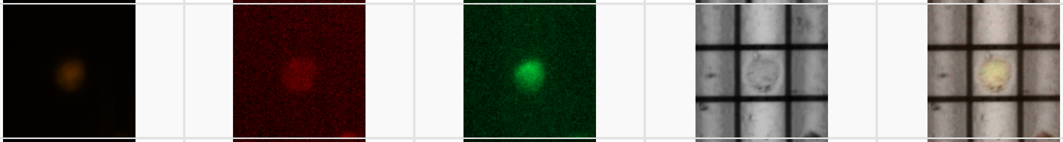					☑
3749	GROUP 1	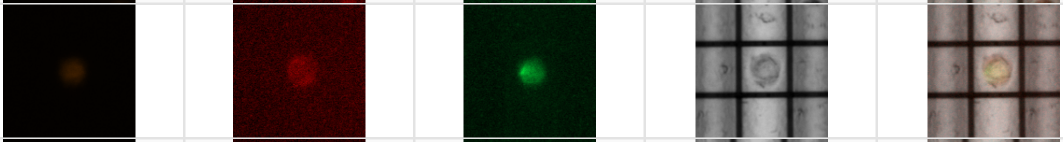					☑
3797	GROUP 1	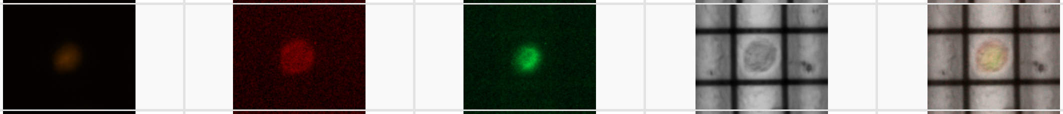					☑
4461	GROUP 1	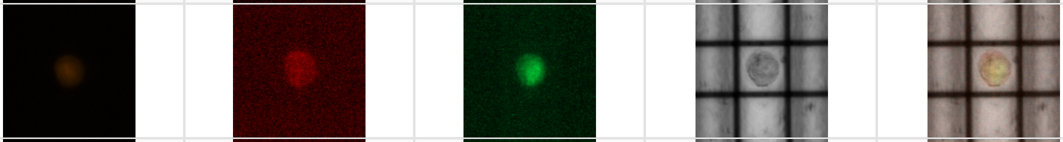					☑
5124	GROUP 1	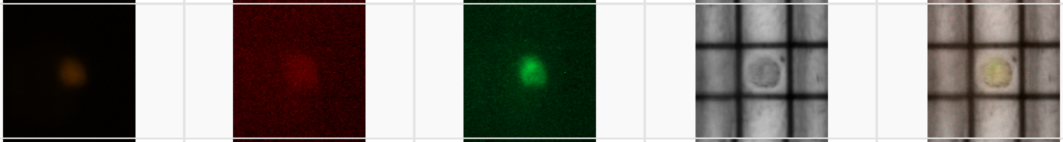					☑
5378	GROUP 1	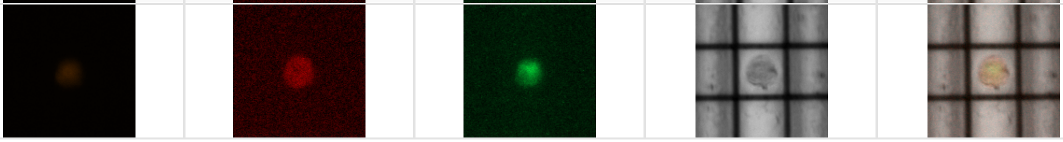					☑

id	Group	APC_0	PE_1	FITC_2	BRIGHTFIELD_3	APC_PE_FITC_ BRIGHTFIELD_4	
5667	GROUP 1	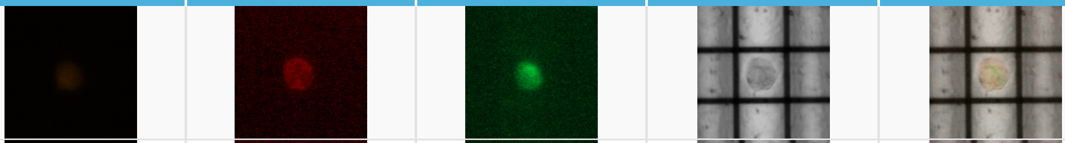					☑
5668	GROUP 1	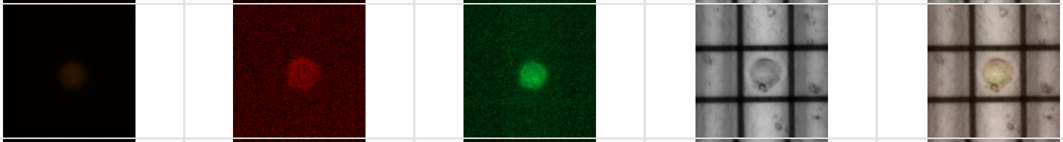					☑
5800	GROUP 1	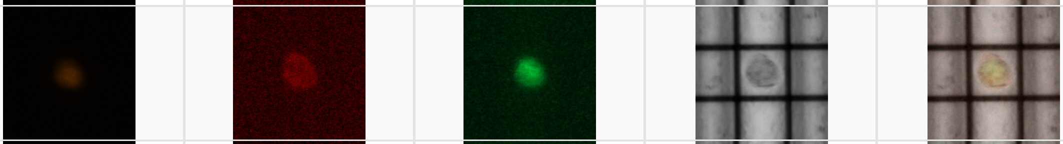					☑
6256	GROUP 1	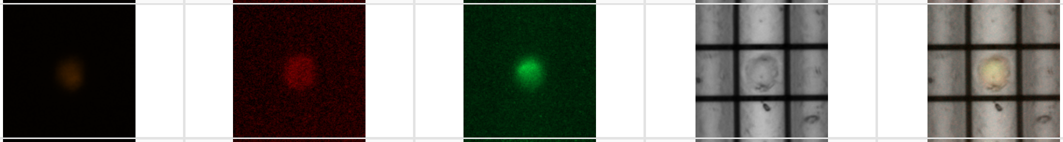					☑
6588	GROUP 1	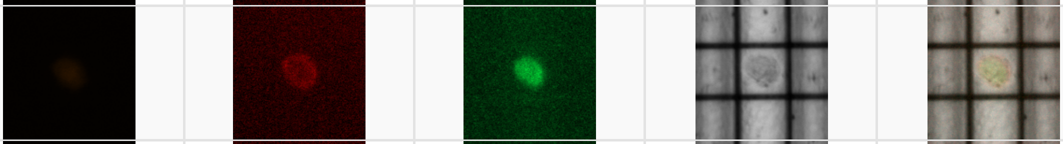					☑
6644	GROUP 1	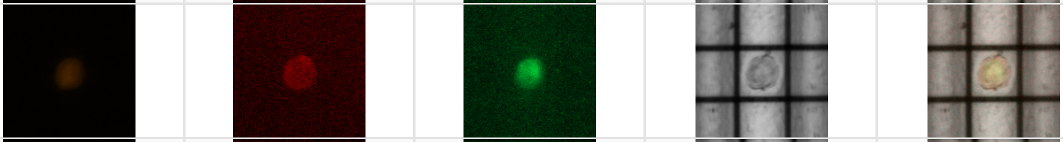					☑
6805	GROUP 1	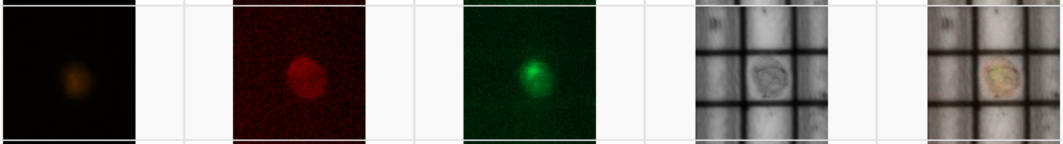					☑
7236	GROUP 1	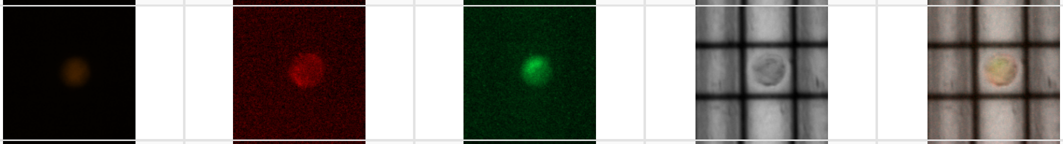					☑
6589	GROUP 1	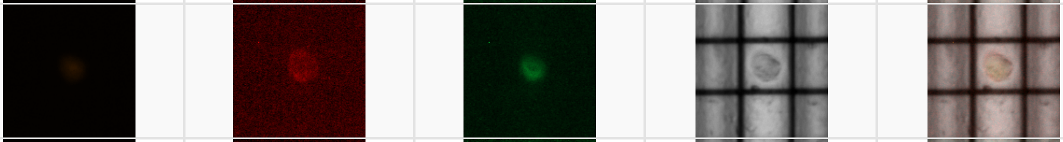					☑
6625	GROUP 1	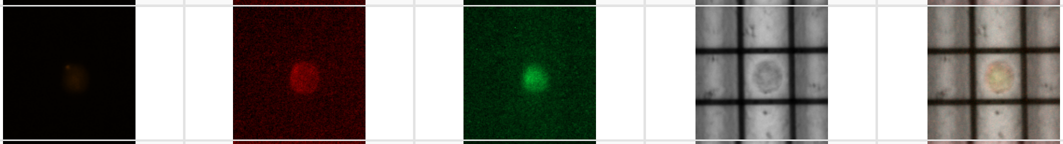					☑
787	GROUP 1	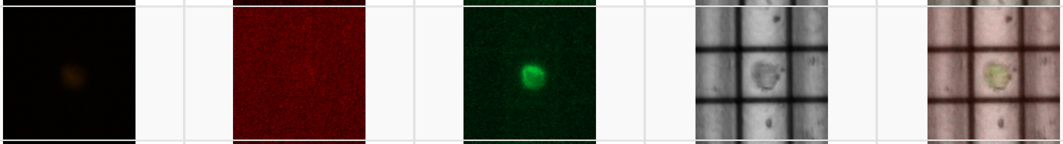					☑
5341	GROUP 1	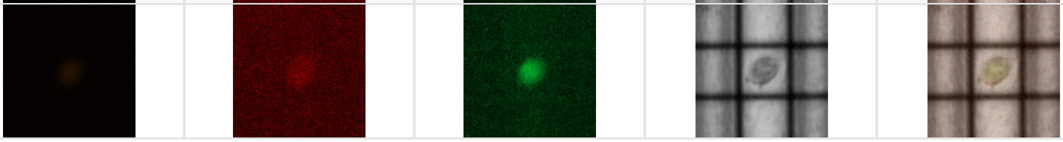					☑

id	Group	APC_0	PE_1	FITC_2	BRIGHTFIELD_3	APC_PE_FITC_ BRIGHTFIELD_4	
2497	GROUP 1	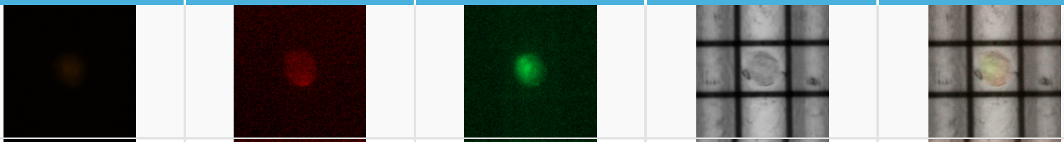					☑
6885	GROUP 1	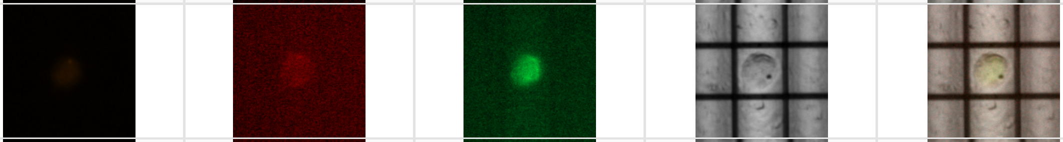					☑
1292	GROUP 1	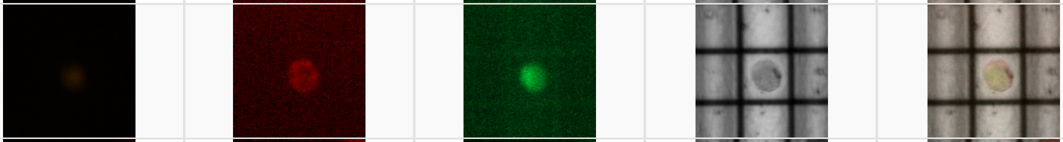					☑
2636	GROUP 1	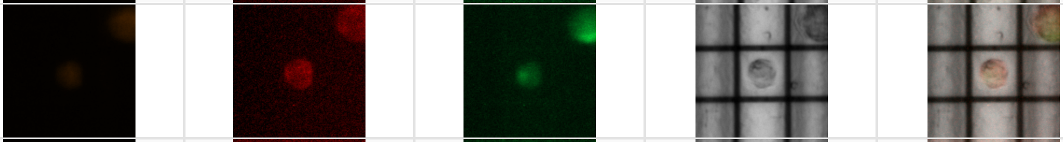					☑
2186	GROUP 1	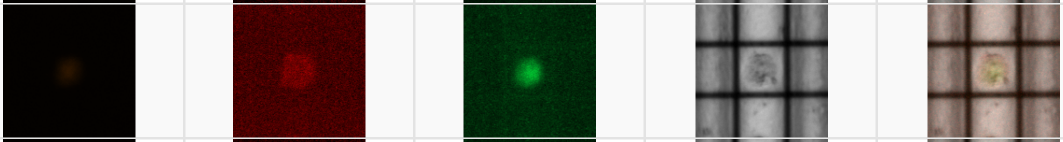					☑
6590	GROUP 1	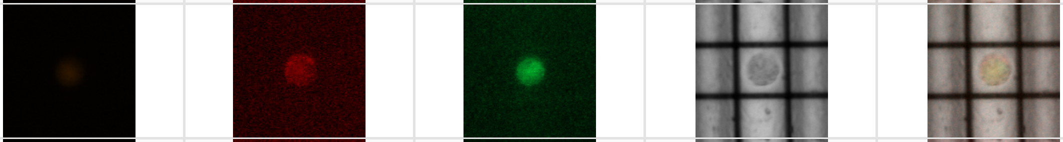					☑
4640	GROUP 1	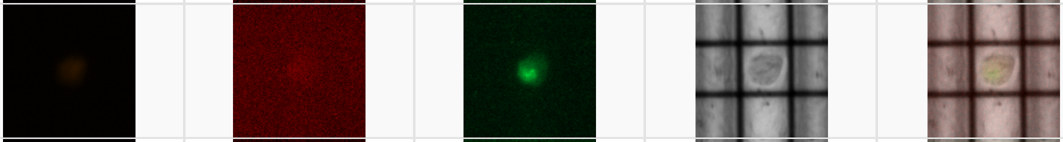					☑
6953	GROUP 1	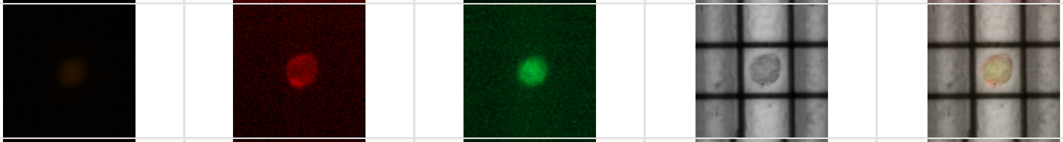					☑
2626	GROUP 1	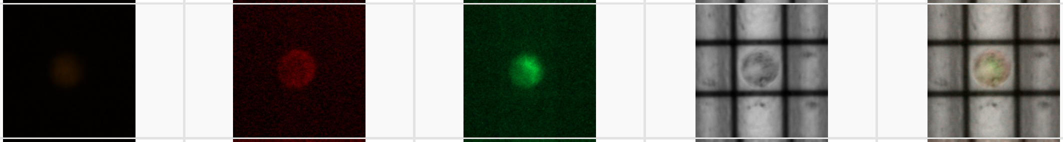					☑
7286	GROUP 1	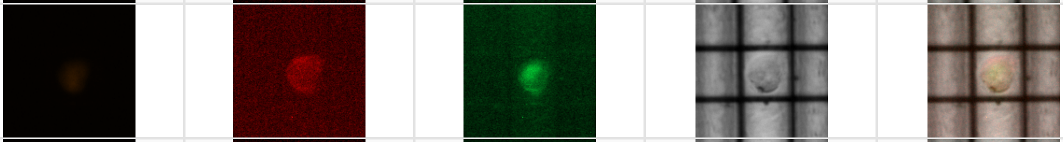					☑
5347	GROUP 1	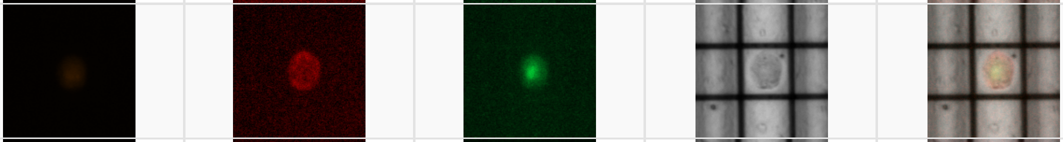					☑
6302	GROUP 1	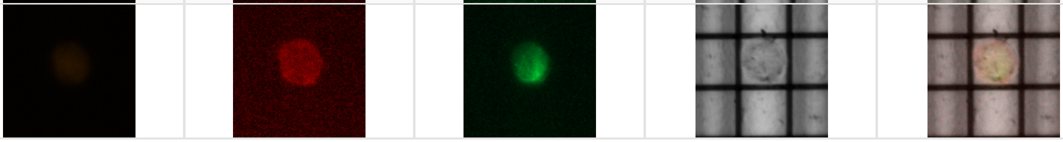					☑

id	Group	APC_0	PE_1	FITC_2	BRIGHTFIELD_3	APC_PE_FITC_ BRIGHTFIELD_4	
4440	GROUP 1	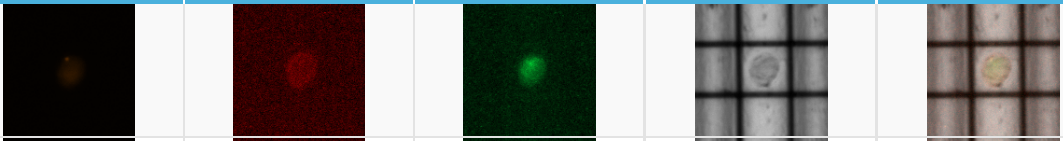					☑
6672	GROUP 1	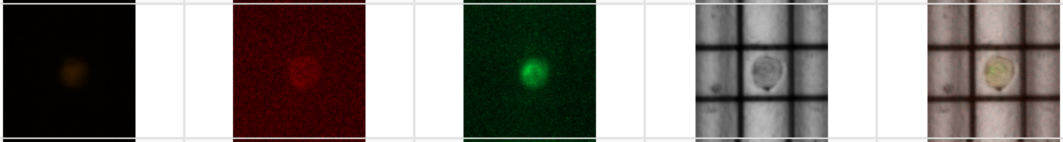					☑
3369	GROUP 1	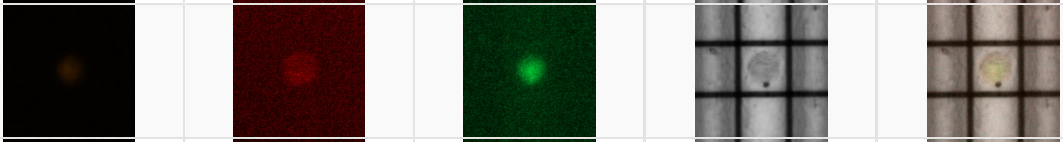					☑
6199	GROUP 1	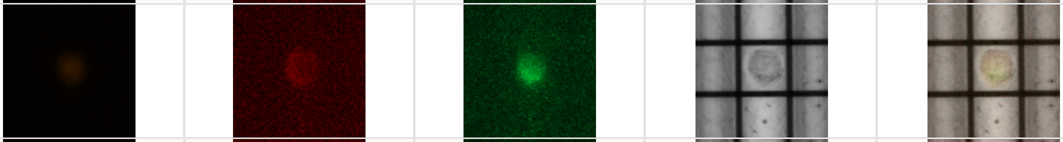					☑
7249	GROUP 1	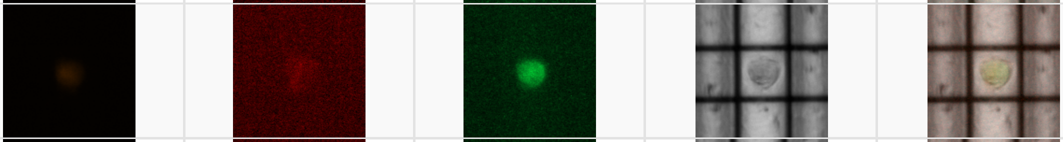					☑
6483	GROUP 1	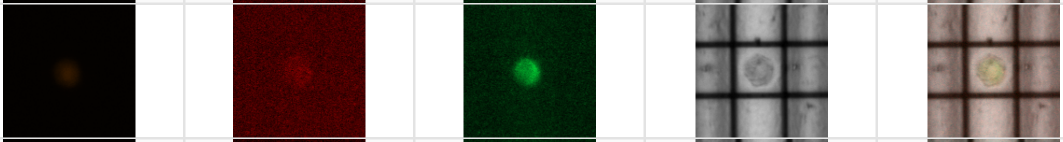					☑
7066	GROUP 1	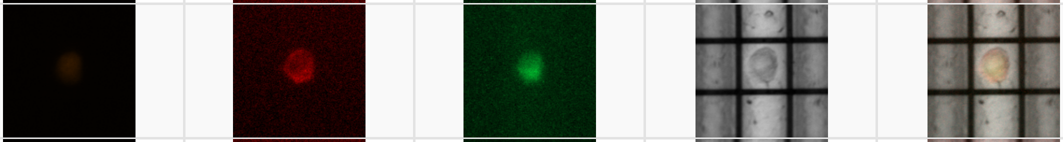					☑
5382	GROUP 1	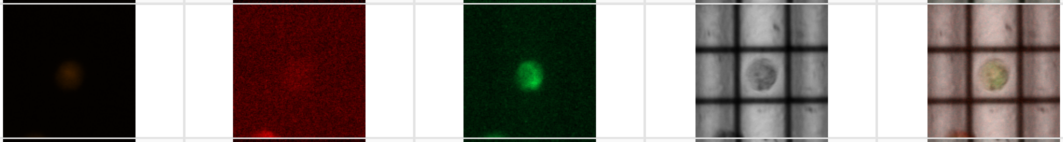					☑
7102	GROUP 1	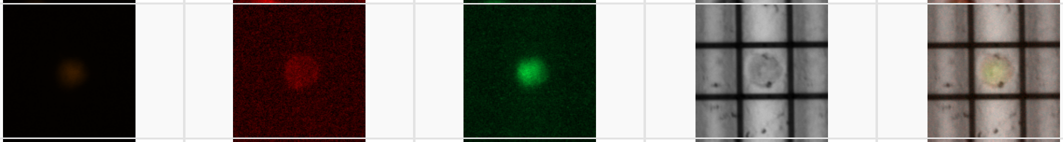					☑
6431	GROUP 1	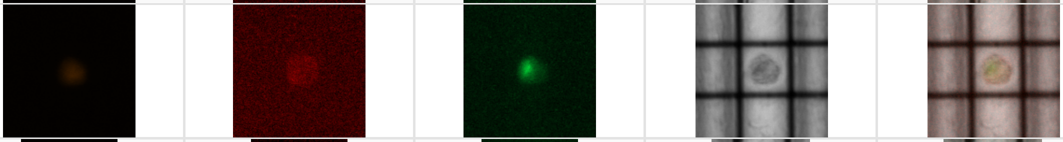					☑
6426	GROUP 1	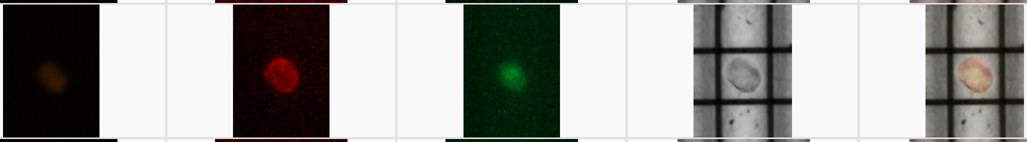					☑
5320	GROUP 1	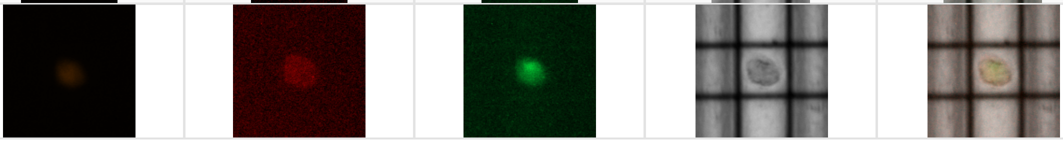					☑

id	Group	APC_0	PE_1	FITC_2	BRIGHTFIELD_3	APC_PE_FITC_ BRIGHTFIELD_4	
7267	GROUP 1	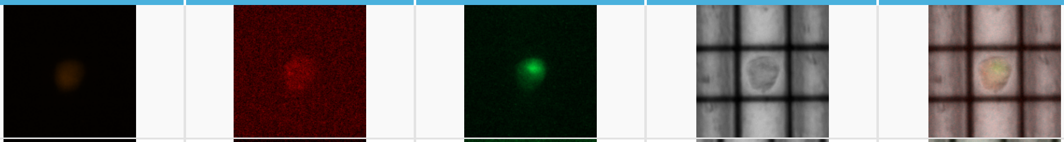					☑
832	GROUP 1	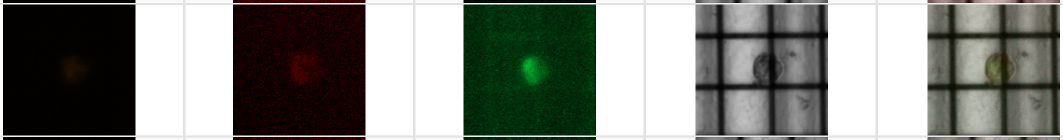					☑
4588	GROUP 1	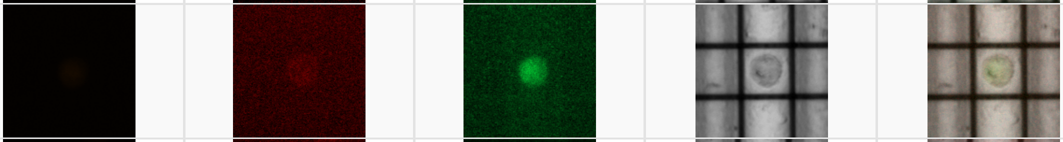					☑
5052	GROUP 1	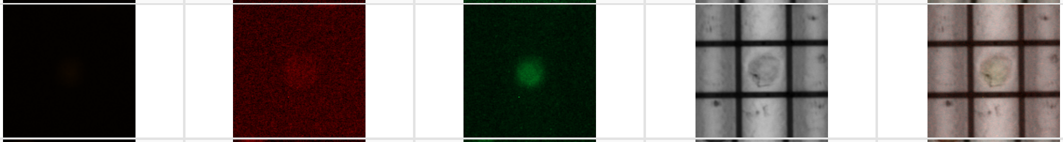					☑
5170	GROUP 1	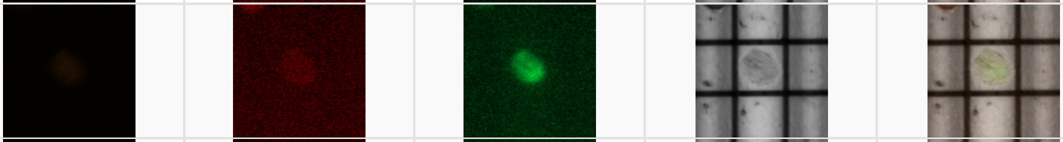					☑
5405	GROUP 1	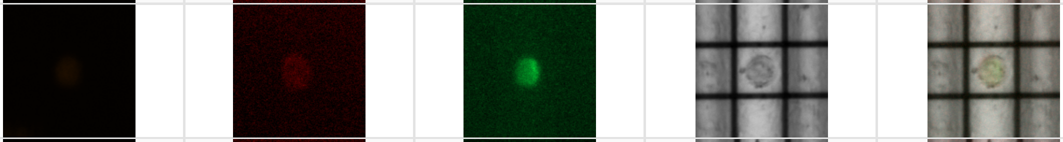					☑
5591	GROUP 1	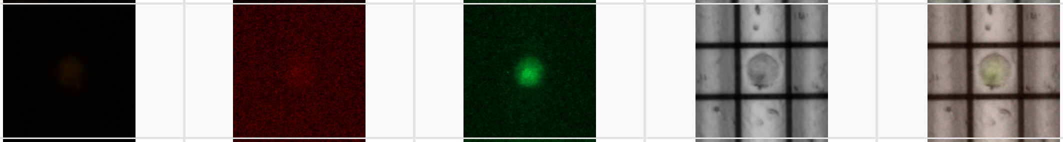					☑
5978	GROUP 1	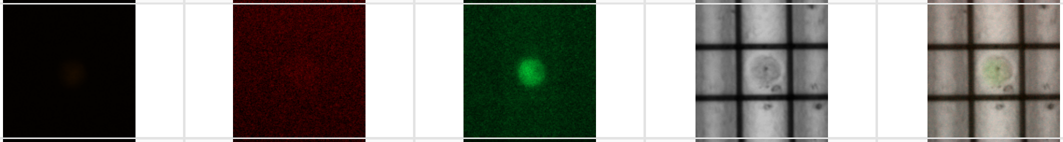					☑
6534	GROUP 1	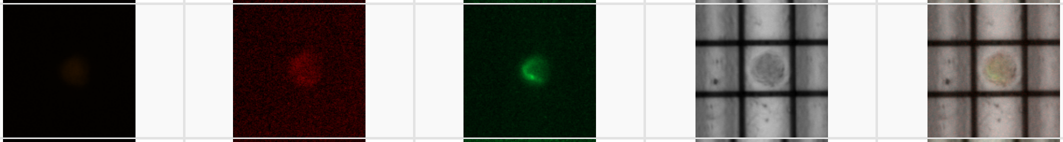					☑
6601	GROUP 1	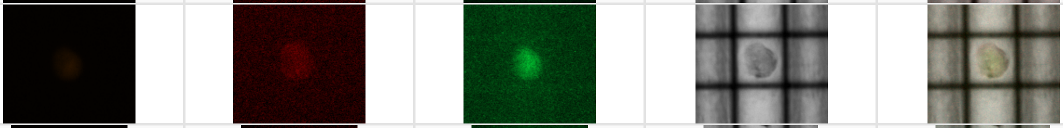					☑
6610	GROUP 1	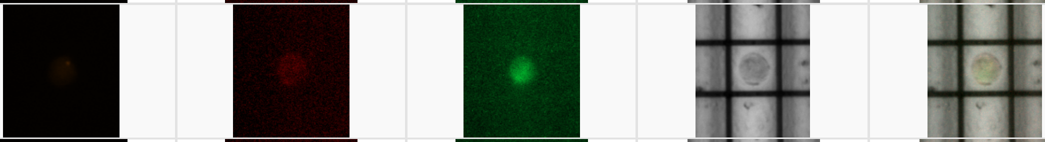					☑
6666	GROUP 1	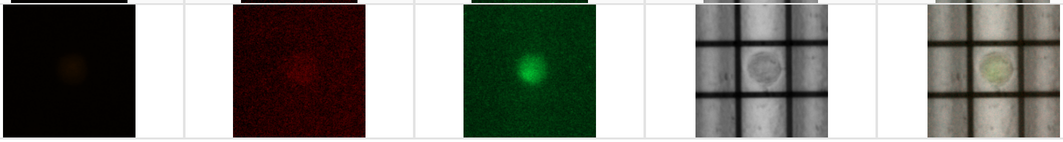					☑

Group 1

id	mean intensity apc	mean intensity brightfield	mean intensity fitc	mean intensity pe
74	238.06	2489.94	792.29	1137.26
708	429.05	3114.07	562.55	1108.49
1331	220.22	2288.09	1014.58	1072.60
2024	243.39	2345.18	830.03	979.04
2390	240.98	2526.15	634.64	1022.26
2523	305.80	2377.30	689.59	1048.91
2783	298.37	2223.55	1006.16	1105.84
2790	261.33	2278.10	1182.29	1154.06
2881	231.59	2701.98	962.21	999.29
3203	333.37	2336.38	760.35	992.63
3277	630.67	3043.87	700.52	1065.59
3486	274.75	2288.07	717.89	980.02
3573	277.82	2464.91	690.99	1123.99
3576	228.16	2566.18	679.28	940.28
3667	204.96	2734.25	643.46	1001.75
3732	200.52	2802.17	799.75	1223.04
3903	280.87	2385.57	838.74	1118.15
4832	203.92	2646.02	610.02	940.53
5624	231.74	2588.96	992.37	1114.81
6358	197.37	2908.55	621.41	989.11
6445	272.65	2629.10	917.71	1004.53
6950	219.02	2469.28	958.09	961.84
624	235.83	2291.19	843.59	1248.40
6617	186.43	2958.27	637.02	984.59
2319	185.43	2745.45	645.26	995.75
5837	175.27	2985.31	727.79	1032.14
757	174.92	2964.43	681.89	1056.41
5187	173.87	2531.19	703.55	955.30
5216	173.54	2954.71	712.14	964.34
3889	173.02	2678.83	596.31	983.41
4922	169.93	2527.22	606.82	1005.41
2077	169.61	2358.91	630.75	1056.55
449	153.21	2948.56	657.71	859.56
802	147.57	2720.88	570.61	826.14
815	147.28	3126.47	515.18	847.86
1517	150.25	2823.05	572.18	860.93
1851	141.33	2729.16	612.79	832.33
1921	117.60	2786.48	633.80	822.15
2230	141.33	2805.30	577.21	866.16
2300	145.18	2610.96	493.04	847.77
2671	138.32	2954.68	562.88	850.83
2741	147.00	2038.26	565.16	863.55
3690	154.66	3266.37	494.04	820.33
3749	129.93	3018.46	586.88	851.25
3797	150.31	3117.68	606.30	857.13
4461	142.89	2800.45	609.42	869.32
5124	151.14	2959.74	540.66	816.34
5378	125.97	2762.76	560.30	882.11
5667	115.37	3202.54	510.63	845.80
5668	114.08	3040.89	515.98	859.29
5800	149.00	2842.33	660.09	838.41
6256	131.13	3085.77	555.23	839.98
6588	102.79	3169.96	469.99	821.23
6644	149.14	3044.15	499.84	845.89
6805	141.50	2659.01	610.52	869.37
7236	141.52	2879.28	505.24	883.82
6589	100.14	3124.52	532.02	842.09

id	mean intensity apc	mean intensity brightfield	mean intensity fitc	mean intensity pe
6625	100.97	3144.46	452.68	870.87
787	101.06	2966.53	653.07	832.69
5341	101.38	2908.84	476.42	848.43
2497	101.80	3197.47	556.86	836.42
6885	103.04	2487.66	793.64	837.41
1292	104.23	3012.41	467.13	874.02
2636	104.61	2899.29	466.77	859.34
2186	105.67	3018.97	525.35	879.31
6590	106.62	3008.37	550.57	869.18
4640	107.35	3064.64	642.84	837.19
6953	107.67	2979.59	515.53	875.29
2626	108.68	2909.15	539.04	817.51
7286	112.20	2512.15	826.82	881.47
5347	112.41	2943.80	577.92	882.85
6302	113.68	2868.00	810.02	869.13
4440	114.24	3014.43	519.98	832.46
6672	115.19	2832.58	530.37	826.72
3369	115.41	3156.71	479.73	837.00
6199	116.23	2989.55	443.51	813.50
7249	116.94	3063.15	555.60	822.33
6483	117.02	3122.86	532.83	836.37
7066	117.83	3148.64	456.82	881.03
5382	119.51	2772.47	598.41	833.85
7102	122.00	2900.33	574.86	818.38
6431	124.44	2839.85	590.11	835.25
6426	128.20	3100.14	474.54	872.93
5320	127.96	2712.36	535.74	831.07
7267	127.67	2990.58	751.07	850.29
832	93.87	1826.67	455.38	749.16
4588	77.80	3124.97	437.85	790.31
5052	70.31	3057.53	485.65	794.86
5170	97.54	2902.39	526.26	782.81
5405	91.33	3010.66	467.16	750.26
5591	94.03	2895.49	588.12	777.38
5978	79.98	3273.53	486.43	755.23
6534	92.82	2756.72	586.23	790.97
6601	98.32	2958.19	421.50	787.40
6610	89.94	2970.93	463.55	749.46
6666	83.26	3060.28	473.91	765.97
