# Supplementary material for: 3D genome organization in the epithelial-mesenchymal transition spectrum
Source: Genome Biol. 2022 May 30;23:121. doi: 10.1186/s13059-022-02687-x (PMC9150291; doi:10.1186/s13059-022-02687-x)
Supplement: Supplementary file 9 — Additional file 9. DEPArray™ NxT system generated reports for single-cell isolation. The reports contain the parameters used for immunofluorescence and the images of single cells captured by the system. [file 13059_2022_2687_MOESM9_ESM.zip › PEO1_HEYA8_DepArray_Report.pdf]

DEPArray™ NxT Run Report not for IVD use

System information

**DEPArray™ NxT System Serial Number:**	D03-001-0069
**DEPArray™ NxT System Software Release:**	3.5.1.8.1
**DEPArray™ NxT System intended for:**	Research Use Only

Run information

**Selected application:**	CTC-RUO-FIXED
**Run ID:**	20092019
**Run mode:**	Post processing
**DEPArray™ NxT Cartridge:**	950002835
**DEPArray™ Buffer:**	
**User ID:**	NCCS
**Date:**	2019 / 09 / 20

Scan settings	Chip scan				Image analysis		
	Exposure (ms)	Camera Gain	Lamp intensity (%)	Offset (um)	Detection	Analysis	Duplicate removal
**CHIP SCAN 1**							
FITC	1000	1X	100%	35	Faint Signal	Enable	
PE	1000	1X	100%	40	Faint Signal	Enable	☑
BRIGHTFIELD	3	1X	5%	26	Faint Signal	Enable	

Data Display

Group 1

id	Group	PE_0	FITC_1	BRIGHTFIELD_2	
3357	GROUP 1	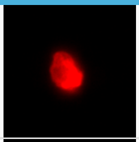	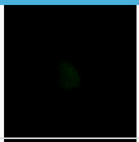	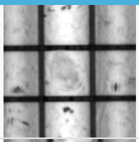	☑
5844	GROUP 1	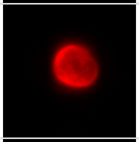	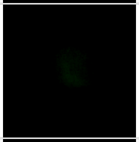	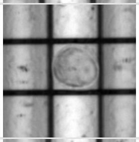	☑
5179	GROUP 1	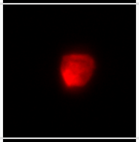	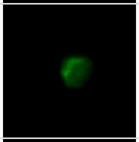	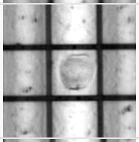	☑
974	GROUP 1	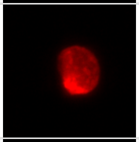	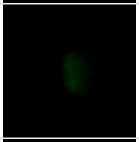	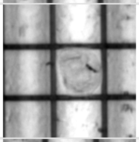	☑
3189	GROUP 1	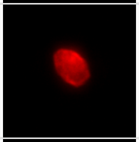	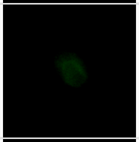	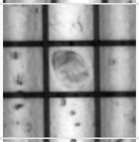	☑
213	GROUP 1	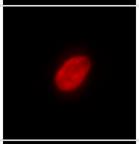	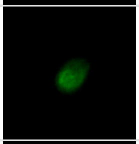	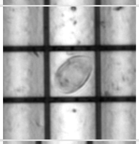	☑
488	GROUP 1	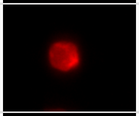	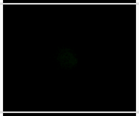	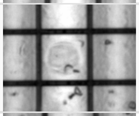	☑
7561	GROUP 1	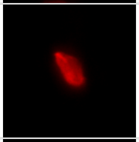	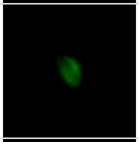	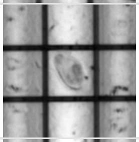	☑
4402	GROUP 1	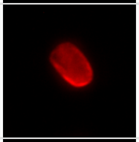	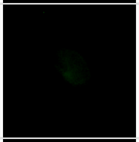	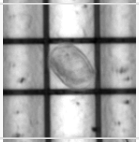	☑
1192	GROUP 1	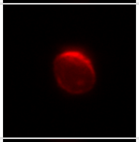	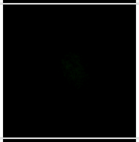	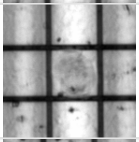	☑
5581	GROUP 1	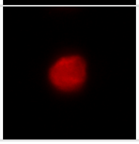	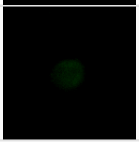	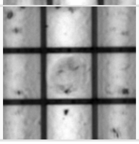	☑

id	Group	PE_0	FITC_1	BRIGHTFIELD_2	
556	GROUP 1	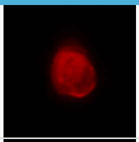	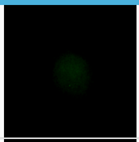	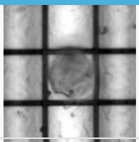	☑
8253	GROUP 1	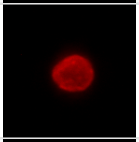	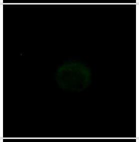	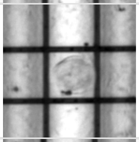	☑
249	GROUP 1	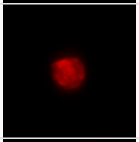	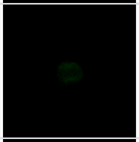	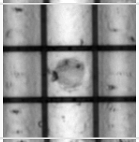	☑
4698	GROUP 1	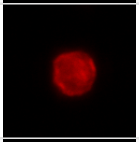	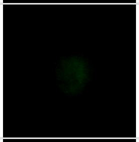	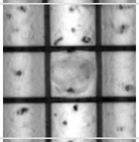	☑
7929	GROUP 1	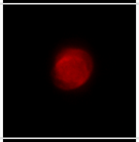	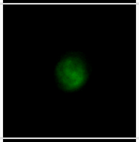	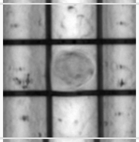	☑
6411	GROUP 1	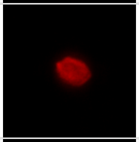	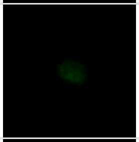	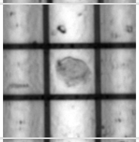	☑
956	GROUP 1	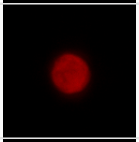	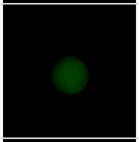	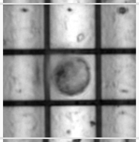	☑
1537	GROUP 1	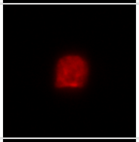	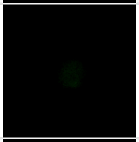	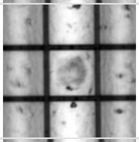	☑
761	GROUP 1	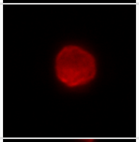	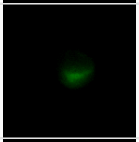	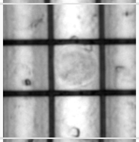	☑
7556	GROUP 1	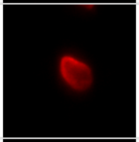	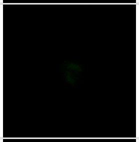	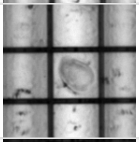	☑
5429	GROUP 1	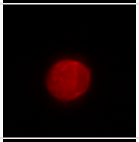	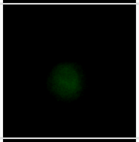	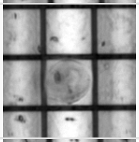	☑
7422	GROUP 1	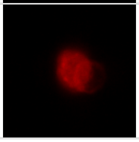	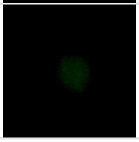	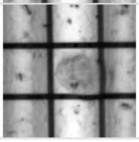	☑

id	Group	PE_0	FITC_1	BRIGHTFIELD_2	
350	GROUP 1	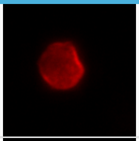	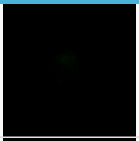	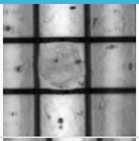	☑
7578	GROUP 1	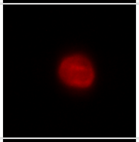	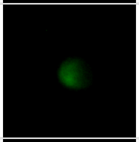	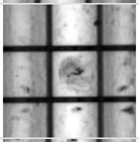	☑
2430	GROUP 1	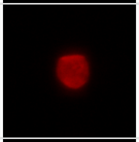	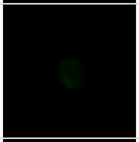	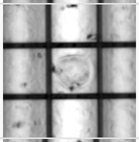	☑
3369	GROUP 1	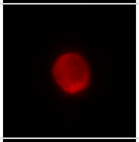	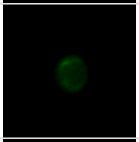	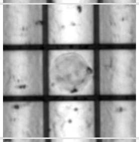	☑
232	GROUP 1	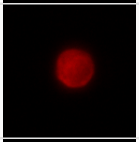	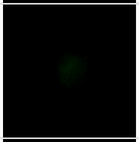	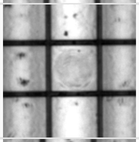	☑
7288	GROUP 1	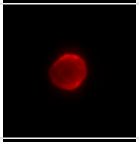	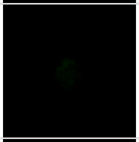	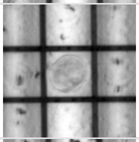	☑
3631	GROUP 1	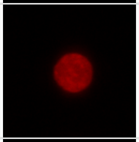	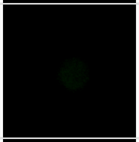	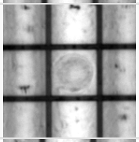	☑
6247	GROUP 1	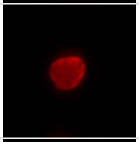	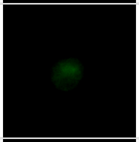	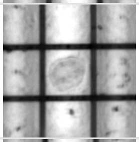	☑
5603	GROUP 1	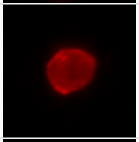	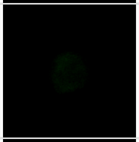	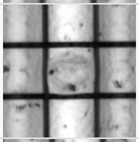	☑
5032	GROUP 1	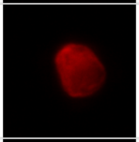	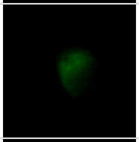	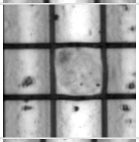	☑
9050	GROUP 1	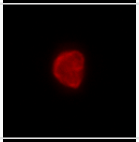	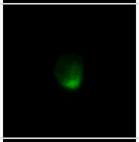	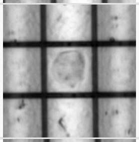	☑
389	GROUP 1	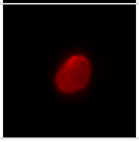	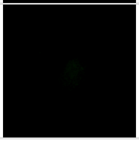	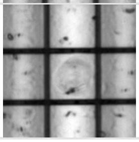	☑

id	Group	PE_0	FITC_1	BRIGHTFIELD_2	
7234	GROUP 1	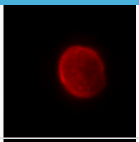	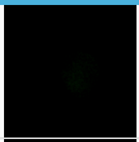	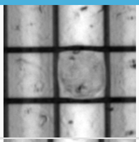	☑
208	GROUP 1	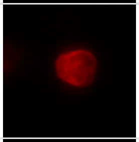	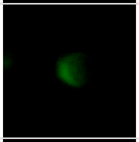	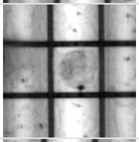	☑
1712	GROUP 1	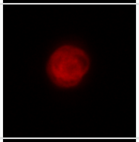	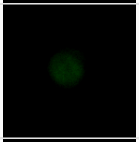	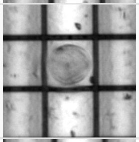	☑
5804	GROUP 1	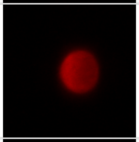	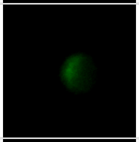	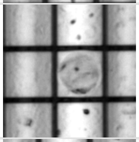	☑
387	GROUP 1	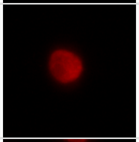	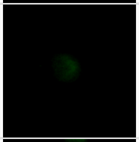	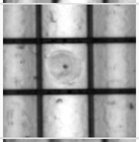	☑
5684	GROUP 1	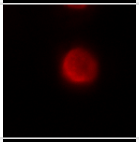	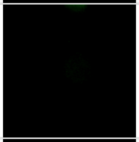	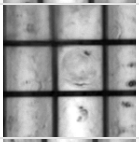	☑
3925	GROUP 1	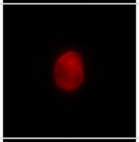	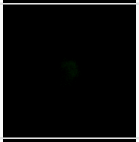	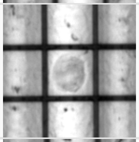	☑
8033	GROUP 1	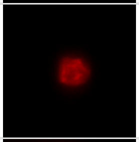	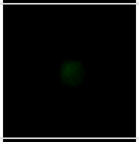	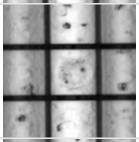	☑
6186	GROUP 1	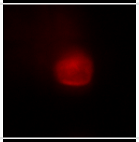	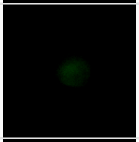	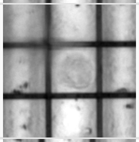	☑
4346	GROUP 1	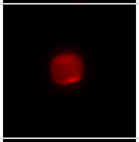	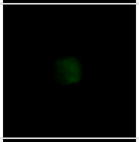	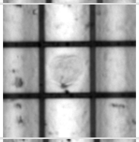	☑
1040	GROUP 1	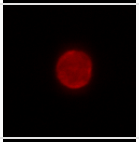	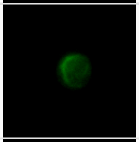	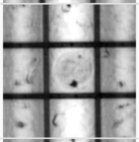	☑
428	GROUP 1	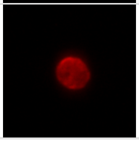	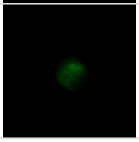	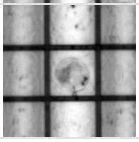	☑

id	Group	PE_0	FITC_1	BRIGHTFIELD_2	
523	GROUP 1	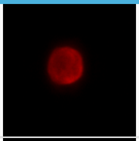	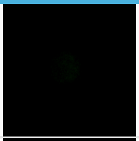	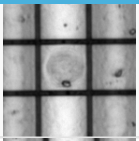	☑
2320	GROUP 1	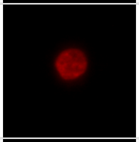	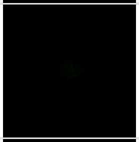	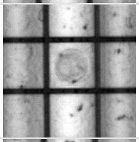	☑
8357	GROUP 1	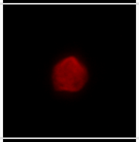	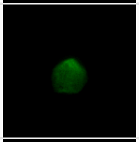	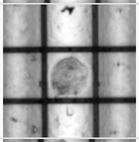	☑
8350	GROUP 1	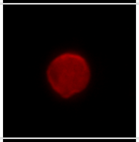	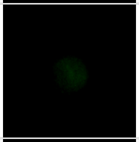	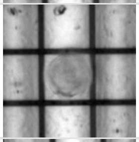	☑
3977	GROUP 1	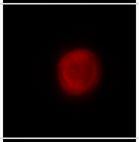	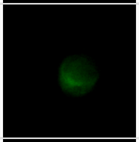	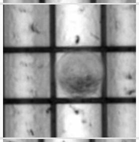	☑
789	GROUP 1	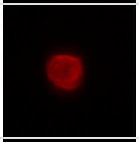	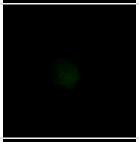	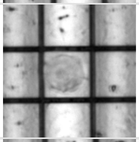	☑
3638	GROUP 1	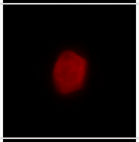	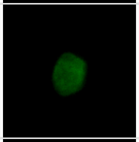	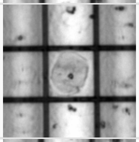	☑
1351	GROUP 1	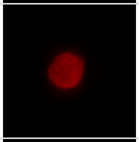	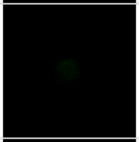	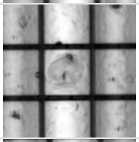	☑
1232	GROUP 1	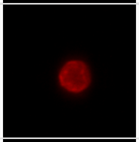	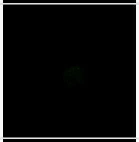	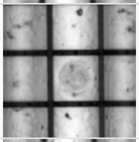	☑
7795	GROUP 1	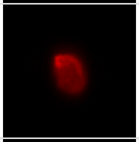	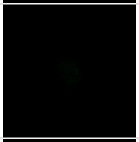	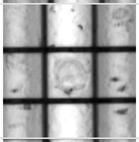	☑
2227	GROUP 1	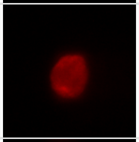	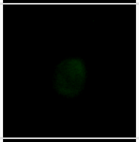	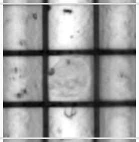	☑
5731	GROUP 1	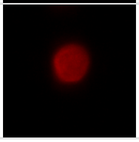	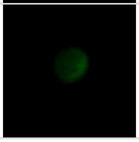	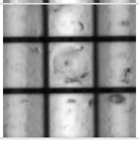	☑

id	Group	PE_0	FITC_1	BRIGHTFIELD_2	
6731	GROUP 1	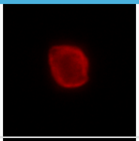	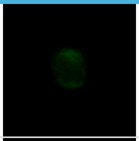	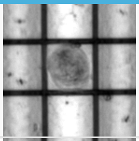	☑
9129	GROUP 1	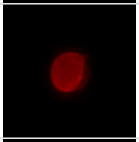	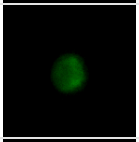	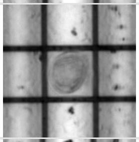	☑
5000	GROUP 1	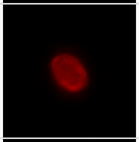	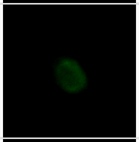	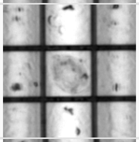	☑
5369	GROUP 1	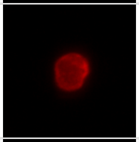	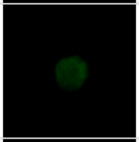	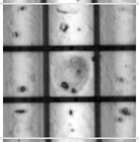	☑
3127	GROUP 1	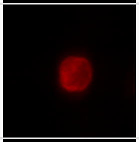	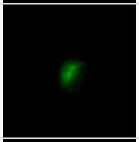	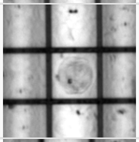	☑
7938	GROUP 1	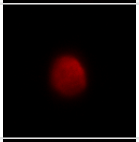	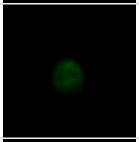	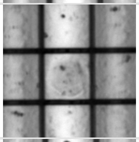	☑
4483	GROUP 1	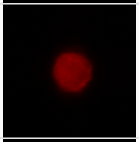	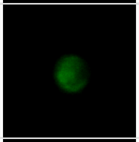	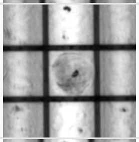	☑
7036	GROUP 1	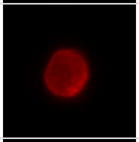	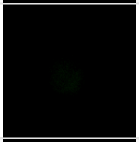	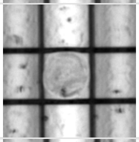	☑
4599	GROUP 1	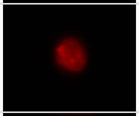	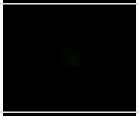	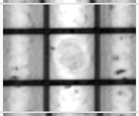	☑
5569	GROUP 1	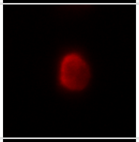	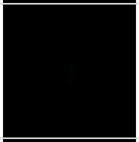	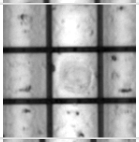	☑
6370	GROUP 1	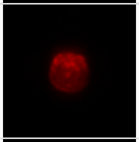	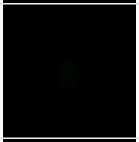	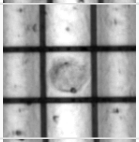	☑
63	GROUP 1	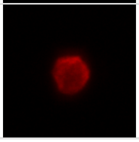	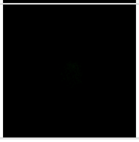	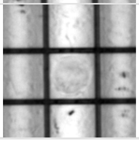	☑

id	Group	PE_0	FITC_1	BRIGHTFIELD_2	
6884	GROUP 1	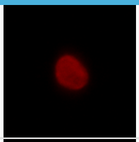	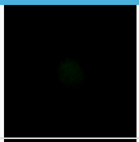	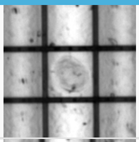	☑
4300	GROUP 1	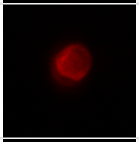	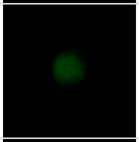	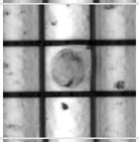	☑
2393	GROUP 1	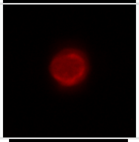	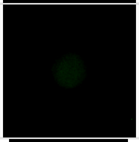	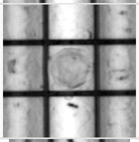	☑
8848	GROUP 1	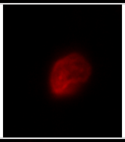	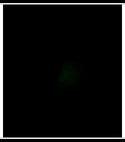	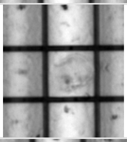	☑
8074	GROUP 1	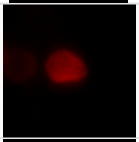	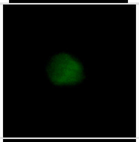	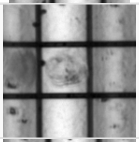	☑
7876	GROUP 1	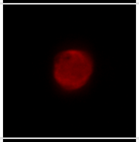	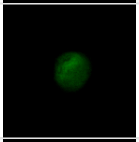	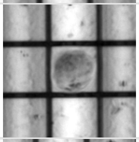	☑
3541	GROUP 1	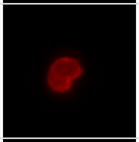	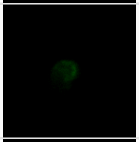	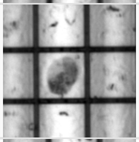	☑
829	GROUP 1	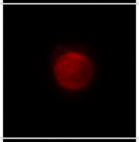	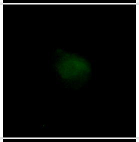	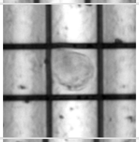	☑
1275	GROUP 1	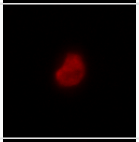	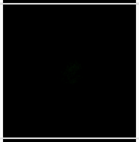	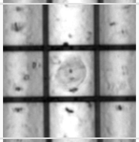	☑
7432	GROUP 1	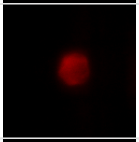	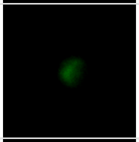	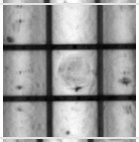	☑
6585	GROUP 1	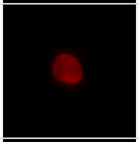	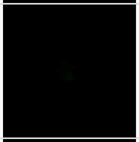	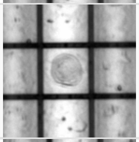	☑
7558	GROUP 1	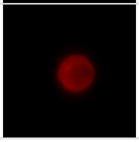	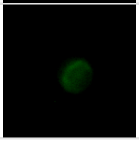	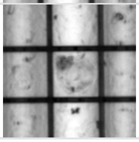	☑

id	Group	PE_0	FITC_1	BRIGHTFIELD_2	
8400	GROUP 1	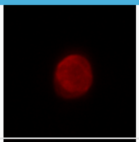	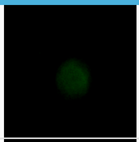	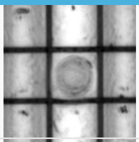	☑
7462	GROUP 1	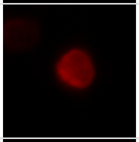	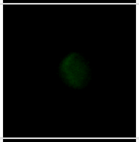	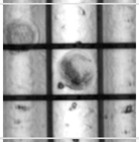	☑
8936	GROUP 1	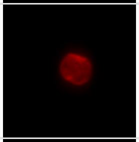	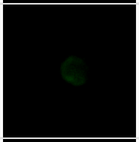	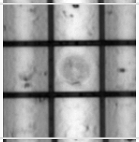	☑
6294	GROUP 1	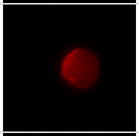	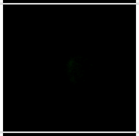	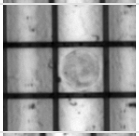	☑
3011	GROUP 1	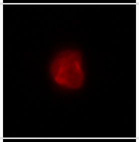	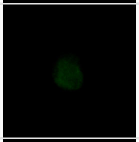	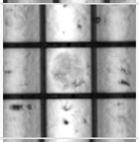	☑
4256	GROUP 1	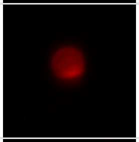	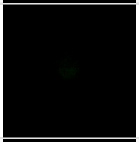	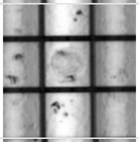	☑
6135	GROUP 1	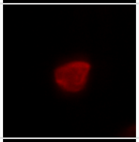	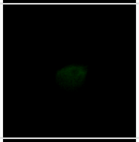	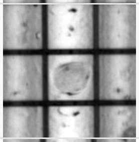	☑
3014	GROUP 1	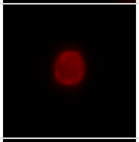	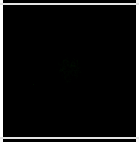	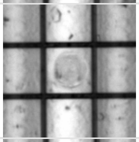	☑
1289	GROUP 1	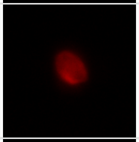	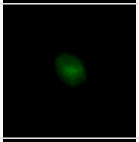	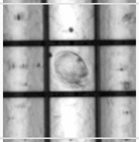	☑
1216	GROUP 1	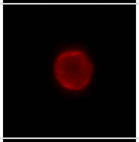	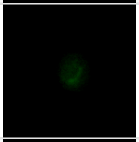	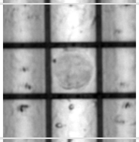	☑
3415	GROUP 1	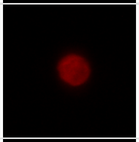	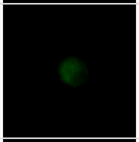	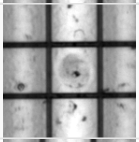	☑
1868	GROUP 1	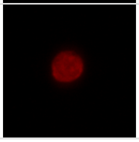	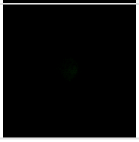	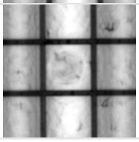	☑

id	Group	PE_0	FITC_1	BRIGHTFIELD_2	
3884	GROUP 1	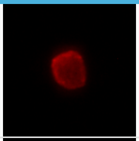	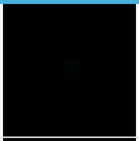	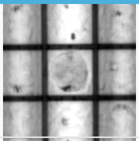	☑
3356	GROUP 1	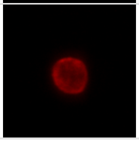	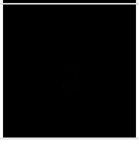	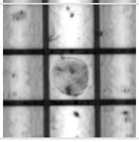	☑

Group 1

id	signal intensity fitc	signal intensity pe
3357	37.30	655.48
5844	37.49	542.39
5179	97.25	502.63
974	49.56	488.44
3189	51.33	460.99
213	106.37	433.90
488	30.22	425.73
7561	96.58	419.62
4402	36.33	407.62
1192	29.49	396.66
5581	47.51	395.90
556	48.27	381.86
8253	39.57	378.12
249	42.63	377.90
4698	43.15	377.12
7929	107.97	371.37
6411	47.64	369.48
956	82.00	369.01
1537	32.83	356.38
761	78.78	356.17
7556	33.96	355.51
5429	61.61	355.23
7422	52.85	354.46
350	29.22	350.78
7578	87.27	348.52
2430	37.16	348.50
3369	60.74	348.48
232	36.05	346.82
7288	32.19	345.11
3631	33.58	344.83
6247	57.58	344.16
5603	36.93	342.74
5032	81.22	342.80
9050	97.56	342.61
389	29.25	340.38
7234	32.04	340.14
208	85.46	337.06
1712	56.86	335.84
5804	76.44	333.27
387	45.46	332.09
5684	26.61	330.76
3925	28.93	329.55

id	signal intensity fitc	signal intensity pe
8033	44.70	327.98
6186	45.33	327.10
4346	53.51	326.75
1040	83.44	322.43
428	67.41	320.92
523	29.42	320.88
2320	28.20	319.13
8357	96.02	319.12
8350	45.96	318.65
3977	79.70	318.57
789	40.74	317.99
3638	99.24	317.32
1351	33.12	316.89
1232	28.96	315.93
7795	28.49	312.81
2227	44.41	310.18
5731	66.45	309.27
6731	51.62	308.44
9129	100.03	308.00
5000	64.10	307.91
5369	65.95	307.60
3127	102.73	307.24
7938	69.73	303.01
4483	98.09	302.44
7036	32.53	302.17
4599	28.42	301.14
5569	22.47	300.44
6370	29.28	300.36
63	24.41	298.64
6884	33.65	296.91
4300	74.03	296.37
2393	41.23	295.61
8848	34.44	295.53
8074	90.75	295.16
7876	94.01	294.10
3541	48.25	292.39
829	63.56	289.55
1275	23.26	287.24
7432	72.10	286.20
6585	26.62	284.96
7558	72.59	283.47
8400	60.24	282.56
7462	54.74	281.03
8936	45.21	280.79
6294	26.52	279.06
3011	55.07	278.89
4256	27.97	278.81
6135	45.21	277.74
3014	24.95	276.24
1289	84.56	274.56
1216	54.42	274.25
3415	60.85	273.90
1868	25.64	273.27
3884	27.35	270.76
3356	23.18	270.71

Data Display

Group 2

id	Group	PE_0	FITC_1	BRIGHTFIELD_2	
4859	GROUP 2	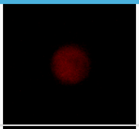	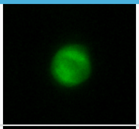	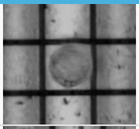	☑
3004	GROUP 2	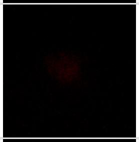	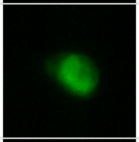	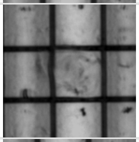	☑
4714	GROUP 2	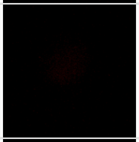	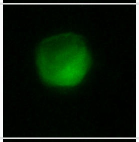	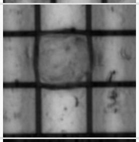	☑
2283	GROUP 2	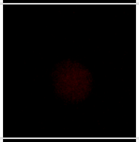	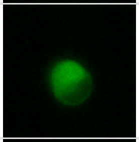	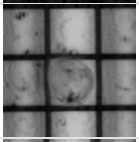	☑
2131	GROUP 2	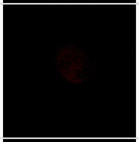	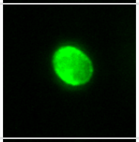	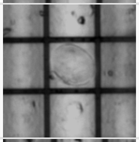	☑
4874	GROUP 2	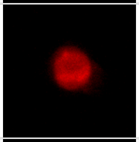	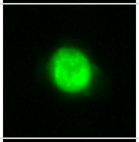	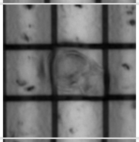	☑
4599	GROUP 2	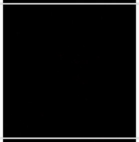	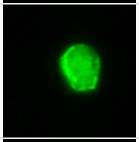	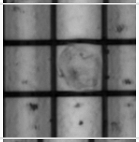	☑
2122	GROUP 2	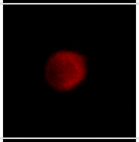	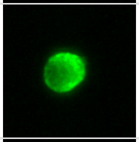	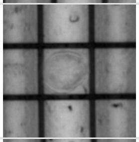	☑
7312	GROUP 2	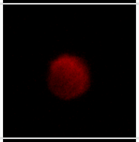	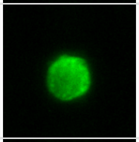	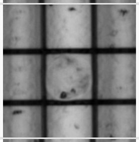	☑
2657	GROUP 2	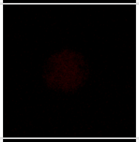	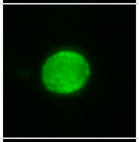	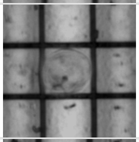	☑
6735	GROUP 2	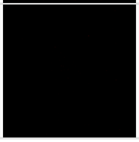	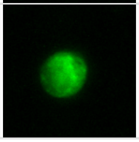	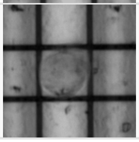	☑

id	Group	PE_0	FITC_1	BRIGHTFIELD_2	
7522	GROUP 2	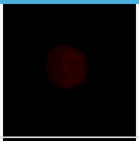	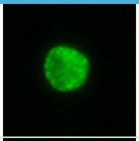	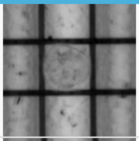	☑
7030	GROUP 2	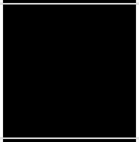	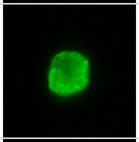	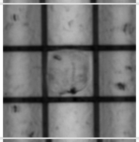	☑
6038	GROUP 2	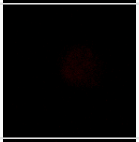	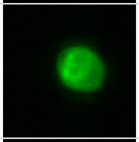	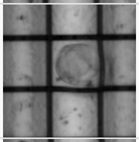	☑
5964	GROUP 2	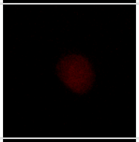	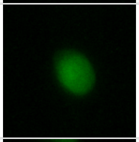	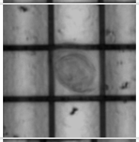	☑
8012	GROUP 2	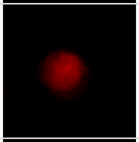	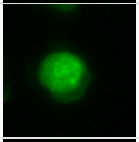	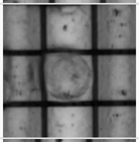	☑
3640	GROUP 2	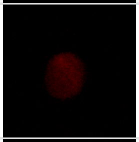	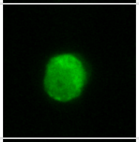	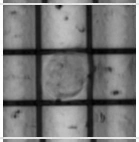	☑
6397	GROUP 2	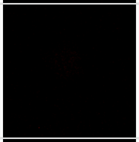	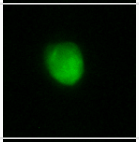	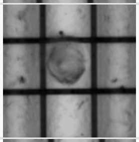	☑
6100	GROUP 2	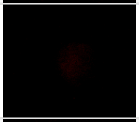	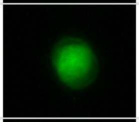	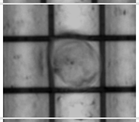	☑
2433	GROUP 2	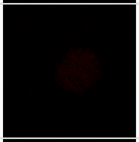	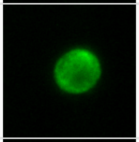	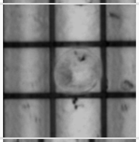	☑
3002	GROUP 2	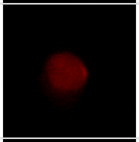	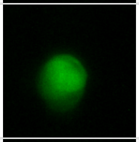	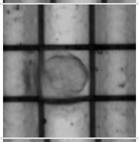	☑
8687	GROUP 2	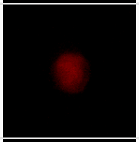	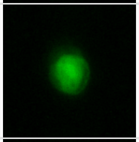	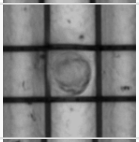	☑
9041	GROUP 2	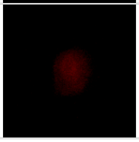	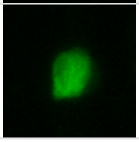	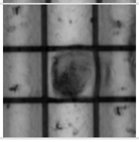	☑

id	Group	PE_0	FITC_1	BRIGHTFIELD_2	
980	GROUP 2	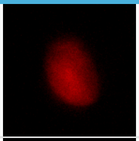	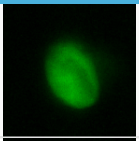	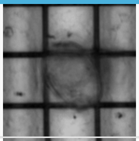	☑
5554	GROUP 2	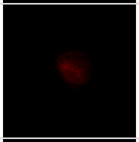	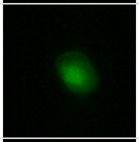	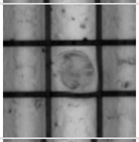	☑
7306	GROUP 2	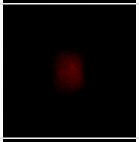	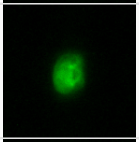	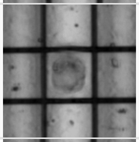	☑
7398	GROUP 2	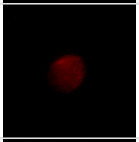	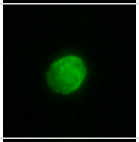	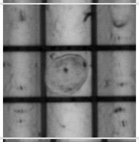	☑
3555	GROUP 2	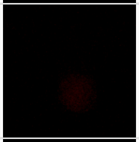	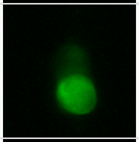	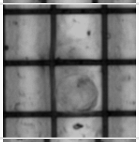	☑
4315	GROUP 2	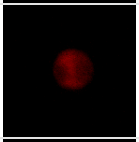	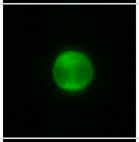	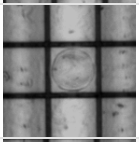	☑
5852	GROUP 2	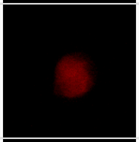	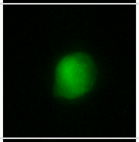	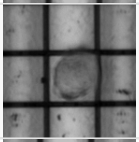	☑
7789	GROUP 2	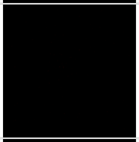	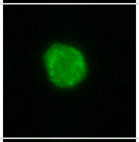	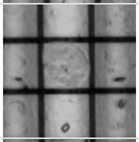	☑
5862	GROUP 2	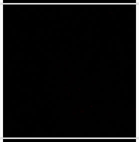	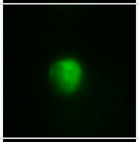	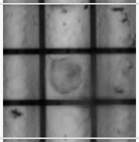	☑
9031	GROUP 2	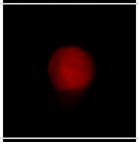	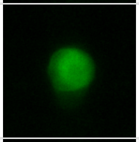	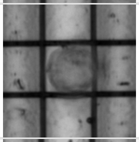	☑
6002	GROUP 2	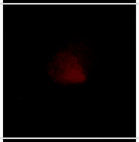	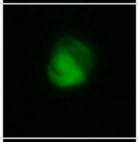	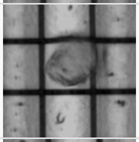	☑
5494	GROUP 2	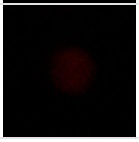	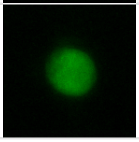	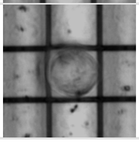	☑

id	Group	PE_0	FITC_1	BRIGHTFIELD_2	
3321	GROUP 2	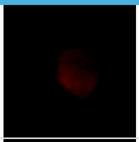	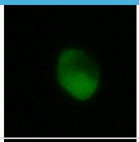	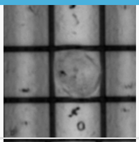	☑
1394	GROUP 2	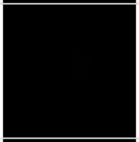	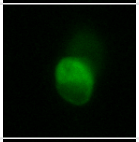	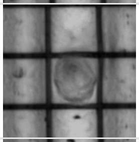	☑
655	GROUP 2	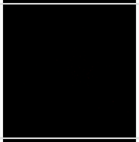	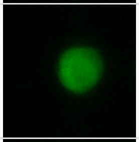	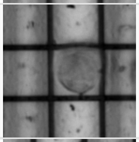	☑
8752	GROUP 2	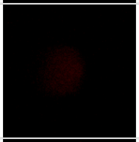	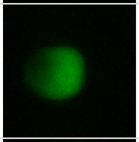	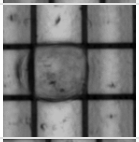	☑
10104	GROUP 2	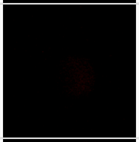	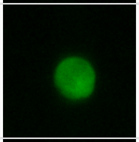	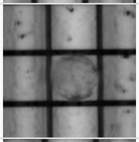	☑
1067	GROUP 2	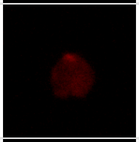	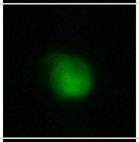	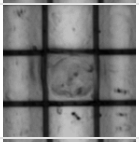	☑
6369	GROUP 2	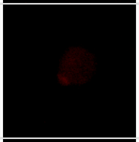	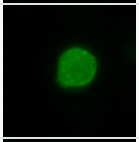	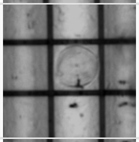	☑
2045	GROUP 2	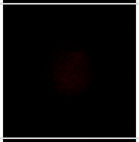	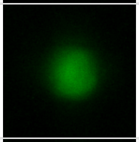	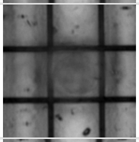	☑
3880	GROUP 2	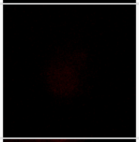	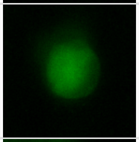	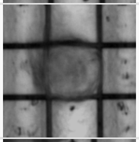	☑
7583	GROUP 2	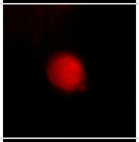	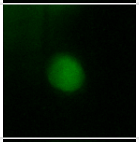	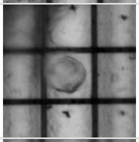	☑
5622	GROUP 2	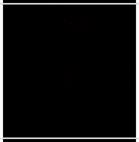	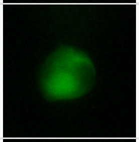	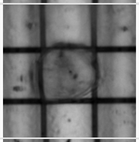	☑
10033	GROUP 2	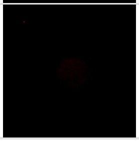	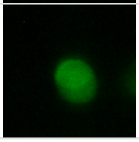	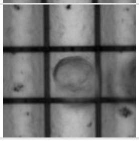	☑

id	Group	PE_0	FITC_1	BRIGHTFIELD_2	
8686	GROUP 2	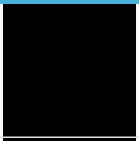	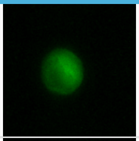	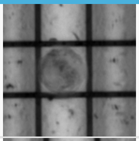	☑
7106	GROUP 2	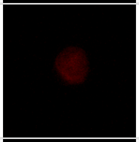	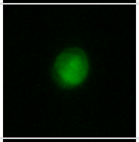	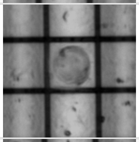	☑
5268	GROUP 2	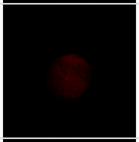	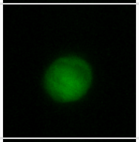	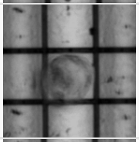	☑
1867	GROUP 2	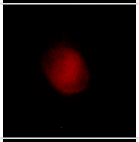	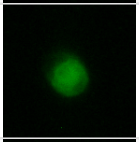	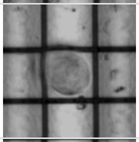	☑
1834	GROUP 2	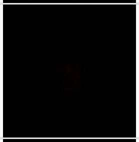	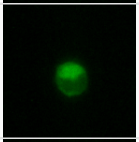	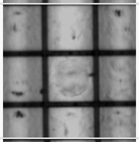	☑
5868	GROUP 2	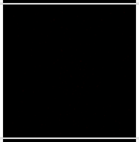	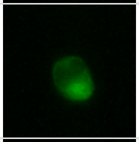	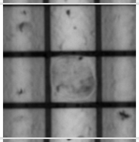	☑
4613	GROUP 2	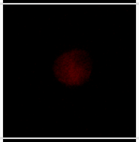	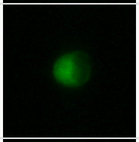	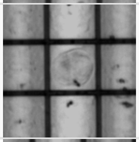	☑
3390	GROUP 2	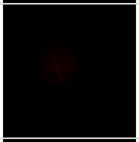	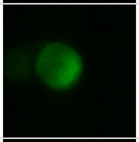	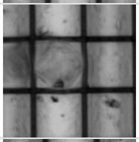	☑
5131	GROUP 2	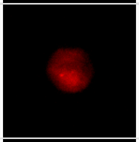	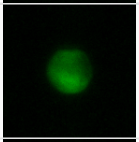	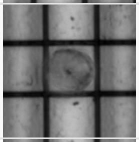	☑
4073	GROUP 2	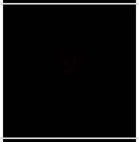	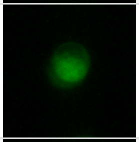	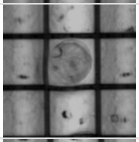	☑
6465	GROUP 2	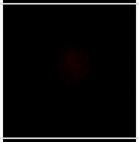	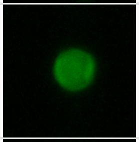	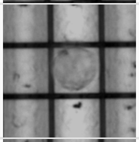	☑
9984	GROUP 2	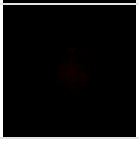	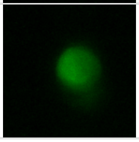	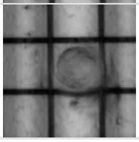	☑

id	Group	PE_0	FITC_1	BRIGHTFIELD_2	
3367	GROUP 2	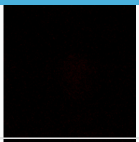	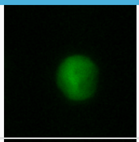	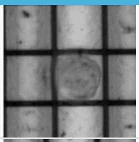	☑
637	GROUP 2	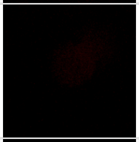	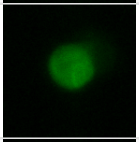	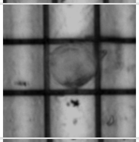	☑
1782	GROUP 2	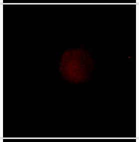	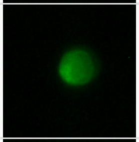	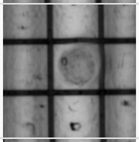	☑
8998	GROUP 2	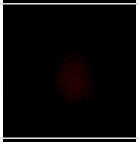	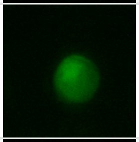	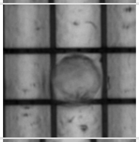	☑
1444	GROUP 2	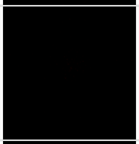	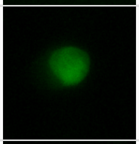	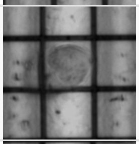	☑
8183	GROUP 2	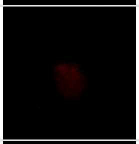	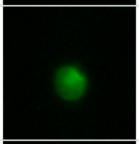	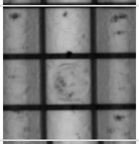	☑
6063	GROUP 2	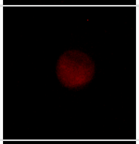	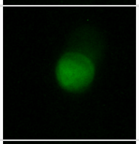	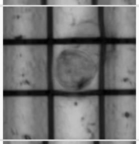	☑
5095	GROUP 2	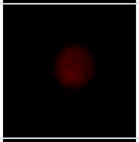	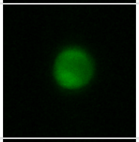	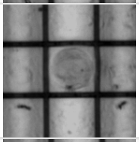	☑
4207	GROUP 2	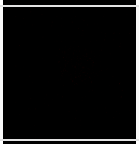	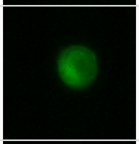	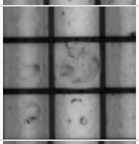	☑
3780	GROUP 2	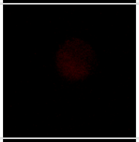	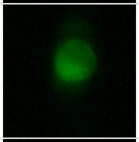	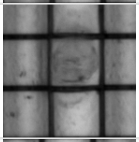	☑
5134	GROUP 2	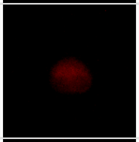	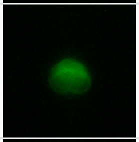	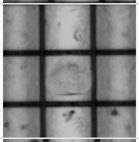	☑
1006	GROUP 2	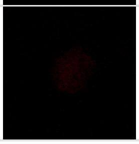	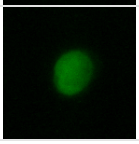	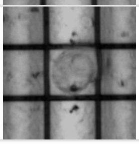	☑

id	Group	PE_0	FITC_1	BRIGHTFIELD_2	
4907	GROUP 2	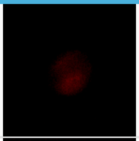	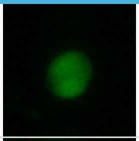	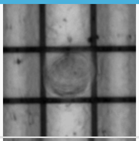	☑
3200	GROUP 2	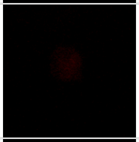	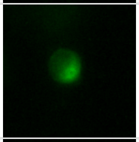	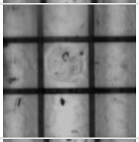	☑
5226	GROUP 2	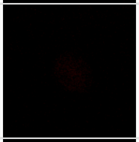	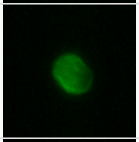	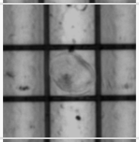	☑
6039	GROUP 2	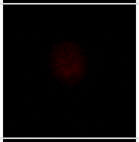	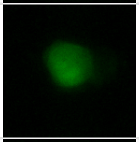	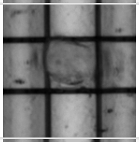	☑
5378	GROUP 2	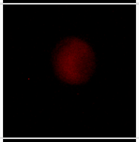	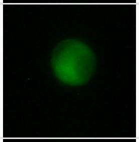	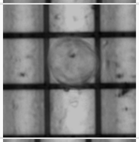	☑
2612	GROUP 2	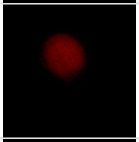	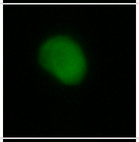	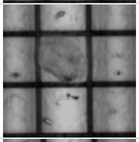	☑
3067	GROUP 2	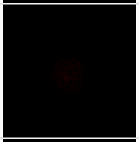	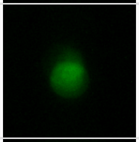	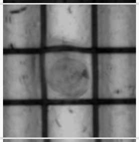	☑
5968	GROUP 2	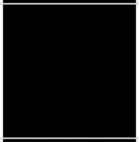	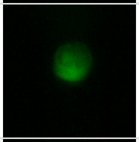	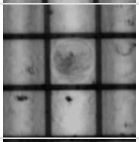	☑
5983	GROUP 2	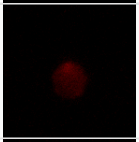	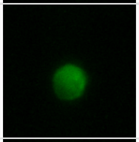	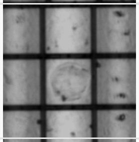	☑
1061	GROUP 2	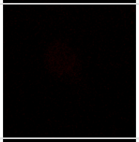	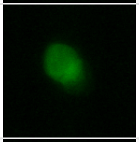	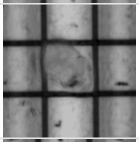	☑
6850	GROUP 2	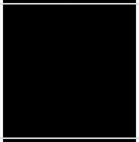	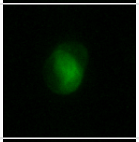	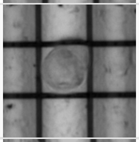	☑
7209	GROUP 2	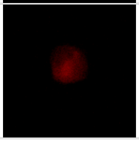	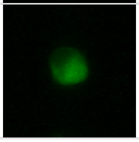	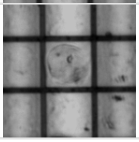	☑

id	Group	PE_0	FITC_1	BRIGHTFIELD_2	
8977	GROUP 2	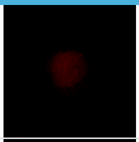	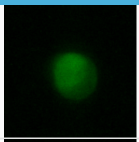	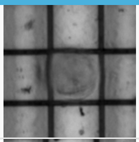	☑
7428	GROUP 2	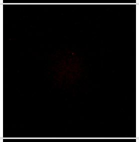	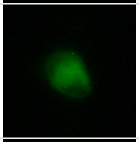	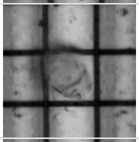	☑
5470	GROUP 2	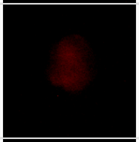	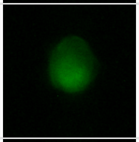	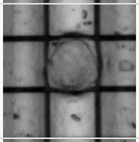	☑
5372	GROUP 2	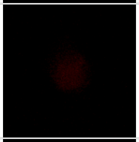	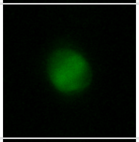	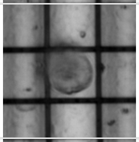	☑
3537	GROUP 2	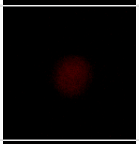	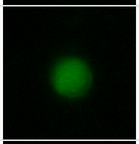	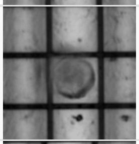	☑
8253	GROUP 2	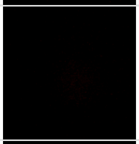	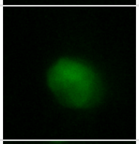	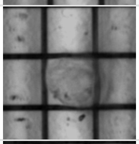	☑
7724	GROUP 2	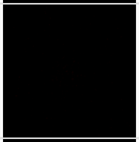	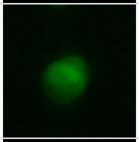	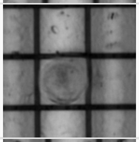	☑
1917	GROUP 2	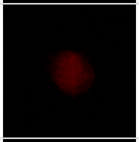	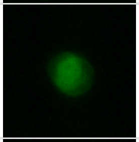	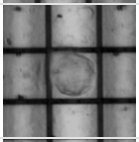	☑
6591	GROUP 2	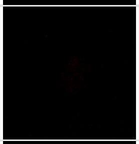	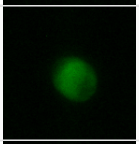	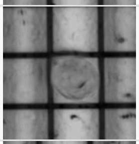	☑
3811	GROUP 2	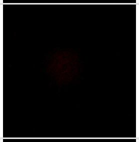	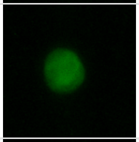	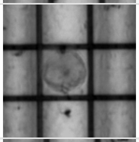	☑
7574	GROUP 2	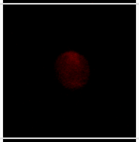	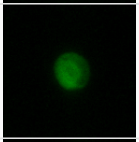	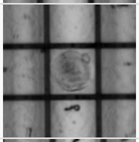	☑
8519	GROUP 2	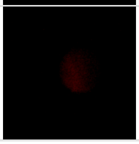	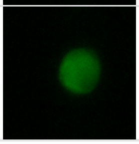	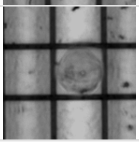	☑

id	Group	PE_0	FITC_1	BRIGHTFIELD_2	
7739	GROUP 2	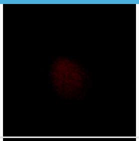	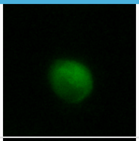	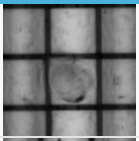	☑
3765	GROUP 2	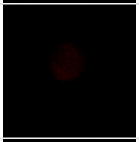	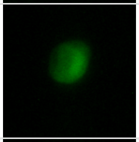	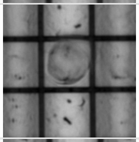	☑
3456	GROUP 2	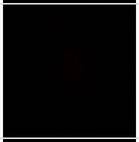	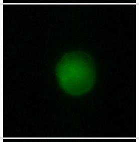	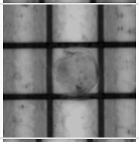	☑
1013	GROUP 2	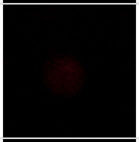	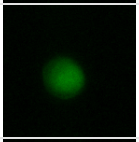	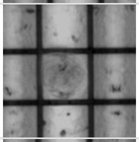	☑
1418	GROUP 2	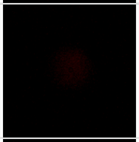	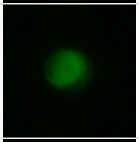	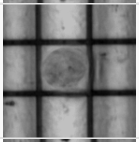	☑
2302	GROUP 2	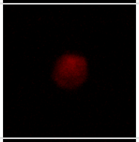	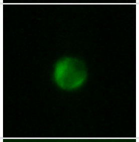	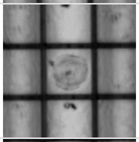	☑
10523	GROUP 2	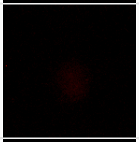	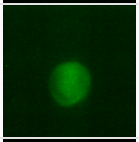	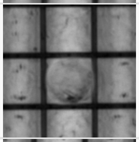	☑
4066	GROUP 2	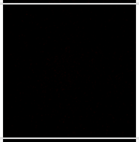	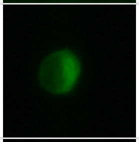	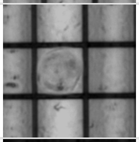	☑
1823	GROUP 2	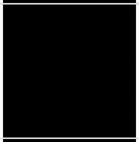	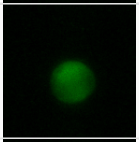	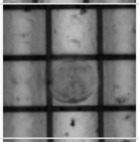	☑
6554	GROUP 2	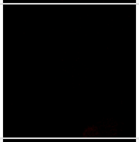	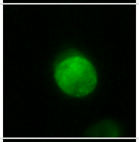	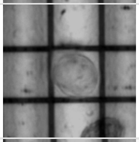	☑
6608	GROUP 2	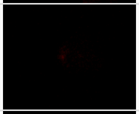	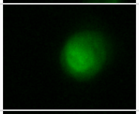	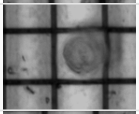	☑
2625	GROUP 2	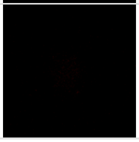	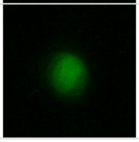	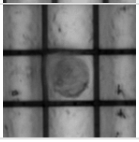	☑

id	Group	PE_0	FITC_1	BRIGHTFIELD_2	
1014	GROUP 2	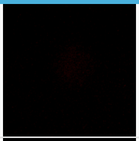	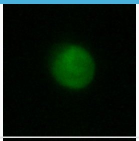	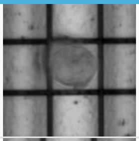	☑
6962	GROUP 2	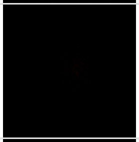	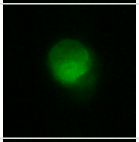	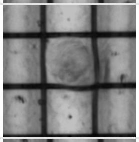	☑
9221	GROUP 2	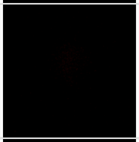	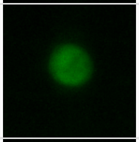	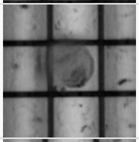	☑
2201	GROUP 2	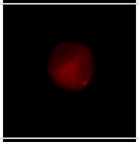	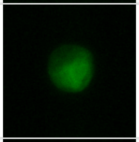	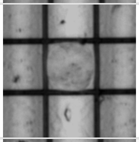	☑
4663	GROUP 2	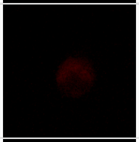	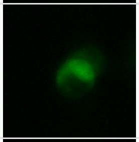	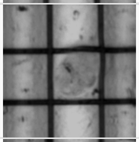	☑
6180	GROUP 2	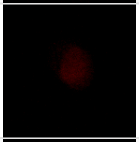	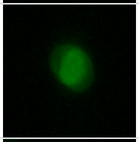	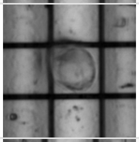	☑
4291	GROUP 2	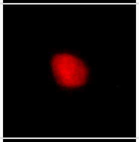	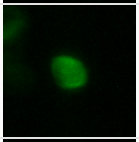	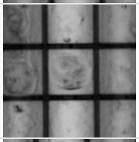	☑
4214	GROUP 2	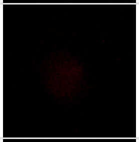	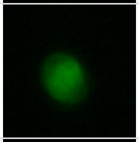	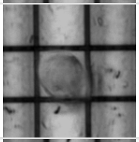	☑
2949	GROUP 2	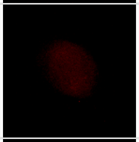	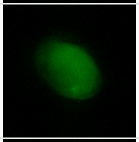	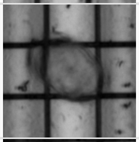	☑
7063	GROUP 2	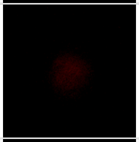	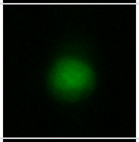	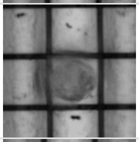	☑
8030	GROUP 2	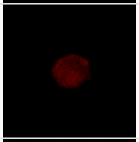	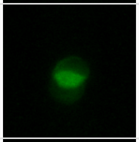	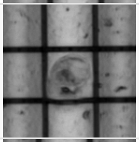	☑
4212	GROUP 2	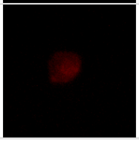	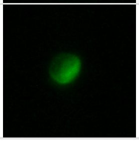	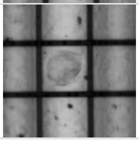	☑

id	Group	PE_0	FITC_1	BRIGHTFIELD_2	
3591	GROUP 2	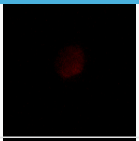	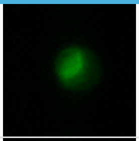	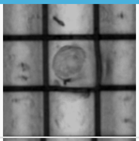	☑
7058	GROUP 2	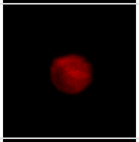	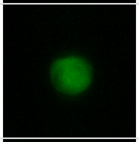	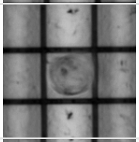	☑
5722	GROUP 2	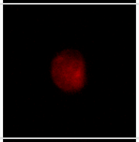	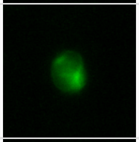	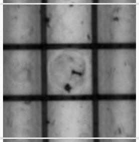	☑
7873	GROUP 2	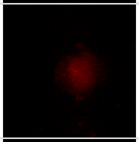	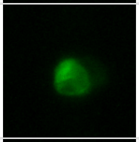	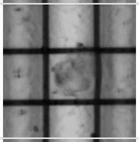	☑
4179	GROUP 2	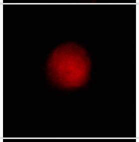	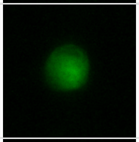	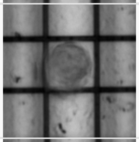	☑
6765	GROUP 2	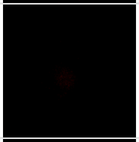	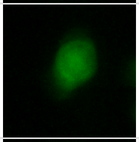	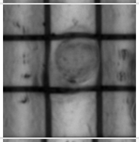	☑
6995	GROUP 2	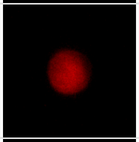	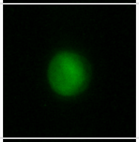	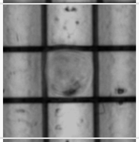	☑
7113	GROUP 2	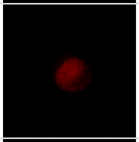	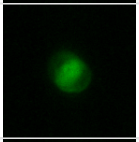	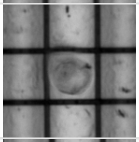	☑
4278	GROUP 2	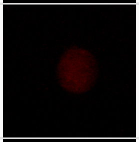	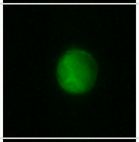	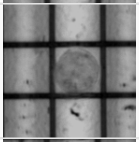	☑
3935	GROUP 2	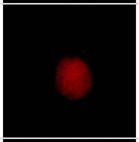	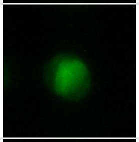	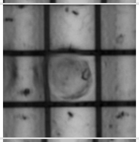	☑
2797	GROUP 2	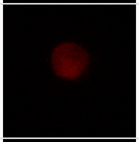	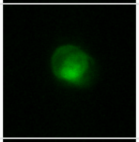	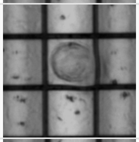	☑
7121	GROUP 2	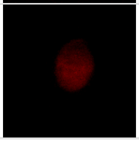	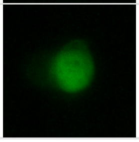	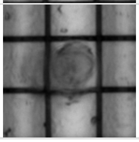	☑

Group 2

id	signal intensity fitc	signal intensity pe
4859	145.31	52.48
3004	144.10	18.43
4714	142.28	10.52
2283	141.85	27.07
2131	269.98	19.39
4874	259.08	99.54
4599	233.96	2.11
2122	232.63	69.63
7312	203.92	66.13
2657	196.01	14.54
6735	192.30	5.92
7522	189.87	30.78
7030	190.24	3.32
6038	188.74	20.27
5964	104.96	33.35
8012	177.94	77.88
3640	173.77	39.24
6397	165.39	4.66
6100	164.14	21.75
2433	161.61	12.45
3002	161.92	53.17
8687	159.09	59.55
9041	159.22	42.86
980	158.42	85.13
5554	104.92	34.39
7306	157.83	38.64
7398	155.62	46.21
3555	152.00	20.02
4315	151.61	57.63
5852	151.47	63.31
7789	149.60	2.04
5862	147.04	5.35
9031	146.27	81.21
6002	142.29	39.45
5494	135.73	24.84
3321	104.58	39.43
1394	133.66	6.06
655	133.04	6.36
8752	131.40	24.51
10104	130.78	16.78
1067	128.46	39.62
6369	128.07	29.07
2045	127.70	18.13
3880	127.05	12.77
7583	102.47	97.73
5622	125.68	4.98
10033	122.37	18.76
8686	121.62	4.48
7106	119.67	34.80
5268	117.99	36.24
1867	117.66	72.28
1834	116.72	11.87
5868	116.58	1.82
4613	104.35	36.85
3390	116.20	18.80
5131	115.87	79.61
4073	115.58	9.28

id	signal intensity fitc	signal intensity pe
6465	115.40	17.69
9984	115.10	14.98
3367	114.25	5.19
637	113.10	11.99
1782	112.45	33.25
8998	110.69	24.88
1444	110.14	6.10
8183	109.84	27.56
6063	109.62	50.49
5095	109.29	41.53
4207	108.75	5.48
3780	107.02	25.70
5134	106.99	45.56
1006	106.72	17.88
4907	106.62	41.60
3200	106.20	19.12
5226	106.12	10.42
6039	106.04	28.03
5378	105.93	48.72
2612	105.78	64.21
3067	105.50	12.38
5968	105.41	2.50
5983	104.89	34.26
1061	104.65	6.84
6850	103.84	4.81
7209	103.65	51.79
8977	103.54	38.38
7428	103.50	9.79
5470	103.43	42.14
5372	103.31	25.00
3537	103.13	35.74
8253	102.92	8.48
7724	102.90	4.60
1917	102.41	42.33
6591	102.30	6.70
3811	101.74	11.94
7574	101.48	38.37
8519	100.92	31.73
7739	100.45	29.09
3765	100.25	27.85
3456	100.08	5.27
1013	100.01	20.81
1418	99.92	18.80
2302	99.55	46.93
10523	99.42	16.62
4066	96.78	3.77
1823	96.23	3.91
6554	159.24	3.97
6608	133.59	7.63
2625	109.50	7.72
1014	96.77	7.75
6962	133.40	8.76
9221	98.81	9.00
2201	105.09	57.20
4663	107.13	28.17
6180	108.05	34.19
4291	109.85	107.47
4214	110.20	15.61
2949	110.31	38.25
7063	110.41	29.99

id	signal intensity fitc	signal intensity pe
8030	110.63	47.05
4212	110.87	35.42
3591	111.86	28.23
7058	112.47	81.39
5722	114.71	59.51
7873	115.15	46.44
4179	117.28	85.40
6765	118.32	20.44
6995	118.91	88.38
7113	122.35	55.62
4278	125.18	32.93
3935	125.90	74.52
2797	126.82	40.72
7121	127.23	55.73
