# Supplementary material for: 3D genome organization in the epithelial-mesenchymal transition spectrum
Source: Genome Biol. 2022 May 30;23:121. doi: 10.1186/s13059-022-02687-x (PMC9150291; doi:10.1186/s13059-022-02687-x)
Supplement: Supplementary file 10 — Additional file 10. Review history. [file 13059_2022_2687_MOESM10_ESM.docx]

Review History

**First round of review**

**Reviewer 1**

**Were you able to assess all statistics in the manuscript, including the appropriateness of statistical tests used?**

No, I do not feel adequately qualified to assess the statistics.

**Comments to author:**

This study explores 3D chromatin conformation in different EMT state cancer cells. The authors refined EMT signature genes and identified association between their gene expression and patient survival. Focusing on the selected EMT genes, the authors identified different chromatin organization patterns between epithelial genes vs mesenchymal genes. The establishment of the new single cell Hi-C technique based on EMT markers is intriguing. However, the study is mostly descriptive, and it is not clear if the results are reflecting the dynamic nature of EMT. The authors have a great model to study temporal changes in the EMT status using the DOX-inducible GRHL2 knockdown system. The Hi-C data in the TGFbeta-treated NMuMG cells has been published by Peiró's group and could be used for the further comparison. It is also not clear how the selection of specific EMT related genes impact on the 3D chromatin analysis, and if the other EMT genes behave similarly. The other major and minor concerns are listed below.

(1) Open chromatin states in mTADs

P.12, the authors stated that "Mesenchymal genes in mTADs maintained in open chromatin states in both epithelial and mesenchymal cells". I am not sure if this conclusion is supported by the data. The compartment analysis seems to suggest that E genes remain active chromatin compartment, while a subset of M genes are converted. The ATA-seq data shows higher signal levels in E genes as compared to M genes. H3K27ac metaplot shows decreased level in PEO1.

(2) Figure 1d

It seems that blue indicates Q4 and red indicates Q1. Please clarify. Figure legend also doesn't match to cancer subtypes shown in the figure.

(3) Single cell analysis

It is not clearly described how the hybrid cells are found. Are they in PEO1 or HEYA8 cell population? Also, the single cell analysis does not seem to be relevant or consistent with the rest of the story.

**Reviewer 2**

**Were you able to assess all statistics in the manuscript, including the appropriateness of statistical tests used?**

Yes, and I have assessed the statistics in my report.

**Comments to author:**

In this manuscript, the authors used Hi-C and ChIP-seq data to dissect the changes of 3D genome organization and epigenome during EMT transition. The authors conclude that A/B compartment changes only happen to a fraction of the mesenchymal genes during this process. Epithelial gene (e gene) residing TADs (e TADs) show decrease in active epigenetic marks and increase in H3K27me3 throughout EMT. Mesenchymal gene (m gene) residing TADs (m TADs) show changes in Hi-C interaction frequency. (So do e TADs.) Using combined immunofluorescence and single cell Hi-C the authors show certain chromosome's conformation can help identify cell clusters of different E-M states. The manuscript includes rich information that could be useful to the genome biology community. But unfortunately the manuscript has many errors, omissions and apparent contradictions that prevent the reviewer from evaluating the validity of the conclusions. At the most basic level, many figure numbers are wrong - it seems the authors updated figures without updating the manuscript. This alone makes the review process very challenging. Also some figures seem to support opposite conclusions. Some figures did not show things they are said to show. I urge the authors to identify these errors and other errors not noted in this review by carefully proofreading the manuscript. Below please find my detailed comments:

Major points:

1. The authors used multiple cell lines to represent the E-M states. However it is unclear if 3D genome differences/similarities among cell lines can be attributed to the E vs M states, as there are other intrinsic differences between the cell lines due to e.g. human individual differences and/or clonal diversity of cancer. The authors have used induced EMT of the same cell line in other studies, which could address this issue.

2. The authors performed GO analysis for the selected E and M genes in each of AA, BB, AB and BA categories and showed that only certain category has GO term enrichment. Has the enrichment analysis taken into account that it is a selected small pool of genes to begin with? For example, the selected pool of E and M genes may already be enriched with the terms regardless of the categories. And the major category picks up the terms because most of the selected genes are in that category.

3. In Fig. 2b, for M genes N=62, yet 4+3+3+11 = 61 in the x label. In Fig. 2c, the AB category should have 4 genes. Based on 2b, one of the AB M genes should have a > 4 fold reduction in HEYA8, could the author point out which one is that in 2c right panels, please? None of the 4 rows in the AB category seem to have that huge reduction in HEYA8.

4. eTADs are shown to have higher active marks such as H3K4me1 and H3K27ac, and lower H3K27me3 in PEO1 than in HEYA8. The compartment identities of the e genes mostly did not change in PEO1 vs HEYA8 when the authors only consider two compartments (A and B). The authors thus conclude that the compartment changes are less important than the epigenetic changes for the e gene. A previous study showed that H3K27me3 enriched region could represent a third compartment with low but positive compartment PC1 value. Thus H3K27me3 regions are traditionally categorized as compartment A but they are in fact different from the active compartment A regions with highly positive PC1 values. Can the authors show not just the sign changes of the PC1, but the actual value changes for the e genes? It could be that the e genes in HEYA8 actually belong to the third intermediate compartment enriched with H3K27me3.

5. Compartment analysis is carried out at the gene level. Why are the epigenetic analyses only at the TAD level? Are the changes of the H3K4me1, H3K27ac, and H3K27me3 for the e and m genes consistent with those for the e and m TADs across the cell lines?

6. A significant portion of the mTADs should have compartment changes since a significant portion of m genes show compartment changes. Yet the active marks did not systematically change in the mTADs. H3K9me3 should be profiled since the B compartment is likely driven by an H3K9me3-HP1 mechanism. This could reconcile the apparent contradiction between the observation here and the prevalent model that the epigenome drive the A/B compartmentalization.

7. Page 12 Line 32: "Interestingly, the M genes and their TADs retained the open chromatin state in the epithelial-like cancer cell lines, suggesting another underlying mechanism could contribute to the regulation of the M genes." For the 11 m genes in the BA category in fig.2c or the 33 dynamic m genes in fig. 2d, are they open chromatin yet in compartment B in the epithelial-like cancer cell lines? Is it legit to call those regions open chromatin from a baseline H3K27ac profile alone? Could the authors show a negative control measurement of H3K27ac in a closed chromatin region?

8. Page 13 Line 26: "Knowing that the mTADs did not show significant enrichment of repressive histone marks,…." The authors only studied H3K27me3 as the repressive mark. What about H3K9me3 and 5mC?

9. In Fig. S3a, for the mTADs that switched from B to A, do they show an increase in ATAC-seq signal?

10. Claims on Page 13-14 about changes in Fig. 4b and c lack statistical tests.

11. Page 13 Line 50: "The interaction scores of both the E and M genes coming into spatial proximity of the other genes within the same TADs increased along the EMT spectrum" according to the Hi-C data. Yet in 3C-qPCR, CDH1, an E gene shows the highest interaction with other genes in the most epithelial-like PEO1 cells. Is this observed in the Hi-C data too? How to reconcile these seemingly contradictory results?

12. Page 15 line 29-34, "Likewise, this pattern of increased interaction frequency in the M state, was also observed between the TES of CDH2 and an upstream enhancer in the CDH2 TAD". It seems that "upstream enhancer" lacks H3K4me1 in all 4 cell lines. How is the "enhancer" defined?

13. Page 15, line 44-46: "In summary, the eTADs adopted an open chromatin state with increased interaction between the E genes with non-EMT genes in the same domain". Is this talking about the E state or M state? E state showed the open chromatin and M state showed the increased interaction according to Fig. 4b.

14. Page 15 line 49-54: "Whereas in the mTADs, the regulation of the M genes was more dependent on the changes in the local chromatin conformation". Fig. 4c actually shows that H3K27me3 decreased at the TSS of M genes in the M state. How is the "more dependent" conclusion achieved? Can the authors do a correlation analysis between the EMT score and M gene TSS H3K27me3? Note this is different from Fig. 3f since that figure uses whole mTAD H3K27me3 levels. The H3K27me3 could be quite local. Also most data in this paper only show associations but not causality (no perturbation was performed except in one case), so the authors cannot conclude about dependence as stated here.

15. In Fig. 5a what are the green stripes? Where are the proximal and distal enhancers mentioned in the text and in Fig. 5b? The green stripes seem to be two enhancer regions but they do not correspond to the E-P loops in the HEYA8 shown by the blue curves. The authors state: "From the Hi-C data, we observed 2 enhancer to promoter (EP) loops which were present between SNAI2 and an upstream distal enhancer and the proximal enhancer in HEYA8 (Figure 5a)". And based on the blue curves they are different from the green stripes. Because of this error it is hard to assess if the 3C-qPCR is consistent with the Hi-C data here.

16. Page 16, line 41-48: "Knowing that SNAI1 binds to the SNAI2 E boxes and recruits HDAC to repress transcription, SNAI2 repression in PEO1 could result from the endogenous SNAI1 expression and the concomitant H3K27me3 enrichment (Figure 5b, 5d)." How is SNAI1 linked to H3K27me3 enrichment? It was mentioned before SNAI1 recruit HDAC. Is H3K27me3 really enriched at this enhancer region in PEO1? Or is it due to H3K27ac removal?

17. What is the genomic resolution of the single-cell Hi-C analyses? The authors could expand significantly on the single-cell Hi-C analyses if the resolution is high enough. For example instead of using the rmsd for the cluster, the authors could use the cell-cell compaction of E-TADs and/or m-TADs, single-cell A/B compartment PC1 of these TADs, or the E/M-genes' P-E loops of the single cell Hi-C data to perform clustering and see which feature(s) generate clusters that correspond to the ground truth cell states identified by immunofluorescence.

Minor points:

18. Fig. 1C: The circular shape of this plot is unnecessary and only increases the difficulty of reading it.

19. Fig. S1 is introduced after Fig. S2.

20. Page 7 line 29: "Figure S2a" should be S2b, also this is introduced after S2d. The numbering of the figures should following the order they are introduced in.

21. Page 7 line 41: "Figure 2a" should be 2b.

22. Page 8 line 10 and 12: "Figure S1b" should be S2C

23. Supple Table 2e: Title says "mesenchymal genes in 'BA" yet table says "AA (Epithelial genes, n= 64)".

24. Page 9 line 27-29: Figure numbers are wrong.

25. "Evidently, majority of the E genes remained stable in the AA/BB compartments (n = 64, 72.7%, Supplementary table 3a) across the 6 cancer cell lines (Figure 2d)." I only see five cell lines in Figure 2d. Similarly many other places mentioned 6 cell lines yet only 5 are shown in figures.

26. Page 13 line 55: "Figure 4b" should be Figure 4bc since both the E and M genes are discussed in this sentence.

27. Terms like TSS and TES were not defined.

28. Overall too many speculations are mixed with observations in the results section. I suggest moving most of the speculations to the discussions section.

29. Page 18 line 46-49: "The TAD boundaries were shown to be depleted in DNA-DNA contacts in both the ensemble and single cell Hi-C matrices (Figure 6c)." Where are the single cell matrices in Fig. 6c? The left label says "based on bulk HiC".

30. In figure 6f and S4d, which cell is in which state? The authors argue that chr10 allow clustering of cells into 3 groups that corresponds to the E, hybrid and M clusters. This is not shown in the two figures.

**Authors Response**

**Point-by-point responses to the reviewers’ comments:**

Reviewer #1:

This study explores 3D chromatin conformation in different EMT state cancer cells. The authors refined EMT signature genes and identified association between their gene expression and patient survival. Focusing on the selected EMT genes, the authors identified different chromatin organization patterns between epithelial genes vs mesenchymal genes.

*Response: We thank the reviewer for the summary, and we sought to provide the EMT field with more insights on role of 3D genome in the regulation of EMT genes.*

The establishment of the new single cell Hi-C technique based on EMT markers is intriguing.

*Response: We agree with the reviewer, and we see the potential in this technique to answer the heterogenous EMT states that follows the dynamic phases of the EMT spectrum. However, low throughput is the limitation of this technique. We did include this in the Discussion section.*

However, the study is mostly descriptive, and it is not clear if the results are reflecting the dynamic nature of EMT. The authors have a great model to study temporal changes in the EMT status using the DOX-inducible GRHL2 knockdown system.

*Response: We thank the reviewer for the comment. Using the Tet-inducible GRHL2 knockdown system, we have shown that there exists a heterogenous response to GRHL2 induction in our previous publication (Figure 3c, Chung, V.Y., Tan, T.Z., Ye, J. et al. 2019. Commun Biol 2, 272 (2019). https://doi.org/10.1038/s42003-019-0506-3).*

*From the staining of GRHL2 and EpCAM in these single cell populations from the inducible system, it was apparent that the expression level of GRHL2 and EpCAM followed a highly correlative gradient (Figure S8b in revised MS). As bulk Hi-C provides 3D genome data at the population level of cells, we will not be able to deconvolute the heterogenous EMT states observed during the induction of GRHL2 in the Tet-inducible GRHL2 knockdown system if we perform Hi-C on the bulk samples. Therefore, we assessed 3D genome structure of the Tet-inducible GRHL2 knockdown system on a single cell depth, which we have included in Figure 6 of the revised manuscript.*

The Hi-C data in the TGFbeta-treated NMuMG cells has been published by Peiró’s group and could be used for the further comparison.

*Response: We thank the reviewer for the comment. We extracted the EMT TADs from the Hi-C data in the TGFbeta-treated NmuMG cells published by Peiró’s group, based on the mouse orthologs of the EMT genes in human. We observed that the ATAC-seq signal was higher in both eTADS and mTADs in 24hr-TGFbeta-treated NmuMG cells (Rebuttal Figure 1 shown below), suggesting that a wider extent of the chromatin had undergone compaction. However, the median size of the TADs obtained from the NmuMG Hi-C data were ~1.5Mb. In our analysis, we utilized Hi-C datasets that had sufficient sequencing coverage to achieve a TAD size of ~600Kb. Therefore, the bigger EMT TADs defined in the exiting NmuMG Hi-C data could result from the fact that the sequencing depth was not deep enough. We thus could not incorporate this dataset into our analysis. There might contain smaller sub-domains that could regulate the EMT genes from the NmuMG Hi-C data, which could be further uncovered upon deeper sequencing.*

*Rebuttal Figure 1: ATAC-seq signal in TGFbeta-treated NmuMG cells (TGFbeta treatment at 0hr, 8hr and 24hr).*

It is also not clear how the selection of specific EMT related genes impact on the 3D chromatin analysis, and if the other EMT genes behave similarly.

*Response: We thank the reviewer for the comment. However, the hallmark EMT signature from Molecular Signatures Database (MSigDB) represent EMT genes that are derived from various founder gene sets. Similarly, our EMT gene signature (Tan et. al., 2014, EMBO Mol Med (2014)) 6: 1279–1293 was obtained from a collective of pan-cancer expression datasets. Given that EMT is a context-specific process, there will be EMT genes that are only implicated in the transition in different context. Therefore, by refining the two gene signatures, we aim to derive a set of epithelial and mesenchymal genes that will enable us to study those EMT genes that are more commonly regulated in during the transition. We have also assessed the histone distribution at another set of 77 epithelial and 13 mesenchymal genes, that were exclusive to the EMT cell-line specific signature. We observed the eTADs (n=77 epithelial genes, Rebuttal Figure 2 below) in the mesenchymal-like cell lines were enriched with H3K27me3 while the mTADs (n=13 mesenchymal genes, Rebuttal Figure 2 below) did not show enrichment of H3K27me3 in the epithelial-like cell lines. This is in agreement with the EMT TADs defined by the 88 epithelial and 61 mesenchymal genes described in our manuscript.*

*Rebuttal figure 2: Heatmap of histone distribution in EMT TADs defined by 77epithelial and 13 mesenchymal genes, which are exclusive to the EMT cell-line signature. The 5’ and 3’ TAD boundaries are denoted by “5’B” and “3’B” respectively. The histone marks are coloured as accordingly, H3K27ac – green, H3K27me3 – black and H3K9me3 – indigo.*

(1) Open chromatin states in mTADs

P.12, the authors stated that "Mesenchymal genes in mTADs maintained in open chromatin states in both epithelial and mesenchymal cells". I am not sure if this conclusion is supported by the data. The compartment analysis seems to suggest that E genes remain active chromatin compartment, while a subset of M genes are converted. The ATAC-seq data shows higher signal levels in E genes as compared to M genes. H3K27ac metaplot shows decreased level in PEO1.

*Response: We thank the reviewer for the comment. We rephrased the section title to “Changes in H3K27me3 in the mTADs and M genes were less associated to the EMT states.*

(2) Figure 1c

It seems that blue indicates Q4 and red indicates Q1. Please clarify. Figure legend also doesn't match to cancer subtypes shown in the figure.

*Response: We thank the reviewer for the clarification. We now use purple and orange colours to represent the survival plots of epithelial and mesenchymal genes in the cancer datasets used. In each plot, the 1st quartile is represented by solid line, while 4th quartile is represented by a dotted line.*

(3) Single cell analysis

It is not clearly described how the hybrid cells are found. Are they in PEO1 or HEYA8 cell population?

*Response: We thank the reviewer for the comment. We would like to clarify that PEO1 and HEYA8 cells were mixed in a population prior to single cell isolation, therefore we are not able to discern if they are PEO1 or HEYA8 cells as the purpose was to mimic a heterogenous population of EMT states.*

Also, the single cell analysis does not seem to be relevant or consistent with the rest of the story.

*Response: We thank the reviewer for the comment, we have expanded the section of scHi-C analysis and included scHi-C data generated from a Tet-inducible EMT model, using OVCA429 shGRLH2 TetR cell line, where we were able to obtain a heterogenous population of EMT states via the induction of GRHL2. The data was incorporated in Figure 6 in the revised manuscript. Due to the limited number of single cells being analysed in the scHi-C profiling, a more in-depth analysis on the compartments and TADs were not feasible for this current study. The aim of this manuscript is to demonstrate the feasibility of performing scHi-C profiling using the dielectrophoresis system. However, we do have ongoing projects in the lab to expand the current analysis.*

Reviewer #2:

In this manuscript, the authors used Hi-C and ChIP-seq data to dissect the changes of 3D genome organization and epigenome during EMT transition. The authors conclude that A/B compartment changes only happen to a fraction of the mesenchymal genes during this process. Epithelial gene (e gene) residing TADs (e TADs) show decrease in active epigenetic marks and increase in H3K27me3 throughout EMT. Mesenchymal gene (m gene) residing TADs (m TADs) show changes in Hi-C interaction frequency. (So do e TADs.) Using combined immunofluorescence and single cell Hi-C the authors show certain chromosome's conformation can help identify cell clusters of different E-M states. The manuscript includes rich information that could be useful to the genome biology community.

*Response: We thank the reviewer for the kind comments to highlight the impact of this study on the Genome Biology community. We greatly appreciate the reviewer’s patience and constructive comments in the review process.*

But unfortunately, the manuscript has many errors, omissions and apparent contradictions that prevent the reviewer from evaluating the validity of the conclusions. At the most basic level, many figure numbers are wrong - it seems the authors updated figures without updating the manuscript. This alone makes the review process very challenging.

*Response: We apologise to the reviewer for those editorial errors, and we thank the reviewer for painstakingly noting out the errors in the editorial process. We have made significant corrections in the revised manuscript accordingly.*

Also some figures seem to support opposite conclusions. Some figures did not show things they are said to show. I urge the authors to identify these errors and other errors not noted in this review by carefully proofreading the manuscript.

*Response: We thank the reviewer for pointing this out. We have revised the manuscript to increase the clarity and resolved confusion for a smoother reading.*

Below please find my detailed comments:

Major points:

1. The authors used multiple cell lines to represent the E-M states. However, it is unclear if 3D genome differences/similarities among cell lines can be attributed to the E vs M states, as there are other intrinsic differences between the cell lines due to e.g. human individual differences and/or clonal diversity of cancer.

*Response: We acknowledge that there might be intrinsic and structural differences between the cell lines used in this study. However, the collective of EMT genes have been shown to be very robust between different cancer types with different cells-of-origin as shown in Figure 3 of our previous publication (Tan et. al., 2014, EMBO Mol Med (2014) 6: 1279–1293), where we were able to stratify the pan-cancer tumours along the EMT spectrum. Indeed, we did observe cell type variability at the SNAI2 locus with cell type specific interaction differences to its distal and proximal enhancers. However, at a broader scale of the collective EMT genes, we still observed the consistent enrichment in H3K27me3 mark in the eTADs of the mesenchymal-like cell lines, while the mTADs were less associated with the changes in the histone modifications between the EMT states. This is in accordance with the previous research showing that TADs are largely invariant across cell lines (Pope, B. D., Ryba, T., Dileep, V., Yue, F., Wu, W., Denas, O., et al. (2014). Nature 515, 402–405. doi: 10.1038/nature13986; Rao, S. S. P., Huntley, M. H., Durand, N. C., Stamenova, E. K., Bochkov, I. D., Robinson, J. T., et al. (2014). Cell 159, 1665–1680. doi: 10.1016/j.cell.2014.11.021; Schmitt, A. D., Hu, M., Jung, I., Xu, Z., Qiu, Y., Tan, C. L., et al. (2016). Cell Rep. 17, 2042–2059. doi: 10.1016/j.celrep.2016.10.061). Nevertheless, we do agree with the reviewer that there would be clonal diversity especially in the setting of intra-tumor heterogeneity. This is the reason why we incorporated the scHi-C analysis (see the next response below) as the final touch of the manuscript to suggest the possible avenue for future research.*

The authors have used induced EMT of the same cell line in other studies, which could address this issue.

*Response: We acknowledge that the transition process in EMT is important. Hence, we sought to understand the transition EMT from a wider scope by using a panel of well-annotated cancer cell line model in terms of EMT states before interrogating our results in an EMT-inducible system developed in our lab. In the revised manuscript, we included scHi-C data generated from a Tet-inducible EMT model, using OVCA429 shGRLH2 TetR cell line, where we can obtain a heterogenous population of EMT states via the induction of GRHL2. The data was incorporated in Figure 6 in the revised manuscript. Due to the limited number of single cells being analysed in the scHi-C profiling, a more in-depth analysis on the compartments and TADs were not feasible for this current study. The aim of this manuscript is to demonstrate the feasibility of performing scHi-C profiling using the dielectrophoresis system. However, we do have ongoing projects in the lab to expand the current analysis.*

2. The authors performed GO analysis for the selected E and M genes in each of AA, BB, AB and BA categories and showed that only certain category has GO term enrichment. Has the enrichment analysis taken into account that it is a selected small pool of genes to begin with? For example, the selected pool of E and M genes may already be enriched with the terms regardless of the categories. And the major category picks up the terms because most of the selected genes are in that category.

*Response: We agree with the reviewer that these are well-characterized, pre-defined EMT genes. However, the EMT genes have specific and diverse functions within the EMT programme. This is another point that we would like to get the messages across that not all EMT genes are equal in terms of the regulation at the 3D genome, epigenome, and transcription controls. Subsets of epithelial or mesenchymal gene did show the same pattern of regulation. However, there did exist diverse regulatory mechanisms within different categories of EMT genes (Chung, V.Y., Tan, T.Z., Ye, J. et al. 2019. Commun Biol 2, 272 (2019). https://doi.org/10.1038/s42003-019-0506-3 and this study). Therefore, the GO analysis would reveal to us that whether there might be specific functional grouping for these subsets of EMT genes. Particularly for the mesenchymal genes, where we see higher dynamics that the enrichment of GO specific functions would be very intriguing.*

3. In Fig. 2b, for M genes N=62, yet 4+3+3+11 = 61 in the x label. In Fig. 2c, the AB category should have 4 genes.

*Response: We thank the reviewer for highlighting the discrepancy, 1 mesenchymal gene was omitted due to filtering of lowly expressed genes. We have updated that in Figure 2b.*

Based on 2b, one of the AB M genes should have a > 4 fold reduction in HEYA8, could the author point out which one is that in 2c right panels, please? None of the 4 rows in the AB category seem to have that huge reduction in HEYA8.

*Response: We thank the reviewer for highlighting the discrepancy. We have corrected the boxplot in the revised manuscript (Figure 2b) to follow the Tukey style, as the previous boxplot was showing the “minimum” and “maximum” values.*

4. eTADs are shown to have higher active marks such as H3K4me1 and H3K27ac, and lower H3K27me3 in PEO1 than in HEYA8. The compartment identities of the e genes mostly did not change in PEO1 vs HEYA8 when the authors only consider two compartments (A and B). The authors thus conclude that the compartment changes are less important than the epigenetic changes for the e gene. A previous study showed that H3K27me3 enriched region could represent a third compartment with low but positive compartment PC1 value. Thus H3K27me3 regions are traditionally categorized as compartment A but they are in fact different from the active compartment A regions with highly positive PC1 values. Can the authors show not just the sign changes of the PC1, but the actual value changes for the e genes? It could be that the e genes in HEYA8 belong to the third intermediate compartment enriched with H3K27me3.

*Response: We thank the reviewer for the comment. We have changed the PC1 component to show the PC1 values instead in the revised Figure 2c and 2d. We also included a sub-compartment analysis to show that the epithelial genes in the A compartment of HEYA8 corresponds to the active sub-compartments A.1.1 and A.1.2. The sub-comparments are called using CALDER (https://github.com/CSOgroup/CALDER) where sub-compartments A.2.2, B.1.1. and B.1.2 are shown to be enriched in H3K27me3, compared to A.1.1 and A.1.2 which were less enriched in H3K27me3 (Liu, Y. et al. Systematic inference and comparison of multi-scale chromatin sub-compartments connects spatial organization to cell phenotypes. Nat Commun 12, 2439 (2021). https://doi.org/10.1038/s41467-021-22666-3). We added a new section in the revised manuscript for the sub-compartment analysis.*

5. Compartment analysis is carried out at the gene level. Why are the epigenetic analyses only at the TAD level?

*Response: We thank the reviewer for the comment. We have included the correlation of histone modifications to the compartments A and B in the revised Figure S3c.*

Are the changes of the H3K4me1, H3K27ac, and H3K27me3 for the e and m genes consistent with those for the e and m TADs across the cell lines?

*Response: We thank the reviewer for the comment. We have included a figure showing the correlation between the EMT scores and histone marks at the E and M genes (Figure S4d). The results of the data are consistent with Figure 3f.*

6. A significant portion of the mTADs should have compartment changes since a significant portion of m genes show compartment changes. Yet the active marks did not systematically change in the mTADs. H3K9me3 should be profiled since the B compartment is likely driven by an H3K9me3-HP1 mechanism. This could reconcile the apparent contradiction between the observation here and the prevalent model that the epigenome drive the A/B compartmentalization.

*Response: We thank the reviewer for the comment. We have shown that the H3K9me3 is correlated to compartment B in Figure S3c of the revised manuscript, and the H3K9me3 levels in the mTADs were shown to remain relatively unchanged between the cancer cell lines of the EMT spectrum in Figure 3e of the revised manuscript.*

7. Page 12 Line 32: "Interestingly, the M genes and their TADs retained the open chromatin state in the epithelial-like cancer cell lines, suggesting another underlying mechanism could contribute to the regulation of the M genes." For the 11 m genes in the BA category in fig.2c or the 33 dynamic m genes in fig. 2d, are they open chromatin yet in compartment B in the epithelial-like cancer cell lines? Is it legit to call those regions open chromatin from a baseline H3K27ac profile alone? Could the authors show a negative control measurement of H3K27ac in a closed chromatin region?

*Response: We thank the reviewer for the comment. We have rephrased it to “Interestingly, the M genes and their TADs show less associated with the changes in histone modification between the EMT states, suggesting that another underlying mechanism could contribute to the regulation of the M genes.”*

*In addition, we have shown that the compartments A and B correlate with the active histone marks (H3K4me1, H3K4me3 and H3K27ac) and repressive marks (H3K27me3 and H3K9me3) respectively in figure S3c of the revised manuscript.*

8. Page 13 Line 26: "Knowing that the mTADs did not show significant enrichment of repressive histone marks,…." The authors only studied H3K27me3 as the repressive mark. What about H3K9me3 and 5mC?

*Response: We thank the reviewer for the comment. We have included H3K9me3 in the analysis of the EMT genes and their TADs and observed that H3K9me3 levels in the EMT TADs remains relatively unchanged between the epithelial- and mesenchymal-like cell lines (Figure 3d and 3e in revised manuscript). We have also looked at the 5mC of EMT genes in the EMT TADs of PEO1 and HEYA8 and shown that the methylation levels of the EMT genes associates with the EMT state (Figure S5a), which was also reported in our previous publication (Chung, V.Y., Tan, T.Z., Ye, J. et al. 2019. Commun Biol 2, 272 (2019). https://doi.org/10.1038/s42003-019-0506-3.). The methylation levels of the genes in mTADs were higher than the eTADs in the E state (pPEO1-eTAD vs PEO1-mTAD < 0.05, Figure S5b). However, the methylation levels of the genes in the EMT TADs were generally higher in the M state (pPEO1-eTAD vs HEYA8-eTAD < 0.0001, pPEO1-mTAD vs HEYA8-mTAD < 0.0001. Figure S5b).*

9. In Fig. S3a, for the mTADs that switched from B to A, do they show an increase in ATAC-seq signal?

*Response: We thank the reviewer for the comment. As shown in the Rebuttal Figure 3, we observed an increase in ATAC-seq signal in mTADs in HEYA8 that switched from B (PEO1) to A (HEYA8).*

*Rebuttal Figure 3: ATAC-seq signal of mTADs that switched from compartment B in PEO1 to compartment A in HEYA8.*

10. Claims on Page 13-14 about changes in Fig. 4b and c lack statistical tests.

*Response: We thank the reviewer for the comment, and we have extracted the interaction frequency from the aggregate plots in Figure 4b and 4c and included the statistical analysis in the revised manuscript.*

11. Page 13 Line 50: "The interaction scores of both the E and M genes coming into spatial proximity of the other genes within the same TADs increased along the EMT spectrum" according to the Hi-C data. Yet in 3C-qPCR, CDH1, an E gene shows the highest interaction with other genes in the most epithelial-like PEO1 cells. Is this observed in the Hi-C data too? How to reconcile these seemingly contradictory results?

*Response: We thank the reviewer for highlighting this and we have revised this in our manuscript. As the bulk Hi-C data represents an aggregate of the chromatin interactions occurring at the epithelial and mesenchymal genes, we acknowledged in the discussion of the revised manuscript that further 3C-qPCR validation at more loci of epithelial genes could explain the differences observed in 3C versus Hi-C data. However, the agreement between the 3C and Hi-C data at the M genes suggested that the regulation of the M genes could be contributed by the increased chromatin interaction frequency occurring between the M genes and their regulatory elements.*

*“Within the EMT TADs, rather than binary changes in the local chromatin structure, such as the formation or loss of enhancer-promoter interactions, it is the quantitative changes such as contact frequency between the enhancer and its promoter that influences the transcriptional activity of the EMT gene. This was observed in both CDH2 and SNAI2 mTADs, the increase in interaction frequency between the genes and its regulatory elements indeed corresponded to an increase in gene expression, despite the enhancer-promoter interactions still being present even in the E state, albeit at a lower frequency. Although the increase in interaction frequency between the E genes and their regulatory elements was not observed in bulk Hi-C data but via 3C-qPCR, it could be further validated with more 3C-qPCR at other epithelial genes, as the bulk HiC provides an aggregate of the chromatin interaction frequency at the 88 epithelial genes.”*

12. Page 15 line 29-34, "Likewise, this pattern of increased interaction frequency in the M state, was also observed between the TES of CDH2 and an upstream enhancer in the CDH2 TAD". It seems that "upstream enhancer" lacks H3K4me1 in all 4 cell lines. How is the "enhancer" defined?

*Response: We apologise to the reviewer and thank the reviewer for the comment. We have corrected the scale of the histone signal tracks. The ChromHMM data has shown there are 2 enhancer regions, according to HEYA8 ChromHMM analysis, at the partnering locus of the CDH2 TES (Rebuttal Figure 4).*

*Rebuttal Figure 4: ChromHMM of the partnering locus of CDH2 TES.*

13. Page 15, line 44-46: "In summary, the eTADs adopted an open chromatin state with increased interaction between the E genes with non-EMT genes in the same domain". Is this talking about the E state or M state? E state showed the open chromatin and M state showed the increased interaction according to Fig. 4b.

*Response: We thank the reviewer for highlighting this and apologise for the confusion. We have revised the sentence in the manuscript.*

*“In summary, the eTADs adopted an open chromatin state in the E state compared to the M state with 3C-qPCR showing increased interaction between selected E genes such as CDH1 and CDH3 with non-EMT genes in the same domain. In the mTADs, both 3C-qPCR and Hi-C suggested that the changes in the local chromatin conformation with the M genes such as CDH2 could play a bigger role in the transcriptional activity of these gene (Figure 4f).”*

14. Page 15 line 49-54: "Whereas in the mTADs, the regulation of the M genes was more dependent on the changes in the local chromatin conformation". Fig. 4c actually shows that H3K27me3 decreased at the TSS of M genes in the M state. How is the "more dependent" conclusion achieved? Can the authors do a correlation analysis between the EMT score and M gene TSS H3K27me3?

*Response: We thank the reviewer for the comment. In our previous publication, we showed that the H3K27me3 and H3K9me3 levels at the TSS of the M genes were less consistent with the EMT states along the spectrum but still showed higher levels of active histone marks such as H3K4me1 and H3K4me3 (Figure 4a, Chung, V.Y., Tan, T.Z., Ye, J. et al. 2019. Commun Biol 2, 272 (2019). https://doi.org/10.1038/s42003-019-0506-3). It was also observed that the H3K27ac was still enriched at the TSS of the M genes in the E state, as compared to the removal of H3K27ac at the E genes in the M state (Figure 4b).*

Note this is different from Fig. 3f since that figure uses whole mTAD H3K27me3 levels. The H3K27me3 could be quite local. Also most data in this paper only show associations but not causality (no perturbation was performed except in one case), so the authors cannot conclude about dependence as stated here.

*Response: We acknowledge this point, and we have rephased our conclusion in the manuscript.*

*“In the mTADs, both 3C-qPCR and Hi-C suggested that the changes in the local chromatin conformation with the M genes such as CDH2 could play a bigger role in the transcriptional activity of these gene (Figure 4f).”*

15. In Fig. 5a what are the green stripes? Where are the proximal and distal enhancers mentioned in the text and in Fig. 5b? The green stripes seem to be two enhancer regions but they do not correspond to the E-P loops in the HEYA8 shown by the blue curves.

*Response: We apologise to the reviewer and thank the reviewer for highlighting the discrepancy. We have revised the paragraph in the manuscript to clarify this.*

*“From the Hi-C data, we observed 2 loops arising from the SNAI2 promoter, of which one of the loops is an enhancer-promoter (E-P) loop (Figure 5a). In addition, there is a proximal enhancer to SNAI2 that consists of the binding site of GRHL2 (Figure 5a), a gatekeeper of the E state. Although no loops were detected between the proximal enhancer and SNAI2 in the Hi-C data, knowing that GRHL2 regulates E genes via CpG methylation and nucleosome remodelling during the intermediate states of EMT/MET23, we then questioned if the binding of GRHL2 at the SNAI2 TAD would contribute to the repression of SNAI2 in the E state by using 3C-qPCR.”*

The authors state: "From the Hi-C data, we observed 2 enhancer to promoter (EP) loops which were present between SNAI2 and an upstream distal enhancer and the proximal enhancer in HEYA8 (Figure 5a)". And based on the blue curves they are different from the green stripes. Because of this error it is hard to assess if the 3C-qPCR is consistent with the Hi-C data here.

*Response: We apologise to the reviewer and thank the reviewer for highlighting the discrepancy. We have revised the paragraph in the manuscript to clarify this. The green stripes have been annotated to show the locations of the distal and proximal enhancer together with the loops validated in 3C-qPCR.*

*“From the Hi-C data, we observed 2 loops arising from the SNAI2 promoter, of which one of the loops is an enhancer-promoter (E-P) loop (Figure 5a). In addition, there is a proximal enhancer to SNAI2 that consists of the binding site of GRHL2 (Figure 5a), a gatekeeper of the E state. Although no loops were detected between the proximal enhancer and SNAI2 in the Hi-C data, knowing that GRHL2 regulates E genes via CpG methylation and nucleosome remodelling during the intermediate states of EMT/MET23, we then questioned if the binding of GRHL2 at the SNAI2 TAD would contribute to the repression of SNAI2 in the E state by using 3C-qPCR.”*

16. Page 16, line 41-48: "Knowing that SNAI1 binds to the SNAI2 E boxes and recruits HDAC to repress transcription, SNAI2 repression in PEO1 could result from the endogenous SNAI1 expression and the concomitant H3K27me3 enrichment (Figure 5b, 5d)." How is SNAI1 linked to H3K27me3 enrichment? It was mentioned before SNAI1 recruit HDAC. Is H3K27me3 really enriched at this enhancer region in PEO1? Or is it due to H3K27ac removal?

*Response: We apologise to the reviewer and thank the reviewer for the suggestion. We have revised the sentence to “Knowing that SNAI1 binds to the SNAI2 E-boxes and recruits HDAC to repress transcription54, SNAI2 repression in PEO1 could result from the endogenous SNAI1 expression and the concomitant H3K27me3 enrichment at the SNAI2 TSS with the removal of H3K27ac at the proximal and distal enhancers around the close proximity looping (Figure 5b, 5d).”*

*In addition, we observed H3K27me3 peaks at the SNAI2 gene in PEO1 (Figure S7d). This also corresponds to our previous publication where the increase in H3K27me3 levels due to SNAI1 overexpression were validated by ChIP-qPCR (Sundararajan, V., Tan, M., Tan, T.Z. et al. Sci Rep 9, 8295 (2019). https://doi.org/10.1038/s41598-019-44826-8).*

17. What is the genomic resolution of the single-cell Hi-C analyses? The authors could expand significantly on the single-cell Hi-C analyses if the resolution is high enough. For example instead of using the rmsd for the cluster, the authors could use the cell-cell compaction of E-TADs and/or m-TADs, single-cell A/B compartment PC1 of these TADs, or the E/M-genes' P-E loops of the single cell Hi-C data to perform clustering and see which feature(s) generate clusters that correspond to the ground truth cell states identified by immunofluorescence.

*Response: We thank the reviewer for intricate suggestion. Unfortunately, the single cell workflow presented in this study was not optimal to call TADs from the scHi-C data due to the limited number of cells. Evidently, we did apply clustering of scHi-C data using scHiCluster (Zhou, J., Ma, J., Chen, Y., Cheng, C., Bao, B., Peng, J., ... & Ecker, J. R. (2019). Proceedings of the National Academy of Sciences, 116(28), 14011-14018). However, due to the spare matrices we were unable to get a clear distinction in the clustering of the single cells by their EMT states (Rebuttal Figure 5).*

*Rebuttal Figure 5: scHiCluster of (i) PEO1+HEYA8 single cells and (ii) OVCA429 shGRHL2 Tet-inducible cells.*

*As scHiCluster or similar scHi-C software such as scHiCexplorer were developed for scHi-C techniques used in generating the large numbers of scHi-C matrices described in Nagano et al., 2017 (Nagano T, Lubling Y, Várnai C, Dudley C, Leung W, Baran Y, Mendelson Cohen N, Wingett S, Fraser P, Tanay A. Nature. 2017 Jul 5;547(7661):61-67. doi: 10.1038/nature23001. PMID: 28682332; PMCID: PMC5567812), where they looked at the 3D genome changes during cell cycle, these studies sequenced up to thousands of single cells to allow the cells being clustered by the cell cycle phases. Those clustering algorithms fit these studies as these cell cycles phases are very different in the context of 3D genome, which was already shown in bulk studies of 3D genome changes during cell cycle. On the other hand, for scHi-C techniques described in Stevens. et al., 2017 (Stevens, T., Lando, D., Basu, S. et al. Nature 544, 59–64 (2017). https://doi.org/10.1038/nature21429) and our scHi-C workflow which applied on a smaller number of cells, structural similarity measured by RMSD was used to analyse the 3D genome structures of the single cells. Consequently, the analysis is pertaining to the scHi-C technique used and limited by the platforms applied.*

Minor points:

18. Fig. 1C: The circular shape of this plot is unnecessary and only increases the difficulty of reading it.

19. Fig. S1 is introduced after Fig. S2.

20. Page 7 line 29: "Figure S2a" should be S2b, also this is introduced after S2d. The numbering of the figures should following the order they are introduced in.

21. Page 7 line 41: "Figure 2a" should be 2b.

22. Page 8 line 10 and 12: "Figure S1b" should be S2C

23. Supple Table 2e: Title says "mesenchymal genes in 'BA" yet table says "AA (Epithelial genes, n= 64)".

24. Page 9 line 27-29: Figure numbers are wrong.

25. "Evidently, majority of the E genes remained stable in the AA/BB compartments (n = 64, 72.7%, Supplementary table 3a) across the 6 cancer cell lines (Figure 2d)." I only see five cell lines in Figure 2d. Similarly many other places mentioned 6 cell lines yet only 5 are shown in figures.

26. Page 13 line 55: "Figure 4b" should be Figure 4bc since both the E and M genes are discussed in this sentence.

27. Terms like TSS and TES were not defined.

28. Overall too many speculations are mixed with observations in the results section. I suggest moving most of the speculations to the discussions section.

*Response: We thank the reviewer for the comments highlighted from points 18-28. We have revised the manuscript accordingly.*

29. Page 18 line 46-49: "The TAD boundaries were shown to be depleted in DNA-DNA contacts in both the ensemble and single cell Hi-C matrices (Figure 6c)." Where are the single cell matrices in Fig. 6c? The left label says "based on bulk HiC".

*Response: We thank the reviewer for the comment and have removed this analysis from the revised manuscript, as we added in more data on scHi-C libraries constructed using OVCA429 shGRHL2 Tet-inducible cell line to better answer the heterogeneity of 3D genome in EMT transition.*

30. In figure 6f and S4d, which cell is in which state? The authors argue that chr10 allow clustering of cells into 3 groups that corresponds to the E, hybrid and M clusters. This is not shown in the two figures.

*Response: We apologise to the reviewer in lacking the annotation required in Figure 6. We have revised Figure 6 and added in the annotation indicating the cluster to which the single cell belongs to. We have also revised the manuscript on the clustering of r.m.s.d values of chr10 to “Clustering of the r.m.s.d values of the 3D genome structures of chr 10, where VIM is situated, showed that the genome structures in the E cluster were distinct from the Hybrid and M clusters (Figure 6d).”*

**Second round of review**

**Reviewer 1**

The authors addressed all of my concerns. The revised manuscript has been significantly improved. Particularly, the new data added in Figure 6 clearly suggests the heterogenous chromatin state and dynamic regulation of chromatin conformation during EMT. This supports the conclusions from the chromatin conformation analysis in multiple cancer cell lines.

**Reviewer 2**

Fig 1 b first panel for “Ovarian cancer”, as one of the first data presented, show the opposite result from that claimed in the text. While the text claims: “We observed that the E-scored tumours had a stronger expression of E genes and a reduced expression of the M genes in 6 cancers, namely ovarian, breast, gastric, lung, colorectal and bladder cancer”, the plot shows E-scored tumours (purple roles) had a weaker expression (blue pixels) of E genes (purple columns). It seems the gene label/the matrix could have been wrongly flipped. Similar issue may have occurred to the lung cancer matrix/gene label on the right (note the leftmost yellow M-genes are mainly expressed in the purple E tumours; similar issue for the rightmost part of the matrix). Please note many similar issues occurred in the last submission and it was the cause of a major negative comment of the reviewer in the last review. It seems none of the authors was able to carefully proofread the paper, as is a professional and ethical responsibility and requirement of each author, to ensure its accuracy and catch obvious errors like these – major figures not showing what they are said to show. The underlying concern of the reviewer is the lack of rigor and care in manuscript preparation may have also happened during the experiments and analyses given the complexity of the work, which the reviewer will not be able to help catch. Because of the repeated issue, the reviewer is ethically obliged to remind readers of this paper to take the conclusions and results with a grain of salt.

Fig S1a is said to show “These EMT genes were not frequently associated with genomic alterations (gene alteration frequency = 0.7%; Figure S1a) nor showed frequent copy number gains/losses (average copy number gain = 1%, average copy number loss = 0.3%; Figure S1a)”. Yet Fig S1a is showing the survival curves.

“Interestingly, E genes in BB and BA were re-assigned to active sub-compartments A.1.1, A.1.2, A.2.1, and A.2.2 in PEO1 without significant change in sub-compartments in HEYA8 (Figure 2c and Supplementary table 2f).” Could the authors explain why the sub-compartment assignment and the compartment assignment appear to be inconsistent in many cases? For example, some B compartment regions are assigned as sub-A compartment regions in the same cell state. The sub-A compartments should be part of the A compartment.

“Knowing that the mTADs did not show significant enrichment of repressive histone marks (Figure 3e and 3f), the frequent contact probabilities within the mTADs of the M state might suggest increased loop formation, since the mTADs in the E-like and the M-like cell lines did not differ in terms of accessibility (Figure S7a).” Besides “increased loop formation”, an alternative model would be stronger boundary blocking loop extrusion – ie, the number of loops may remain the same, but due to the stronger boundary (e.g. by CTCF), the loop stems are more homogenously positioned in the population, ie loop extrusion becomes more likely to be stopped by the boundary, rather than bypassing the boundary and generating a mix of different loop conformations in the population that cancel each other in the averaging. To distinguish these possibilities, what are the CTCF, cohesin, NIPBL, and WAPL expression levels in the E and M cells respectively?

The Hi-C results and the 3C-qPCR results still look inconsistent. Could the authors plot the bar plots like Fig. 4e for Hi-C data too for the selected genes and elements, and place that side by side with Fig 4e to show consistency, please? Could the authors mark CDH1’s data points in the box plot of Fig. 4b based on the Hi-C data, and show “increased interaction between selected E genes such as CDH1 and CDH3 with non-EMT genes in the same domain”?

**Authors Response**

**Point-by-point responses to the reviewers’ comments:**

Reviewer #1:

The authors addressed all of my concerns. The revised manuscript has been significantly improved. Particularly, the new data added in Figure 6 clearly suggests the heterogenous chromatin state and dynamic regulation of chromatin conformation during EMT. This supports the conclusions from the chromatin conformation analysis in multiple cancer cell lines.

*Response: We thank the reviewer for the positive comment. We agree with the reviewer that there exists heterogenous chromatin states and dynamic regulation of chromatin conformation during EMT. We believe that we have shown the potential in single cell technology to decipher this heterogeneity and the method presented in this study would pioneer interest for further interrogation into the epigenomic landscapes of EMT.*

Reviewer #2:

1. Fig 1 b first panel for “Ovarian cancer”, as one of the first data presented, show the opposite result from that claimed in the text. While the text claims: “We observed that the E-scored tumours had a stronger expression of E genes and a reduced expression of the M genes in 6 cancers, namely ovarian, breast, gastric, lung, colorectal and bladder cancer”, the plot shows E-scored tumours (purple roles) had a weaker expression (blue pixels) of E genes (purple columns). It seems the gene label/the matrix could have been wrongly flipped. Similar issue may have occurred to the lung cancer matrix/gene label on the right (note the leftmost yellow M-genes are mainly expressed in the purple E tumours; similar issue for the rightmost part of the matrix).

*Response: We thank the reviewer for picking this up. We have updated Figure 1b accordingly with the correct labels of the E/M tumours, as well as the E and M genes. The annotation for the colour panel indicating the EM genes was misaligned during the proofreading process. We have made it consistent that E is always presented in purple and M is always presented in yellow. We apologize to the reviewer for this confusion.*

Please note many similar issues occurred in the last submission and it was the cause of a major negative comment of the reviewer in the last review. It seems none of the authors was able to carefully proofread the paper, as is a professional and ethical responsibility and requirement of each author, to ensure its accuracy and catch obvious errors like these – major figures not showing what they are said to show. The underlying concern of the reviewer is the lack of rigor and care in manuscript preparation may have also happened during the experiments and analyses given the complexity of the work, which the reviewer will not be able to help catch. Because of the repeated issue, the reviewer is ethically obliged to remind readers of this paper to take the conclusions and results with a grain of salt.

*Response: We thank the reviewer for the comment, and we truly apologize for the editorial errors in the previous revised manuscript. We had heeded the advice of the reviewer with great importance during the first revision and had carried out multiple rounds of proofreading. As a cloud sharing platform was used to share documents among the authors, there was a lapse in the proofreading process when carried out across multiple authors due to conflicted copies being saved. Hence, errors were carried forward unknowingly. Therefore, during the second revision, we have made adjustment in our co-working format to ensure that the duplication of different versions of the revised manuscript would not occur.*

*We strongly agree with the reviewer that every author has a professional and ethical responsibility to ensure that a clear and accurate publication is being presented. Being a well-established group with recognized publication records, we also strive our best to ensure high standards are maintained. This standard has never been compromised. We also agree with the reviewer that there exists high dimension of complexity in the experiments and the analysis of this work. We have high confidence that the data analysis is reproducible, and the dataset will be released onto the public domain for the scientific community to access. Given that this review process will also be made transparent, we believe that the readers would be able to witness the transparency and forthcoming we have demonstrated during the revisions.*

Fig S1a is said to show “These EMT genes were not frequently associated with genomic alterations (gene alteration frequency = 0.7%; Figure S1a) nor showed frequent copy number gains/losses (average copy number gain = 1%, average copy number loss = 0.3%; Figure S1a)”. Yet Fig S1a is showing the survival curves.

*Response: We have amended Figure S1. Figure S1a and S1b are now showing the correct annotations.*

2. “Interestingly, E genes in BB and BA were re-assigned to active sub-compartments A.1.1, A.1.2, A.2.1, and A.2.2 in PEO1 without significant change in sub-compartments in HEYA8 (Figure 2c and Supplementary table 2f).” Could the authors explain why the sub-compartment assignment and the compartment assignment appear to be inconsistent in many cases? For example, some B compartment regions are assigned as sub-A compartment regions in the same cell state. The sub-A compartments should be part of the A compartment.

*Response: We thank the reviewer for the comment. The calling of the sub-compartments and the compartments were done by using different computation methods. The sub-compartments were called using CALDER (https://github.com/CSOgroup/CALDER) while the main chromosomal compartments were derived using HOMER (http://homer.ucsd.edu/homer/). Because HOMER does not compute the sub-compartments, we had to use an alternative software such as CALDER to predict the sub-compartments from the Hi-C contacts. Therefore, it is not ideal to perform a stringent head-to-head comparison between them. Nevertheless, there was some degree of overlap of the compartment/sub-compartment calls between these 2 methods. As most of the compartmental analysis in this work was based on the main compartments, we stuck to using HOMER derived main chromosomal compartments for all the 5 cancer cell lines.*

3. “Knowing that the mTADs did not show significant enrichment of repressive histone marks (Figure 3e and 3f), the frequent contact probabilities within the mTADs of the M state might suggest increased loop formation, since the mTADs in the E-like and the M-like cell lines did not differ in terms of accessibility (Figure S7a).” Besides “increased loop formation”, an alternative model would be stronger boundary blocking loop extrusion – ie, the number of loops may remain the same, but due to the stronger boundary (e.g. by CTCF), the loop stems are more homogenously positioned in the population, ie loop extrusion becomes more likely to be stopped by the boundary, rather than bypassing the boundary and generating a mix of different loop conformations in the population that cancel each other in the averaging. To distinguish these possibilities, what are the CTCF, cohesin, NIPBL, and WAPL expression levels in the E and M cells respectively?

*Response: We thank the reviewer for the suggestion in considering the loop formation models. We observed that the expression levels of CTCF, SMC3, NIPBL and WAPL were similar across the 5 cancer cell lines (Rebuttal Figure 1, below; Figure S6b, revised manuscript). This suggests that there might not be a change of the boundary “strength” due to the expression differences.*

*We have thus revised the manuscript “Knowing that the mTADs did not show significant enrichment of repressive histone marks (Figure 3e and 3f), the frequent contact probabilities within the mTADs of the M state might suggest increased loop formation, since the mTADs in the E-like and the M-like cell lines did not differ in terms of accessibility (Figure S6a). In addition, we observed similar levels of CTCF and cohesin complex proteins across the E-like and M-like cell lines (Figure S6b) thus implying that the boundaries of the mTADs do not play a significant role towards the changes in contact probabilities within the TADs.”*

*Rebuttal Figure 1. RNA-seq log2 expression (y-axis) of CTCF, SMC3, NIPBL and WAPL in MCF7 (dark purple), PEO1 (light purple), PANC1 (pale yellow), A549 (light yellow), HEYA8 (dark yellow).*

4. The Hi-C results and the 3C-qPCR results still look inconsistent. Could the authors plot the bar plots like Fig. 4e for Hi-C data too for the selected genes and elements, and place that side by side with Fig 4e to show consistency, please? Could the authors mark CDH1’s data points in the box plot of Fig. 4b based on the Hi-C data, and show “increased interaction between selected E genes such as CDH1 and CDH3 with non-EMT genes in the same domain”?

*Response: We thank the reviewer for the comment and have thus showed the observed/expected values extracted from the Hi-C data in PEO1 and HEYA8, alongside the 3C-qPCR results (Rebuttal Figure 2a and 2b, below; Figure 4f, revised manuscript). In the Hi-C data, we observed higher interaction frequencies between CDH1 and CDH3 in PEO1 albeit not being significant, while there was no significant difference in the Hi-C data at the interacting loci: CDH1-ZFP90, CDH3-ZFP90 and CDH1 TAD boundary. On the other hand, at the CDH2 TAD, we observed significantly higher interaction frequencies in HEYA8 at the interacting loci of CDH2 TSS-intron, CDH2 TES-enhancer, which is consistent with the 3C-qPCR results. This is due to the nature of 3C-qPCR being a direct and more quantitative approach at one-to-one interactions.*

*We have included the following figure in the revised manuscript as Figure 4f and added the following text in the manuscript. “In agreement with the 3C-qPCR observations, Hi-C data at the CDH2 TAD showed higher interaction frequencies in HEYA8 at the interacting loci of CDH2 TSS-intron, CDH2 TES-enhancer (Figure 4f). Albeit not being significant, we did observe higher interaction between CDH1 and CDH3 in the Hi-C data of PEO1 as compared to HEYA8 (Figure 4f).”*

*We have also annotated the data points in the Hi-C aggregate matrices that were extracted for the boxplots presented in Figure 4b and 4c. In Figure 4d, we use grey arrows to annotate the interacting loci as assessed by 3C-qPCR in the triangular Hi-C matrix plots.*

*Rebuttal Figure 2. (a) Bar plot depicting Hi-C observed/expected values (y-axis) between interacting loci (x-axis) in CDH1 TAD, in PEO1 (black) and HEYA8 (gray). The interacting loci at the CDH1 TAD are as follows: “CDH1-CDH3” (CDH1 TSS to CDH3 TSS), “CDH3-ZFP90” (CDH3 TSS to ZFP90 TSS), “CDH1- ZFP90” (CDH1 TSS to ZFP90 TSS) and “CDH1 TAD boundary” (CDH1 TAD 5’boundary to 3’boundary). (b) Bar plot depicting Hi-C observed/expected values (y-axis) between interacting loci (x-axis) in CDH2 TAD, in PEO1 (black) and HEYA8 (gray). The interacting loci at the CDH2 TAD are as follows: “CDH2 TSS-intron” (CDH2 TSS to CDH2 intronic enhancer), “CDH2 TES-Enhancer” (CDH2 TES to CDH2 upstream enhancer) and “CDH2 TAD boundary” (CDH2 TAD 5’boundary to 3’boundary). Student T-test were used for statistical analyses. *p<0.05.*

**Third round of review**

**Reviewer 2**

The authors have addressed my previous concerns.
